# Towards the development of a CRISPR-Cas9 based kill switch for *Saccharomyces cerevisiae*

**DOI:** 10.1186/s12934-026-02959-2

**Published:** 2026-02-20

**Authors:** Pavithra Umashankar, Bohyun Choi, Yvonne Nygård

**Affiliations:** 1 Department of Life Sciences, Division of Industrial Biotechnology, Chalmers University of Technology, 412 96 Gothenburg, Sweden; 2 VTT Technical Research Centre of Finland, Espoo, Finland

**Keywords:** Kill switch, Genetic circuit, CRISPR-Cas9, Yeast, Population control, Inactivation, Escape

## Abstract

**Background:**

Advancements in synthetic genetic circuits have enabled programmable and condition-dependent control of microbial cell growth. CRISPR-Cas9-based kill switches, genetic systems that program cells to lose viability in response to specific conditions, have recently been demonstrated for bacterial cell factories but not yet in yeast.

**Results:**

In this study, we present a foundational demonstration for a CRISPR-based *ki*ll *s*witch in *Saccharomyces cerevisiae,* CRISPR KiSS. The CRISPR KiSS employs inducible CRISPR targeting essential genes to elicit growth inhibition. The activation of the KiSS system is achieved through conditional expression of a guide RNA (gRNA) upon anhydrotetracycline (ATc) induction, thereby activating CRISPR-mediated gene disruption. We demonstrate that targeting the essential genes (*ERG13, PGA3, TPI1* or *CDC19)* leads to severe growth inhibition upon ATc induction. Still, the current set up does not allow complete killing of the cells due to system inactivation, e.g. escape from CRISPR based cutting. We studied reasons for system inactivation and substantially improved the system by simultaneous expression of two different gRNAs. Sequencing escape mutants revealed mutations in both the gRNA sequences and target genes as potential sources of system inactivation.

**Conclusions:**

This work highlights the potential of harnessing a CRISPR-based kill switch in *S. cerevisiae*. Cells expressing the system were able to escape growth inhibition through mutations and further optimization of the KiSS system is still needed for it to be used in various cell factory applications.

**Supplementary Information:**

The online version contains supplementary material available at 10.1186/s12934-026-02959-2.

## Background

*Saccharomyces cerevisiae* is among the most widely exploited cell factories of today. A wide selection of genetic circuits and other synthetic biology tools have already been developed for this and other yeasts. Recently, genetic circuits for controlling cell viability have emerged. Kill switches are artificial genetic devices that induce cell death under specific environmental conditions [1]. For biocontainment, e.g. when there is a need to conditionally eliminate a population, efficiency and robustness of the kill switch become imperative. While achieving such a stringent kill switch can be challenging, less stringent, growth-controlling kill switches can still be effectively applied in closed bioproduction settings. The capability to control cell growth is attractive for bioproduction purposes, e.g. when production is to be decoupled from cell growth or in a co-culture where population dynamics are to be altered.

Several types of kill switches have already been demonstrated in *S. cerevisiae,* including systems for on-demand cutting of DNA using nucleases [2], restriction enzymes [3] or conditional expression of toxin-antitoxin genes [4]. Controlling the regulation of essential genes—either through conditional removal, controlling transcription or translation, or making the stability or function of essential proteins conditional, is another kill switch approach [5]. Simultaneous introduction of multiple means for controlling regulation has been shown to increase the efficiency of such systems. Transcriptional and recombinational control of essential histone genes, controlled by the addition of estradiol proved to be an efficient way to control cell viability in *S. cerevisiae* [6]. In a recent study, an estrogen receptor-derived destabilizing domain was fused to essential genes, resulting in conditional post-translational control of essential genes and subsequent in vivo half-life of proteins, resulting in stringent biocontainment [7]. A similar approach was used for de-coupling growth from production by conditionally degrading essential proteins targeted to the bacterial ClpXP proteasome expressed in *S. cerevisiae* [8]*.*

Lately, CRISPR/Cas-based kill switches have been emerging in bacterial systems [9–11]. In such systems, the Cas endonuclease recognizes a target sequence and introduces a double stranded break (DSB), which endogenously triggers cell death [12]. Caliando et al. (2015) developed a targeted DNA degradation device in *E. coli* that upon arabinose induction selectively degraded DNA at specific target sites, resulting in cell death [9]. Rottinghaus et al. [11] developed a two-input kill switch utilizing CRISPR-Cas9 to induce DSBs in *E. coli*. In this system anhydrotetracycline (ATc) served as the first input to initiate cell death, while a second level of control was based on temperature-dependent de-repression of the kill switch [11]. Recently, a CRISPR-Cas9 based kill switch in which cell death was induced by targeting highly repetitive genomic elements was demonstrated for *Pseudomonas putida* [10]. These systems in bacteria highlight that CRISPR circuits can be combined with inducible or multi-input regulatory modules, allowing tight, tunable, and programmable control over cell viability. While the mentioned systems were shown to work and escape frequency of strains with CRISPR-based kill switches were improved during the studies, system inactivation remains a big challenge.

Compared to toxin-antitoxin based-, auxotrophy based-, and essentiality-based kill switch systems which rely on toxin expression or metabolic dependencies, CRISPR-based kill switches offer distinct advantages. They enable sequence-specific degradation of the genetic material, and provide a programmable, modular framework [9]. By directly targeting and degrading DNA, CRISPR-based kill switches can limit horizontal gene transfer, overcoming a major limitation of many existing kill switch approaches [13]. CRISPR-based kill switches in yeast are yet to be explored.

Here, we present an inducible CRISPR-Cas9 based kill switch in *S. cerevisiae* (CRISPR KiSS) that inhibits cell growth by expression of Cas9 and essential gene-targeting gRNAs in response to ATc. In this design, gRNAs targeting essential genes (*ERG13, PGA3, CDC19,* and *TPI1*) were expressed by an inducible RNA polymerase III promoter. Erg13 is essential for ergosterol biosynthesis [14], Pga3 is linked to protein trafficking and lifespan regulation [15], Cdc19 is rate-limiting in glycolysis [16], and Tpi1 is essential for central carbon metabolism [17]. Targeting *ERG13, PGA3, CDC19* or *TPI1* with the KiSS system led to notable growth inhibition upon ATc induction. Notably, the simultaneous expression of two gRNAs led to stronger growth inhibition compared to single gRNA constructs and to a lower escape frequency. Further optimization is expected to improve the system and pave the way for various applications where population control is beneficial.

## Methods

### Strains, media composition, and cultivation

Electrocompetent *E. coli* TOP 10 cells were used for cloning, and transformed cells were selected using appropriate antibiotics. *E. coli* strains were cultivated in lysogeny broth (LB), composed of 5 g/L yeast extract, 10 g/L peptone from casein, and 10 g/L NaCl, when needed solidified by addition of 15 g/L agar–agar. *S. cerevisiae* CEN.PK 113-5D [18] was used as the parental strain for all KiSS strains constructed in this study (Table 1). Yeast cells were cultivated in YPD medium (20 g/L bacterial peptone, 10 g/L yeast extract, 20 g/L glucose) or synthetic defined (SD) medium lacking uracil (0.77 g/L complete supplement mixture [CSM] without uracil, 6.9 g/L yeast nitrogen base without amino acids and ammonium sulfate [YNB w/o AA and ammonium sulfate], 5 g/L, urea, 20 g/L glucose, pH 5.5), and when required supplemented with 200 µg/mL G418 (geneticin) for plasmid maintenance.

**Table 1:** Strains used in the study

Name	Genotype	Description	Parental strain
CEN.PK 113-5D	*MATa MAL2-8c ura3-52 HIS3 LEU2 TRP1*		
CEN.PK_Cas9_FPs	CEN.PK 113-5D HO::pPGK1-Cas9-PGK1t-pPCCW12-mCherry-SSA1t-pRPL18B-mTurquoise2-ENO2t	Basal strain expressing Cas9 and two fluorescent proteins	CEN.PK 113-5D
mCherry_S1	CEN.PK_Cas9_FPs pRPR1_tetO-sgRNA_mCherry-tSNR52-pTDH3-tetR-ScTDH1t	KiSS strain with sgRNA targeting *mCherry*	CEN.PK_Cas9_FPs
mTurquoise2_S1	CEN.PK_Cas9_FPs pRPR1_tetO-sgRNA-tSNR52-pTDH3-tetR-ScTDH1t	KiSS strain with sgRNA targeting *mTurquoise2*	CEN.PK_Cas9_FPs
ERG13_S1	CEN.PK_Cas9_FPs pRPR1_tetO-ERG13_sgRNA1-tSNR52-pTDH3-tetR-ScTDH1t	KiSS strain with sgRNA #1 targeting *ERG13*	CEN.PK_Cas9_FPs
PGA3_ S1	CEN.PK_Cas9_FPs pRPR1_tetO-PGA3_sgRNA1-tSNR52-pTDH3-tetR-ScTDH1t	KiSS strain with sgRNA #1 targeting *PGA3*	CEN.PK_Cas9_FPs
ERG13_S4	CEN.PK_Cas9_FPs pRPR1_tetO-ERG13_sgRNA4-tSNR52-pTDH3-tetR-ScTDH1t	KiSS strain with sgRNA #4 targeting *ERG13*	CEN.PK_Cas9_FPs
ERG13_S4	CEN.PK_Cas9_FPs pRPR1_tetO-ERG13_sgRNA4-tSNR52-pTDH3-tetR-ScTDH1t	KiSS strain with sgRNA #4 targeting *ERG13*	CEN.PK_Cas9_FPs
ERG13_S5	CEN.PK_Cas9_FPs pRPR1_tetO-ERG13_sgRNA5-tSNR52-pTDH3-tetR-ScTDH1t	KiSS strain with sgRNA #5 targeting *ERG13*	CEN.PK_Cas9_FPs
ERG13_S6	CEN.PK_Cas9_FPs pRPR1_tetO-ERG13_sgRNA6-tSNR52-pTDH3-tetR-ScTDH1t	KiSS strain with sgRNA #6 targeting *ERG13*	CEN.PK_Cas9_FPs
TPI1_S1	CEN.PK_Cas9_FPs pRPR1_tetO-TPI1_sgRNA1-tSNR52-pTDH3-tetR-ScTDH1t	KiSS strain with sgRNA #1 targeting *TPI1*	CEN.PK_Cas9_FPs
TPI1_S2	CEN.PK_Cas9_FPs pRPR1_tetO-TPI1_sgRNA2-tSNR52-pTDH3-tetR-ScTDH1t	KiSS strain with sgRNA #2 targeting *TPI1*	CEN.PK_Cas9_FPs
CDC19_S1	CEN.PK_Cas9_FPs pRPR1_tetO-CDC19_sgRNA1-tSNR52-pTDH3-tetR-ScTDH1t	KiSS strain with sgRNA #1 targeting *CDC19*	CEN.PK_Cas9_FPs
CDC19_S2	CEN.PK_Cas9_FPs pRPR1_tetO-CDC19_sgRNA2-tSNR52-pTDH3-tetR-ScTDH1t	KiSS strain with sgRNA #2 targeting *CDC19*	CEN.PK_Cas9_FPs
PGA3_S1_ERG13_S6	CEN.PK_Cas9_FPs pRPR1_tetO-PGA3_sgRNA1-tSNR52-pTDH3-tetR-ScTDH1t pRPR1_tetO-ERG13_sgRNA6-tSNR52-pTDH3-tetR-ScTDH1t	KiSS strain with sgRNA #1 and sgRNA #6 targeting *PGA3* and *ERG13*	CEN.PK_Cas9_FPs
ERG13_S4_ERG13_S6	CEN.PK_Cas9_FPs pRPR1_tetO-ERG13_sgRNA4-tSNR52-pTDH3-tetR-ScTDH1t pRPR1_tetO-ERG13_sgRNA6-tSNR52-pTDH3-tetR-ScTDH1t	KiSS strain with sgRNA #4 and sgRNA #6 targeting *ERG13*	CEN.PK_Cas9_FPs
Control	CEN.PK_Cas9_FPs pRPR1_tetO-no_sgRNA-tSNR52-pTDH3-tetR-ScTDH1t	KiSS strain with no sgRNA	CEN.PK_Cas9_FPs

### Design of constructs and cloning

Unless otherwise specified, all the designed constructs were assembled following the MoClo Yeast Toolkit (Addgene #1000000061) instructions [19]. The sequences of all primers and gRNA sequences are listed in Table S1, and the plasmids used are detailed in Table S2. All primers and gene fragments used were purchased from Eurofins Genomics (Germany). PCR components, restriction enzymes, and ligation components were purchased from Thermo Scientific (USA). Plasmids were purified using GeneJET Plasmid Miniprep Kit (Thermo Scientific, USA). The NEBuilder® HiFi DNA Assembly Master mix (New England Biolabs, USA) was used for Gibson assembly reactions according to the manufacturer’s protocol.

The MoClo kit was expanded with level-0 vector of the tetracycline operator (tetO) modified *RPR1* RNA polymerase III promoter, the P_RPR1-tetO_ sequence was purchased as a gene fragment and when inserted into the pYTK001 vector named pPU0_001. The level-0 part containing a tetracycline-regulated repressor (tetR) was constructed by PCR amplification of the corresponding gene fragments from pRS416-dCas9-Mxi1 + TetR + pRPR1(TetO)-NotI-gRNA (Addgene #73796) [20] resulting in plasmid pPU0_004. The level-0 genetic part for sgRNA expression, pPU0_006, was constructed by PCR amplification of the sgRNA cassette (HDV ribozyme prior to a placeholder for the sgRNA, followed by the sgRNA scaffold and the *SNR53* terminator) from the pYTK050 plasmid.

To facilitate integration, a level-1 backbone plasmid designated as pPU1_007 was constructed. This plasmid includes homology arms for the *HO* locus, the yeast *URA3* selection cassette, and a bacterial GFP-dropout construct. Furthermore, fluorescent protein expression plasmids, pPU1_008 and pPU1_009 containing the cyan fluorescent protein-encoding gene *mTurquoise2* and the red fluorescent protein-encoding gene *mCherry* (pMM0_12, [21]) expressed under the *RPL18b* and *PCCW12* promoters, respectively, were constructed. These constructs also contained the *ENO2* and *SSA1* terminators, respectively. pPU1_0010 was constructed for Cas9 expression, using the *PGK1* promoter and terminator. The level-1 expression backbone plasmid, pPU1_016 was constructed with a G418 resistance cassette for selection in yeast.

The final, level-2 integration vector, pPU2_020 was assembled by combining plasmids pPU1_007 to pPU1_010. The level-2 expression vector, pPU2_017 was constructed by PCR amplification of the transcription units of the MoClo level 1 vectors for expressing the sgRNA(s) under P_RPR1-TetO_ (pPU0_001), the repressor cassette (pPU0_004), the sgRNA cassette (pPU0_006) and pPU1_016 with appropriate overhangs and assembled using Gibson assembly. The sgRNAs targeting essential genes were chosen using CRISPR-ERA [22] and CHOPCHOP [23]. For sgRNA expression plasmids, the sgRNAs sequences were assembled in pPU2_017 after BsmBI digestion, resulting in sgRNA expression plasmids pPU2_021 to pPU2_032. The control plasmid was constructed by inserting a 20 bp non-targeting sequence, resulting in pPU2_033. The sgRNA sequences with compatible BsmBI sites were purchased as primers and hybridized by boiling followed by a gradual decrease in temperature. For the construction of dual gRNAs expression plasmids, the PGA3_S1 expression cassette was amplified from pPU2_024 with PCR primers containing NotI-flanked ends and inserted into NotI digested pPU2_027 containing the ERG13_S6 module, generating the PGA3_S1_ERG13_S6 construct, named pPU2_034. Similarly, the ERG13_S4 cassette was subcloned from pPU2_025 into pPU2_027 harboring ERG13_S6, to obtain the ERG13_S4_ERG13_S6 construct, named pPU2_035. Constructs assembled were confirmed with colony PCR and restriction digestion.

### Strain construction

Yeast strains were transformed using the Gietz protocol [24]. The genetic constructs were integrated using the CRISPR/Cas9 technology and plasmid YN2_1_IL50_HOlocus (Addgene # 194,426) [25], containing a Cas9 expression cassette and an sgRNA targeting the *HO* locus (GCTCCAGCATTATAGCATGC). The pYN2_1_IL50_HOlocus plasmid was co-transformed with NotI digested pPU2_020. Correct integration of the cassette was verified by colony PCR. The pYN2_1_IL50_HOlocus plasmid was cured from the strain, CEN.PK_Cas9_FPs, prior to transformation with either single- or dual gRNAs expression plasmids.

### Growth characterization in microtiter plates

All yeast pre-cultures except escape frequency determination were inoculated from individual colonies grown on solid medium and grown overnight in 3 mL SD-URA medium supplemented with G418, in 14 mL culture tubes. The cells of the pre-cultures were then collected, washed with fresh SD media, and used for inoculation, each culture originating from a separate colony, resulting in replicate cultures. For the pre-cultures used for escape frequency determination, 24 colonies grown on solid medium, of each strain, were inoculated in 250 µL SD-URA medium supplemented with G418, in 96-well plates. After overnight culture, 12 of the pre-cultures were collected, washed with fresh SD media, and used for inoculation. Microtiter cultivation was performed in 96-well plates, where each well containing 250 µL SD -URA medium was inoculated to an initial optical density at 600 nm (OD_600_) of 0.1. For induction studies, 0, 5 or 10 µg/mL of ATc was added to the medium either at the start or during the cultivation. The stock solution of 5 mg ATc/mL in DMSO was diluted with DMSO to obtain the desired concentrations. The 96-well plates were shaken at 250 rpm in a Growth profiler 960 device (Enzyscreen, Netherlands). All the yeast cultures were carried out at 30 °C. The growth data was collected as green values and then converted to OD_600_ according to a standard curve, following the instructions of the Growth Profiler. The green values obtained for cultures with ATc were normalized against blank media containing the corresponding ATc concentrations without cell inoculation. All experiments were performed independently in at least three replicates.

### Fluorescence microscopy and image processing

Yeast strains expressing *mCherry* and *mTurquoise2* were cultured in 3 ml SD -URA medium in 14 mL round culture tubes for 16 h. Then 100 µL of culture was collected and the cells were washed with 200 µl of PBS buffer prior to microscopy. The fluorescence expression was visualized using a LEICA DMi8 microscopy (LEICA, Germany) equipped with a LEICA DFC7000 T Color CCD Microscope Camera and FITC filter set. The mCherry signal was visualized using a 540/580 nm (excitation) and 592/668 nm (emission) filter whereas the 426/446 nm (excitation) and 460/500 nm (emission) filter set were used for mTurquoise2 visualization. The exposure time was set to 50 ms. The images were resized and cropped using LEICA application suite X (LEICA, Germany).

### Cell viability and FACS analysis

The *LIVE/DEAD* Funga*Light* Yeast Viability Kit (Invitrogen, USA) was used to monitor the viability of the strains. Yeast cells were stained with fluorescence dyes composed of SYTO™ 9 (green) and Propidium iodide (PI; red). SYTO™ 9 stained all yeast cells within the population, labeling both LIVE and DEAD cells with green fluorescence. In contrast, only yeast cells with damaged membranes were stained red by the PI, causing a reduction of green fluorescence from SYTO™ 9. Therefore, dots in the high PI intensity zone (in the order of 10^6^–10^7^) of the flow cytogram represent non-viable cells, and the rest of the dots represent viable cells. All strains expressed *mCherry* (fluorescence in the order of 104–106) but an increased red fluorescence intensity was measured for the non-viable cells. Samples were collected from the cultures used for the escape frequency determination (Fig. S6), after 24 (control), 48 (PGA3_S1 #3) or 54 (PGA3_S1_ERG13_S6 #3) h of cultivation, when the cells had resumed to grow, at mid-exponential phase. Samples were prepared following the manufacturer’s manual. In brief, 1 ml of collected yeast cells (1 × 10^7^) were centrifuged and washed with 1 mL of 1 X phosphate-buffered saline (PBS) buffer (Sigma Aldrich, USA). The Funga*Light* solution mix, which contained 3.34 mM SYTO™ 9 and 20 mM PI solution, was mixed with 1 mL of yeast cells at a concentration of 1 × 10^6^. Samples were incubated at room temperature, protected from light for 20 min, after which the stained cells were analyzed using a SONY SH800 Cell Sorter (SONY, Japan) equipped with an air-cooled 15 mW argon ion laser (488 nm), using standard settings of the emission filters. A total of 100,000 cells were counted separately, based on the respective wavelength using the Cell Sorter Software Version 2.1.7 (SONY, Japan). The data collected were further analyzed and modified using FlowJo v 10.0 (Becton, Dickinson & Company, USA).

### Statistical analysis

The specific growth rate (µ) for each strain was calculated from the OD_600_ measurements, at consecutive timepoints of the growth curves. The maximum specific growth rate (µmax) was defined as the highest value observed in the line fitting the exponential growth phase. The generation time and lag phase length of each strain was calculated using the spline curve plot in SigmaPlot 12.0 (Systat Software Inc., USA). The first immediately increased green values were considered an optical sensing error and thus excluded from analysis. Statistical differences among strains and control were calculated by student’s t-test in SigmaPlot 12.0 (Systat Software Inc., USA). Statistical significance was established at *p* < 0.05 and marked by * *p* < 0.05, ** *p* < 0.01, and *** *p* < 0.001.

## Results

### Development of the KiSS system and characterization of KiSS strains

**Fig. 1:**
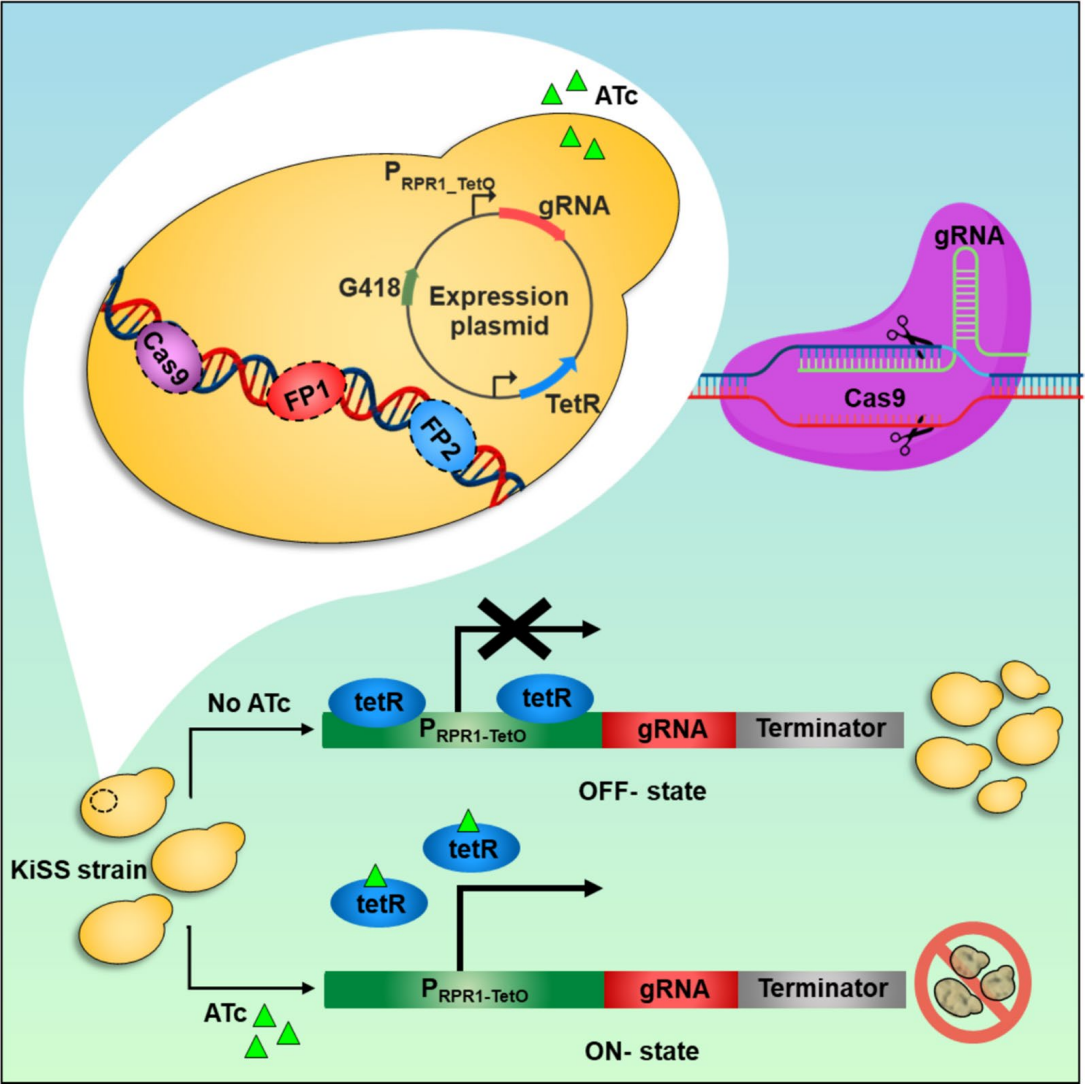
Schematic representation of the CRISPR KiSS system. Cas9 and fluorescent protein (FP1: *mCherry* and FP2: *mTurquoise2*) expression cassettes were integrated into CEN.PK 113-5D. The strains also contained a plasmid for expressing gRNA(s) under the ATc-inducible P_RPR1-tetO_ promoter and a constitutively expressed, tetracycline-regulated repressor (tetR). In the absence of ATc, tetR should repress the KiSS system. Upon addition of ATc, tetR is unable to bind to its promoter, leading to the expression of the gRNA. Cleavage of essential genes by the Cas9-gRNA complex should be detrimental for the cells.

The inducible CRISPR KiSS system consists of a genome-integrated Cas9 expression cassette combined with plasmid-based expression of gRNAs (Fig. 1). The gRNA expression is controlled by P_RPR1-tetO_, an RNA polymerase III promoter with an embedded tetracycline operator (tetO). The basal strain, *S. cerevisiae* CEN.PK_Cas9_FPs, also contains genome-integrated cassettes for expression of *mCherry* and *mTurquoise2.* Multiple gRNAs targeting *ERG13*, *PGA3, TPI1, CDC19, URA3*, *mCherry* or *mTurquoise2* were tested.

**Fig. 2:**
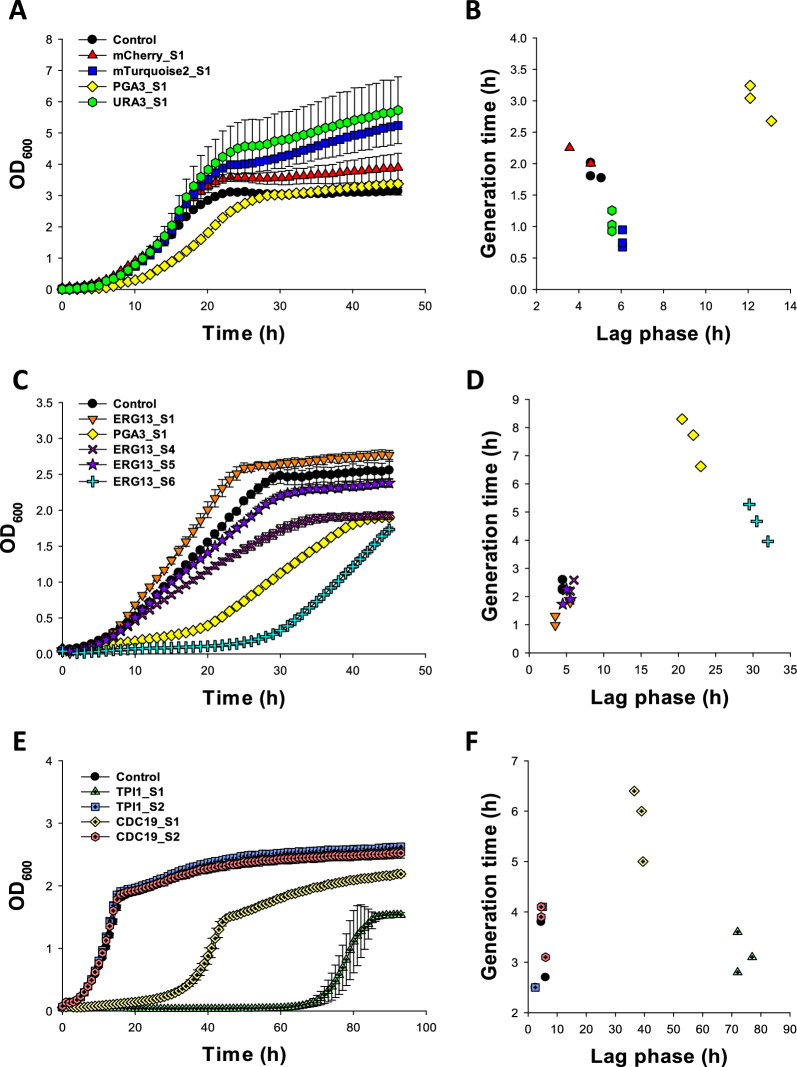
Growth profiles (**A**, **C** and **E**) and the generation time at the maximum specific growth rate plotted against the lag time (**B**, **D** and **F**) of a representative set of KISS strains: control (black circle; ), mCherry_S1 (red triangle; ), mTurquoise2_S1 (blue square; ), PGA3_S1 (yellow diamond; ), URA3_S1 (green hexagon; ), ERG13_S1 (orange triangle downwards;  ), ERG13_S4 pink cross; ), ERG13_S5 (filled purple star; ), ERG13_S6 (cyan plus; ), TPI1_S1 (light green cross-hair triangle; ), TPI1_S2 (light blue cross-hair square; ), CDC19_S1 (light yellow cross-hair diamond; ), and CDC19_S2 (light red cross-hair hexagon; ). The strains were cultured in SD-URA medium supplemented with G418 (**A**, **B**) or SD-URA medium supplemented with G418 and 10 µg/mL ATc (**C**–**F**). Values shown are the means of three independent experiments, and the error bars indicate the standard error.

To evaluate the impact of the KiSS system on the strain growth, a set of strains were cultured in SD-URA medium supplemented with G418. In medium lacking the inducer for the kill switch, ATc, all strains except PGA3_S1 (Fig. 2A, B), ERG13_S6 (Figure S3A), TPI1_S1 and CDC19_S1 (Figure S3B) showed rather similar growth patterns. The strain targeting *URA3*, the gene that was also used for cloning and thus present in two copies in the genome, showed no significant (*p* > 0.05) difference in growth compared to the control strain that had no gRNA (Fig. 2A). In the absence of ATc, the PGA3_S1 exhibited growth inhibition compared to other strains (*p* = 0.001), including an approximately 2.6-fold prolonged lag phase length compared to the control strain (Fig. 2B). Similarly, TPI1_S1 and CDC19_S1 (Figure S3B) displayed approximately 6.3- and 5.1-fold prolonged lag phases, compared to the control strain. Remarkably, the introduction of plasmids with gRNAs targeting either of the two fluorescent protein encoding genes, *mCherry* and *mTurquoise2*, instantly led to silencing the fluorescence of the strains, even in the absence of ATc (Figure S1).

To verify the inducibility of the KiSS system, the growth of the strains was monitored in SD medium supplemented with 0, 5, or 10 µg/mL ATc. As the gRNA chosen has been shown to largely affect the efficiency of CRISPR/Cas systems [23], five different single gRNAs targeting *ERG13* or *URA3* were designed. All strains with gRNAs targeting *URA3* (found in two copies in the genome) had growth profiles similar to the control strain (Figure S2; data for one of the strains shown). The growth of the strains in medium lacking ATc was similar (ERG13_S1, TPI1_S2), slightly repressed (ERG13_S4, ERG13_S5 and CDC19_S2) or even highly repressed (PGA3_S1, ERG13_S6, TPI1_S1, and CDC19_S1) compared to the control strain (Figure S3). At 5 µg/mL ATc, the effects were smaller; the PGA3_S1 strain showed an approximately 2.6-fold increased generation time, and a three times prolonged lag phase length compared to the control strain (Figure S2). At 10 µg/mL ATc severe growth repression (*p* < 0.001) was observed for PGA3_S1 and ERG13_S6 (lag phase increased by 20—32 h) whereas the control strain, ERG13_S1, ERG13_S4, and ERG13_S5 showed similar lag phases and generation times (Fig. 2C, D). Similarly, TPI1_S1 and CDC19_S1 had lag phases increased by 36 or 70 h, whereas TPI1_S2, and CDC19_S2 showed similar lag phases and generation times as the control strain (Fig. 2E, F).The URA3_S1 strain showed a growth profile similar to that of the control strain (generation time of 1.3 h and a lag phase length of 3.8 h, *p* > 0.05) at all ATc concentrations and even higher biomass at the end of cultures at 0 (Fig. 2A) and 5 µg/mL ATc (Fig. S2).

### Instability and mutations observed in the KiSS strains

**Fig. 3:**
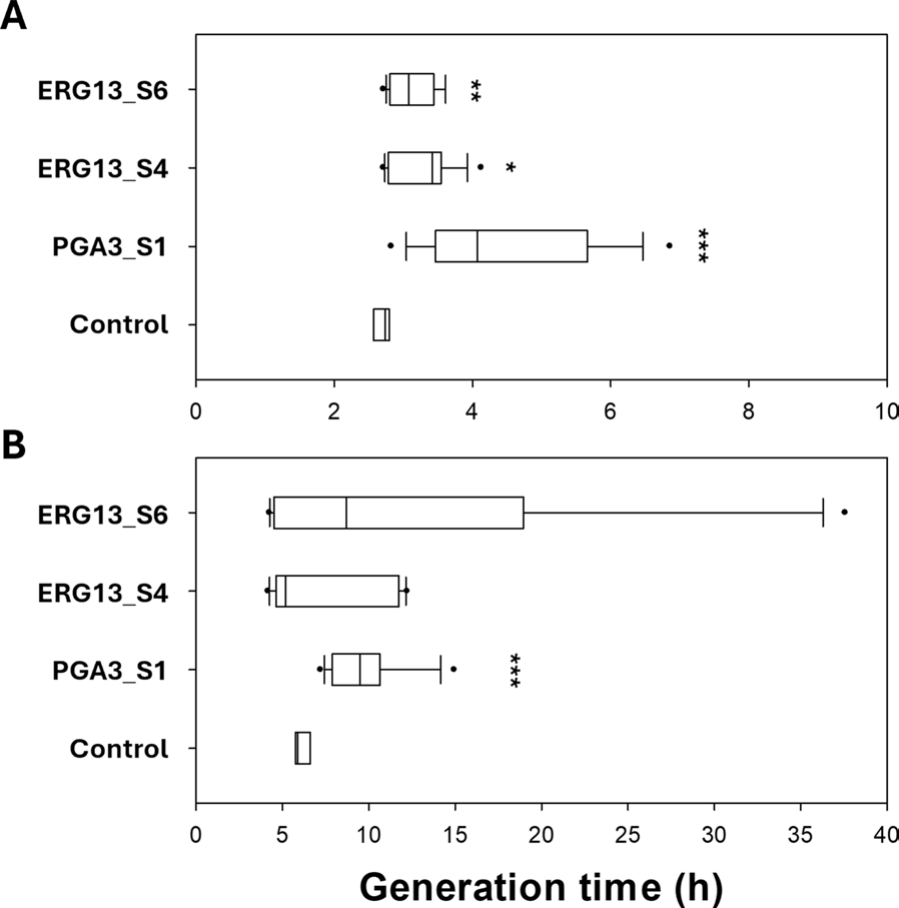
Box plots representing the generation time at the maximum specific growth rate of the control strain, PGA3_S1, ERG13_S4, and ERG13_S6 in SD—URA medium supplemented with G418 and 0 (**A**) or 10 (**B**) µg/mL ATc, added after 7 h of cultivation. The cultures were inoculated from different individual colonies (n = 9) and carried out for 48 h. A student’s t-test was used for statistical analysis, * indicates *p* < 0.05, ** *p* < 0.01, and *** *p* < 0.001.

Different growth patterns were observed across experimental replicates. To investigate this, three independent cultures of PGA3_S1, ERG13_S4, and ERG13_S6 were cultivated at 0 and 10 µg/mL ATc (Figs. 3 and S4). Two out of the three cultures of each KiSS strain exhibited a severe growth inhibition at 10 µg/mL ATc whereas one culture grew as the control. The variations in generation time among the cultures were more severe at 10 µg/mL ATc than in medium lacking ATc (Fig. 3B).

To investigate the cause of instability (resumed growth) of the KiSS strains, the target genes and the gRNA expression modules of the mCherry_S1, mTurquoise2_S1, URA3_S1, PGA3_S1, ERG13_S4, and ERG13_S6 strains were sequenced. Sequencing samples were collected when cell growth had resumed, after the initial growth repression. Mutations were found in all the sequences except for the sequences of the URA3_S1 strain (Table 2). The mCherry_S1 and the mTurquoise2 strains had mutations causing frame shifts in the fluorescent protein encoding genes. Strains with gRNAs targeting *PGA3* or *ERG13* had mutations both in the targeted genes and the gRNA sequences (Tables 2 and S3).

**Table 2:** Mutations identified in a selection of strains, where the target genes and the gRNA expression modules were sequenced when cell growth had resumed, after an initial growth repression

Strain	Target gene	Nucleotide mutation	Amino acid mutation	Type of mutation	gRNA mutation
mCherry_S1	*mCherry*	96–100 DelCGAGA, 104_105ins	F32, E33, I34 > F32, E35fs	Frameshift	–
mTurquiose2_S1	*mTurquoise2*	65–70 delTTAATG	V22, D23, G24 > Gfs	Frameshift	–
URA3_S1	*URA3*	–	–	–	–
PGA3_S1	*PGA3*	838–846 delCCTGATGGA	del280P, 281D, 282G	Amino acids deletion	15-16insT in crRNA
ERG13_S4	*ERG13*	A45G, 45_46insG	L16Afs	Frameshift	G30A, G37A, G75A in tRNA and G72T in sgRNA
ERG13_S6	*ERG13*	A581G	D194G	Amino acid substitution	6-7insT, 15-16insC in crRNA

Samples for sequencing were taken after 20 h of cultivation in medium lacking ATc (mCherry_S1, mTurquiose_S1, and URA3_S1, see Fig. 2A), or after 36 h (PGA3_S1 #3 see Figure S4D or 45 h (ERG13_S4 #1 and ERG13_S6 #1, see Figure S4E and F) of cultivation in medium supplemented with 10 µg/mL ATc (Fig. S4).

**Fig. 4:**
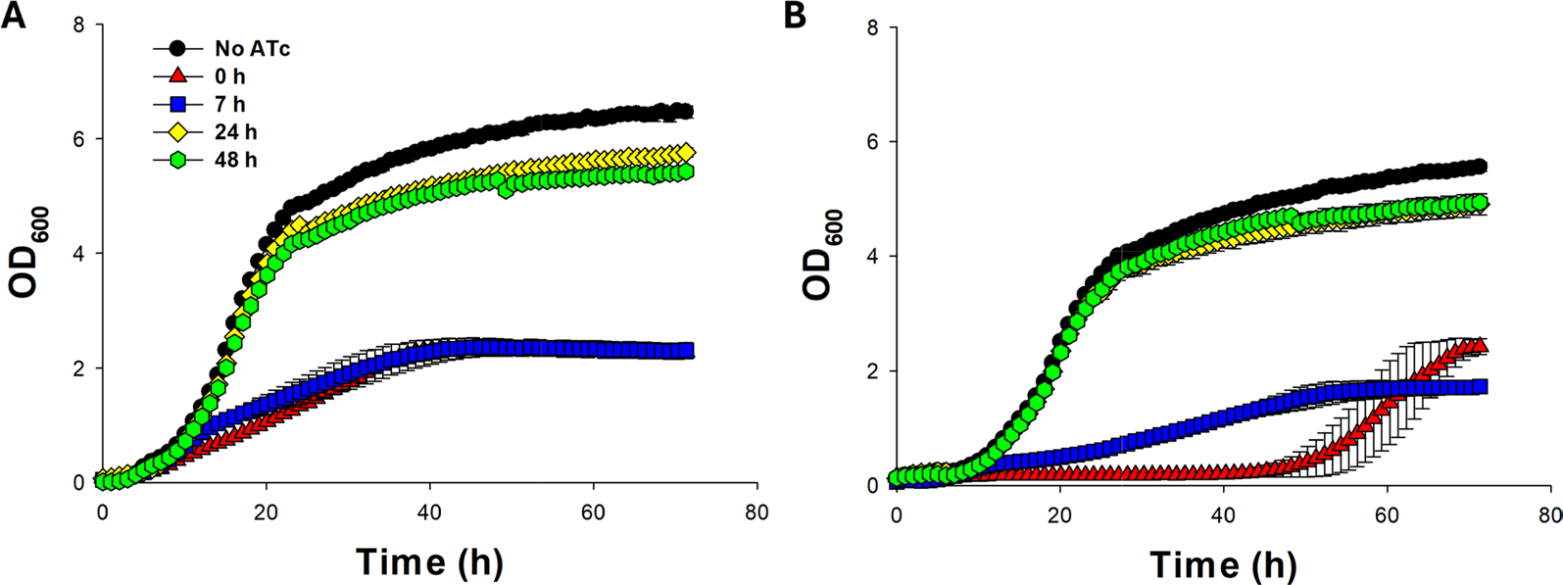
Growth profiles of the control strain (**A**) and ERG13_S6 (**B**) in SD–URA medium supplemented with G418, without ATc (black circle), or with 10 µg/mL ATc added at 0 (red triangle), 7 (blue square), 24 (yellow diamond) or 48 (green hexagon) h after inoculation. Values represent the means of three independent experiments, with error bars indicating the standard error

To investigate the effect of induction timing on the KiSS system inactivation, ATc was added to ERG13_S6 and control cultures after 7, 24 and 48 h (Fig. 4). When ATc was added 7 h after inoculation the generation time was hampered and the maximum OD_600_ value of ERG13_S6 was compromised by approximately 25% compared to the control strain. On the contrary, when ATc was added 24 or 48 h after inoculation, the growth of either strain was barely affected (Fig. 4).

### Co-expression of two gRNAs improves the KiSS system stability

**Fig. 5:**
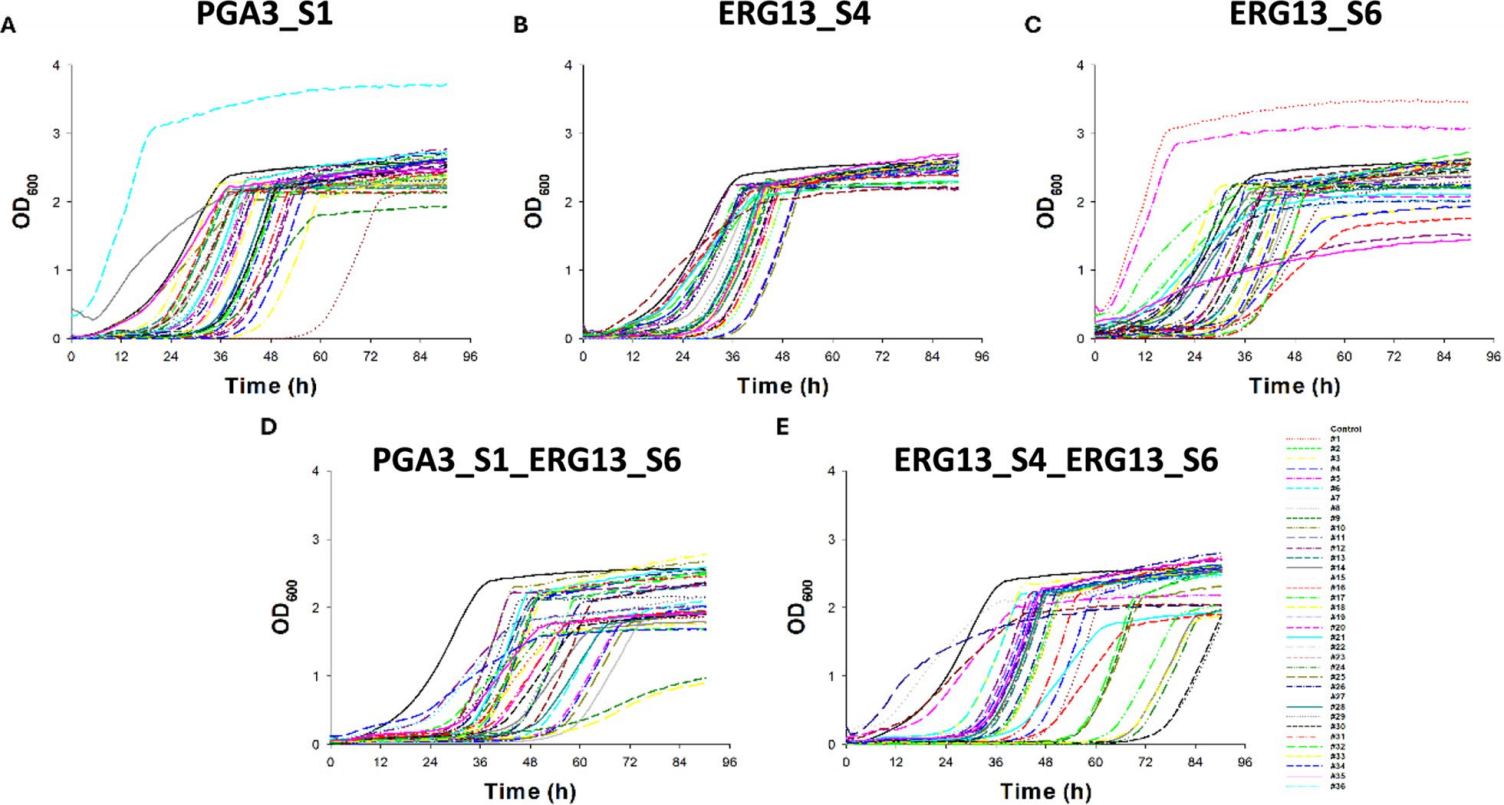
Growth profiles of the control strain and (**A)** PGA3_S1, (**B)** ERG13_S4, (**C)** ERG13_S6, (**D)** PGA3_S1_ERG13_S6, and (**E)** ERG13_S4_ERG13_S6 in SD—URA medium supplemented with G418 and 10 µg/mL of ATc. Individual cultures (n = 36 per strain) indicated by different symbols were cultivated for 90 h.

**Fig. 6:**
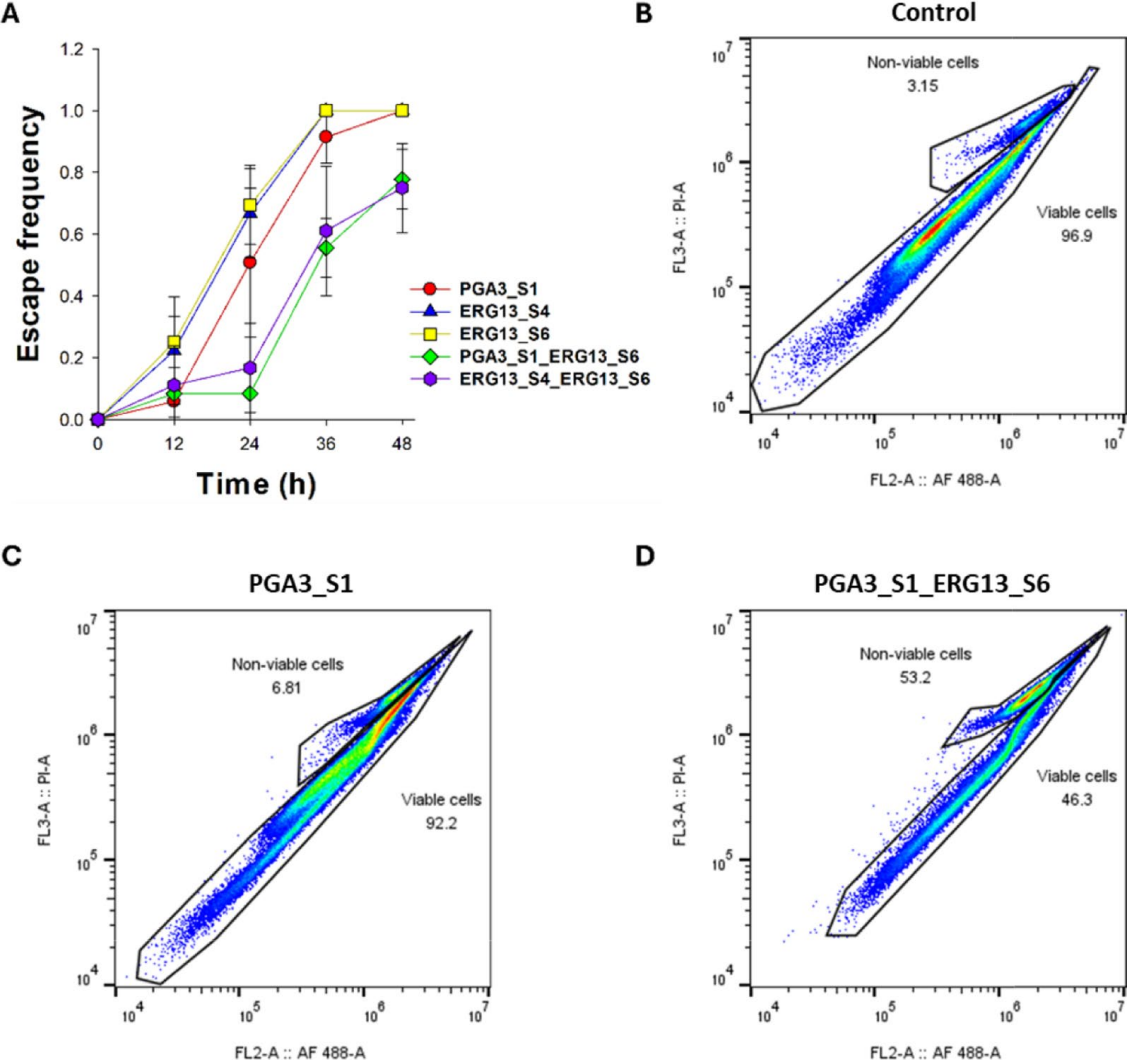
Escape frequency, i.e. proportion of cultures (n = 36) that had resumed growth in KiSS strains after a specified time (**A**) and flow cytograms of the control strain (**B**), PGA3_S1 (**C**), and PGA3_S1_ERG13_S6 (**D)** as determined by Funga*Light* straining of cells collected after 24 (**B**), 48 (C) or 54 h (**D**) of cultivation, when growth was resumed and the cells were at mid-exponential phase. (Fig. 5). Individual cultures of PGA3_S1 (red circle;), ERG13_S4 (blue triangle;), ERG13_S6 (yellow square;), PGA3_S1_ERG13_S6 (green diamond;), and ERG13_S4_ERG13_S6 (purple hexagon;) were cultivated in SD–URA medium supplemented with G418 and 10 µg/mL of ATc. Values represent the means of three independent experiments, with error bars indicating the standard error

With the hypothesis that multiple gRNAs may increase the cutting efficiency and thus the effect of the KiSS system, two gRNA expression modules (targeting *ERG13* and/or *PGA3*) were combined on one plasmid. Individual cultures (n = 36) of both single (PGA3_S1, ERG13_S4, and ERG13_S6) and dual (PGA3_S1_ERG13_S6, and ERG13_S4_ERG13_S6) gRNA KiSS strains were cultivated at 0 (Figure S5) and 10 µg/mL ATc (Fig. 5). After 72 h, both single gRNA and dual gRNA strains showed recovered growth in medium without (Figure S5) or with ATc (Fig. 5). The effectiveness of the KiSS system was determined by escape frequency. The escape frequency was determined as the proportion of cultures where growth was resumed after the initial growth inhibition—presumably as the strains had evaded the KiSS system. At 24 h, the escape frequency of the induced single gRNA strains ranged from 50 to 70%, whereas approximately 90% of the dual gRNA strains remained inviable (escape frequency: 0.1; Fig. 6A). All the induced ERG13_S4 and ERG13_S6 strains resumed growth within 36 h (escape frequency 1), and merely 9% of the PGA3_S1 strains remained inviable at this time point (escape frequency 0.91). By 48 h, all the single gRNA strains and approximately 75% of the dual gRNA had recovered growth. In medium lacking ATc, all strains resumed growth faster compared to when the KiSS system was induced (Figure S6).

To further examine the inactivation of the KiSS system, the cell viability of the control strain, PGA3_S1 and PGA3_S1_ERG13_S6 were monitored through flow cytometry (Fig. 6B–D). The samples were taken when cultures had resumed growth, in the mid-exponential growth phase (Fig. 5). The PGA3_S1_ERG13_S6 strain still showed decreased viability with 53% of cells remaining non-viable (Fig. 6D). In contrast, merely 3% of the control cells and 7% of the PGA3_S1 cells were non-viable at this time point (Fig. 6B, C).

## Discussion

In the KiSS system, Cas9 was constitutively expressed, while the gRNA expression was controlled by an ATc-inducible *RPR1* RNA polymerase III (P_RPR1-tetO_) promoter containing a 19 bp tetO site (Fig. 1). This was expected to enable precise regulation of gRNA expression, allowing controlled activation of the KiSS system. Addition of ATc at the cultivation start (Figs. 2C, E and 4B) or at early exponential phase (at 7 h, Fig. 4B) induced the KiSS system, leading to inhibited growth of strains with gRNAs targeting the essential genes *PGA3*, *ERG13*, *CDC19* or *TPI1* compared to uninduced conditions (Figs. 2A and S3). In contrast, the KiSS strains with gRNAs targeting *URA3* showed no growth defect (Fig. 2A and S2, in total 5 gRNAs tested), likely due to the presence of two *URA3* copies—the native copy and an additional cloning marker. A single functional *URA3* gene can support cell growth, which may allow the cells’ own repair mechanisms to fix any cut made to the second *URA3* copy. Adding ATc after 24 h—when the cells were approaching stationary phase—or at 48 h, during stationary phase, did not alter the growth of ERG13_S6 compared to uninduced conditions (Fig. 4). We ration that this may be attributed to a reduction in the number of active cells and decreased demand for ergosterol, as a previous report showed that ergosterol levels decreased during the stationary phase [26]. To extend the KiSS system applicability during the stationary phase, targeting genes essential for cell maintenance during the stationary growth phase could be effective. Indeed, targeting *CDC19* and *TPI1* proved to be very efficient, leading to more severely hampered growth compared to when *ERG13* or *PGA3* was targeted (Fig. 2).

The KiSS strains targeting the essential genes *PGA3, ERG13, CDC19* and *TPI1* (Figure S3) exhibited moderate growth inhibition even without ATc supplementation, suggesting leaky expression from the inducible P_RPR1-tetO_ promoter. This leakiness may result from the basal transcription of gRNA in the absence of induction. However, previous studies using the same *RPR1* promoter with a single tetO for CRISPR interference reported no growth defects without ATc [20, 27]. In an inducible CRISPR-Cas9 system for mammalian cells the U6 promoter with a single tetO site led to unintended gene editing due to leaky gRNA expression without ATc induction, which was minimized by using two tetO sites [28]. On the contrary, Rottinghaus et al. [11] reported that P_tet_ promoters with two tetO sites showed leakiness in a dual-gRNA CRISPR-Cas9 based kill switch in *E. coli*. These findings highlight the need for optimizing the P_RPR1-tetO_ promoter in our KiSS system to minimize leaky expression. Approaches like modifying the promoter expressing the tetO gene to include multiple operator sites [29] or co-expression of the reverse tetracycline transactivator, rtTA [30] have proven efficient for improving the reliability of the tet system.

In this study, we aimed to design a CRISPR-based kill switch. Tightly controlled induction of the KiSS system was however not achieved, as the KiSS strains were capable of escaping the system. Notably, there was a great variability among KiSS strain replicates, where some strains grew as well as the control strain despite ATc induction (Figs. 2E, 5 and S4). We hypothesized that this may be due to mutations and therefore sequenced crucial components of the KiSS system. Indeed, we identified mutations in gRNA sequences and the target gene, likely compromising the system’s functionality (Table 2). Even a single nucleotide mismatch between the genomic target site and the gRNA has been shown to significantly reduce or even deactivate Cas9 nuclease activity, especially when the mismatch is near the PAM site [31].

To improve the KiSS system, we developed KiSS strains that simultaneously expressed two gRNAs targeting essential genes (*PGA3* and *ERG13*). The hypothesis was that this may kill cells more efficiently as there could be two cuts in the genome occurring simultaneously. Multiplex gene editing approaches have been shown to enhance gene knockout efficiency in *S. cerevisiae* [32]. Increasing the number of conditionally expressed essential genes has been shown to increase the reliability of biocontainment [33]. An optimized dual gRNA CRISPR-based kill switch in *E. coli* was shown to improve killing efficiency and reduced escape frequencies to below detection limit after 1.5 h of induction [11]. In this study, we applied this approach by either targeting different loci within the same gene (*ERG13*) or simultaneously targeting two different genes (*ERG13* and *PGA3*). As the efficiency of CRISPR systems is known to be highly dependent on the gRNA sequence [34], different gRNAs targeting *ERG13, CDC19* and *TPI1* (Figs. 2C and S3) were tested. Among these, ERG13_S4 and ERG13_S6, PGA3_S1*,* CDC19_S1*,* and TPI1_S1 led to significant growth inhibition (Fig. 2C, E), demonstrating that gRNA choice critically impacts the function of the circuit. Compared to single gRNA KiSS strains, the dual gRNA system indeed led to improved efficiency in upon ATc addition (Fig. 5). During the first 24 h, less than 10% of the cells managed to escape the dual KiSS system (Fig. 6). Still, after a much-prolonged long lag phase (36 h), cell growth still resumed also in the dual-gRNA strains (Figs. 5 and 6).

The inactivation of biocontainment systems, often attributed to genetic mutations, remains a major challenge [13]. Kill switches based on induced lethality are known to be prone to spontaneous mutations within the lethality inducing genes or through a gained immunity against suicide gene products, causing system failure [35]. Prior studies have demonstrated that targeting essential genes provides a more mutation-resistant alternative, as mutations in essential genes typically are less feasible [5]. Evolved protection mechanisms, such as DNA repair and positioning of essential genes in cold spots for mutation, have been reported to reduce the mutation rate in essential genes [36, 37] Yet, essential gene-based regulatory circuits in bacteria and yeast have shown system inactivation caused by mutations in regulatory components such as Cas9, inducible promoters, gRNAs, repressor-binding domains within the regulatory switches, recombinase enzymes, and other genes involved in the system regulation [6, 7, 11, 38]. In line with these findings, the sequencing of escaped KiSS mutants revealed mutations in the gRNA and target genes (Table 2).

The lack of standardized characterization of escape events hampers the comparison of different kill switch systems and limits their translation to large-scale applications [13]. Terms such as escape rate, escape frequency, reversion frequency, and fraction viable are used to describe the proportion of the cells circumventing a kill switch. While escape frequencies have been quantified for few cell population control strategies in *S. cerevisiae*, CRISPR-based kill switches have so far been characterized primarily in *E. coli* (Table 3). In this study, the proportion of culture circumventing the kill switch was reported as the escape frequency. Upon ATc induction, single gRNA KiSS strains (PGA3_S1 and ERG12_S4/S6) fully recovered after 36–48 h (Fig. 6A). In contrast, dual gRNAs KiSS strains (PGA3_S1_ERG13_S6 and ERG13_S4_ERG13_S6) demonstrated lower escape frequencies, with approximately 25% of the cells remaining non-growing even after 48 h (Fig. 6A). The PGA3_S1_ERG13_S6 strain showed a dramatic increase in the amount of non-viable cells (53%) also after cell growth had resumed, demonstrating that escaping the dual gRNA KiSS system was indeed much more difficult for the cells (Fig. 6D). The optimization of the inducing system and chosen genes to target simultaneously may lead to more stringent population control, as demonstrated in other kill switch systems (Table 3). To further improve the system, the combination of the Cas9-based kill switch with another system such as a conditionally expressed nuclease, recombinase or anti-CRISPR protein or could be attempted.

**Table 3:** Comparison of selected kill switch strategies and reported escape rates of the systems

Organism	Strategy	Mechanism	Escape rate*	Calculation method	References
*S. cerevisiae*	Auxotrophy	Adenine auxotrophy (*ade2-1* mutation)	Not reported	Not applicable	[39]
	Conditional essentiality	Inducible expression of essential histone genes (*HHTS* and *HHFS*) and of expression of a site-specific recombinase	Less than < 10^–10^	Escape rate calculated using method of median [40]. Reversion frequency = (colony forming units (CFUs) on restrictive plates) / (CFUs on permissive plates)	[6]
		Regulation of essential genes (*FAS2*, *RPB11 HTS1*, *SEC4*, and *SEC17*) using inducible promoters	Less than < 10^–7^	Escape rate calculated using method of median [40]. Reversion frequency = (colony forming units (CFUs) on restrictive plates) / (CFUs on permissive plates)	[38]
	Toxin expression	Regulation of toxin gene (*relE*) using an inducible promoter	Not reported	Not applicable	[41]
		Regulation of toxin gene (*nucA*) using an inducible promoter	Not reported	Not applicable	[2]
	CRISPR-based	Inducible gRNA expression, gRNAs targeting essential genes; *ERG13, PGA3, TPI1, CDC19*	~ 0.1	Escape frequency = (number of replicate cultures showing growth recovery under non-permissive conditions)/ (Total number of cultures tested in non-permissive condition)	This study
*E.* *coli*	CRISPR-based	2-input inducible *cas9* expression, gRNAs targeting essential genes	Less than < 10^–8^	Fraction viable = (CFUs in the non-permissive condition)/ (CFUs in the permissive condition)	[11]
		Inducible CRISPR system (CasABCDE + Cas3), gRNAs targeting streptomycin- resistance genes on a plasmid	Not reported	Not applicable	[9]
*P. putida*		Tet-induced *cas9* expression, gRNAs targeting repetitive genomic elements. Regulated expression of the AcrIIA4 anti-CRISPR protein and TetR	Not reported	Not applicable	[10]

^*^Escape rate refers to the proportion of cells escaping the kill-switch control, reported as escape rate, escape frequency, reversion frequency, or fraction viable

While challenges such as leaky expression and system escape remain, our kill switch provides a valuable foundation for advancing cell growth inhibition circuits in yeast. Unlike earlier kill switches relying on conditional expression of lethality causing genes, CRISPR-based systems have been suggested not to cause horizontal gene transfer in an open environment [13, 42]. We envision that the KiSS system could be used for population control, e.g. in co-cultures, where production is shared between two or more organisms. Recently, our group showed that lactic acid production from lignocellulosic hydrolysates could be improved by co-culturing an inhibitor detoxifying bacterium, *Acinetobacter baylyii* with a lactic acid producing *S. cerevisiae* [43]. In this process, control of the two cell populations was needed to maximize production. Using the KiSS system to hamper the growth of one of the microorganisms once it has completed its intended function for a specific time could be advantageous. Wild type *E. coli* strains were shown to competitively eliminate escaped kill switch strains [11], thus for co-cultivation applications our kill switch may already work well enough.

## Conclusions

In this study, we laid a foundation for a CRISPR-Cas9 based kill switch in *S. cerevisiae*, CRISPR KiSS as a synthetic genetic device to regulate cell proliferation by leveraging inducible targeting of essential genes. Our results showed that both gRNA selection, induction timing, and dual gRNA system are key parameters influencing the KiSS system performance. The dual gRNA KiSS system improved efficiency and reduced escape frequencies, although prolonged cultivation eventually led to system escape. Although the KiSS system requires further optimization to enhance both efficiency and reliability of the system, the flexibility and tunability of the KiSS system provide a promising avenue for future cell factory applications.

## Supplementary Information

Supplementary material 1: Figure S1. Fluorescence microscopy images of selected strains. Figure S2. Growth profiles and the generation time at the maximum specific growth rate plotted against the lag time of KiSS strains with single gRNA targets at different ATc concentrations. Figure S3. Growth profiles of KiSS strains with single gRNA targets without induction. Figure S4. Growth profiles of experimental replicates of KiSS strains with single gRNA targets with and without induction. Figure S5. Growth profiles of experimental replicates of KiSS strains with single or dual gRNA targets without ATc. Figure S6. Escape frequency of cultures without induction. Table S1. List of oligos used. Table S2. Plasmids used and developed. Table S3. Sequencing results of selected KiSS strains.

## Data Availability

All data generated or analyzed during this study are included in this published article and its additional file.

## Abbreviations

ATc: Anhydrotetracycline
CFU: Cell forming unit
DSB: Double stranded break
gRNA: Guide RNA
tetO: Tetracycline operator
tetR: Tetracycline repressor

## References

[1] Stirling F, Bitzan L, O’Keefe S, Redfield E, Oliver JWK, Way J, et al. Rational design of evolutionarily stable microbial kill switches. Mol Cell. 2017;68(4):686–97. 29149596 10.1016/j.molcel.2017.10.033 PMC5812007

[2] Balan A, Schenberg ACG. A conditional suicide system for *Saccharomyces cerevisiae* relying on the intracellular production of the *Serratia marcescens* nuclease. Yeast. 2005;22(3):203–12. 15704225 10.1002/yea.1203

[3] Westmoreland JW, Summers JA, Holland CL, Resnick MA, Lewis LK. Blunt-ended DNA double-strand breaks induced by endonucleases PvuII and EcoRV are poor substrates for repair in *Saccharomyces cerevisiae*. DNA Repair. 2010;9(6):617–26. 20356803 10.1016/j.dnarep.2010.02.008 PMC2883614

[4] Kristoffersen P, Jensen GB, Gerdes K, Piš AJ, Pišˇkur P. Bacterial toxin-antitoxin gene system as containment control in yeast cells. Appl Environ Microbiol. 2000;66(12):5524–6. 11097943 10.1128/aem.66.12.5524-5526.2000 PMC92497

[5] Hoffmann SA, Diggans J, Densmore D, Dai J, Knight T, Leproust E, et al. Safety by design: biosafety and biosecurity in the age of synthetic genomics. iScience. 2023. 10.1016/j.isci.2023.106165. 36895643 10.1016/j.isci.2023.106165 PMC9988571

[6] Cai Y, Agmon N, Choi WJ, Ubide A, Stracquadanio G, Caravelli K, et al. Intrinsic biocontainment: multiplex genome safeguards combine transcriptional and recombinational control of essential yeast genes. Proc Natl Acad Sci U S A. 2015;112(6):1803–8. 25624482 10.1073/pnas.1424704112 PMC4330768

[7] Hoffmann SA, Cai Y. Engineering stringent genetic biocontainment of yeast with a protein stability switch. Nat Commun. 2024;15(1):1060. 38316765 10.1038/s41467-024-44988-8 PMC10844650

[8] Kakko N, Rantasalo A, Koponen T, Vidgren V, Kannisto M, Maiorova N, et al. Inducible synthetic growth regulation using the ClpXP proteasome enhances cis,cis-muconic acid and glycolic acid yields in *Saccharomyces cerevisiae*. ACS Synth Biol. 2023;12(4):1021–33. 36976676 10.1021/acssynbio.2c00467 PMC10127448

[9] Caliando BJ, Voigt CA. Targeted DNA degradation using a CRISPR device stably carried in the host genome. Nat Commun. 2015;6(1):6989. 25988366 10.1038/ncomms7989 PMC4479009

[10] Asin-Garcia E, Martin-Pascual M, de Buck C, Allewijn M, Müller A, Martins dos Santos VAP. GenoMine: a CRISPR-Cas9-based kill switch for biocontainment of *Pseudomonas putida.* Front Bioeng Biotechnol. 2024;12. 10.3389/fbioe.2024.1426107 PMC11439788 39351062

[11] Rottinghaus AG, Ferreiro A, Fishbein SRS, Dantas G, Moon TS. Genetically stable CRISPR-based kill switches for engineered microbes. Nat Commun. 2022;13(1):672. 35115506 10.1038/s41467-022-28163-5 PMC8813983

[12] Kim D, Lee JW. Genetic biocontainment systems for the safe use of engineered microorganisms. Biotech Bioprocess Eng. 2020;25(6):974–84.

[13] Zhu X, Zhang Z, Jia B, Yuan Y. Current advances of biocontainment strategy in synthetic biology. Chin J Chem Eng. 2023;56:141–51.

[14] Jordá T, Puig S. Regulation of ergosterol biosynthesis in *Saccharomyces cerevisiae*. Genes. 2020;11(7):1–18. 10.3390/genes11070795 PMC7397035 32679672

[15] Yu L, Peñ Castillo L, Mnaimneh S, Hughes TR, Brown GW. A survey of essential gene function in the yeast cell division cycle Yu, Lisa, et al. Mol Biol Cell. 2006;17(11):4736–47. 16943325 10.1091/mbc.E06-04-0368 PMC1635385

[16] Pearce AK, Crimmins † Kay, Groussac E, Hewlins MJE, Dickinson JR, Francois J, et al. Pyruvate kinase (Pyk1) levels influence both the rate and direction of carbon flux in yeast under fermentative conditions. Microbiology. 2001;147:391–401. 10.1099/00221287-147-2-391 11158356

[17] Alber T, Kawasaki G. Nucleotide sequence of the triose phosphate isomerase gene of *Saccharomyces cerevisiae*. J Mol Appl Genet. 1982;1(5):419–34. 6759603

[18] Van Dijken JP, Bauer J, Brambilla L, Duboc P, Francois JM, Gancedo C, et al. An interlaboratory comparison of physiological and genetic properties of four *Saccharomyces cerevisiae* strains. Enzyme Microb Technol. 2000;26(9–10):706–14. 10862876 10.1016/s0141-0229(00)00162-9

[19] Lee ME, DeLoache WC, Cervantes B, Dueber JE. A highly characterized yeast toolkit for modular, multipart assembly. ACS Synth Biol. 2015;4(9):975–86. 25871405 10.1021/sb500366v

[20] Smith JD, Suresh S, Schlecht U, Wu M, Wagih O, Peltz G, et al. Quantitative CRISPR interference screens in yeast identify chemical-genetic interactions and new rules for guide RNA design. Genome Biol. 2016;17(1):45. 26956608 10.1186/s13059-016-0900-9 PMC4784398

[21] Mormino M, Siewers V, Nygård Y. Development of an Haa1-based biosensor for acetic acid sensing in *Saccharomyces cerevisiae*. FEMS Yeast Res. 2021. 10.1093/femsyr/foab049. 34477863 10.1093/femsyr/foab049 PMC8435060

[22] Liu H, Wei Z, Dominguez A, Li Y, Wang X, Qi LS. CRISPR-ERA: a comprehensive design tool for CRISPR-mediated gene editing, repression and activation. Bioinformatics. 2015;31(22):3676–8. 26209430 10.1093/bioinformatics/btv423 PMC4757951

[23] Labun K, Montague TG, Krause M, Torres Cleuren YN, Tjeldnes H, Valen E. CHOPCHOP v3: expanding the CRISPR web toolbox beyond genome editing. Nucleic Acids Res. 2019;47(W1):W171–4. 31106371 10.1093/nar/gkz365 PMC6602426

[24] Daniel Gietz R, Woods RA. Yeast transformation by the LiAc/SS Carrier DNA/PEG Method. In: Methods in molecular biology. 2006. p. 107–20. 10.1385/1-59259-958-3:107 16118429

[25] Torello Pianale L, Olsson L. ScEnSor kit for *Saccharomyces cerevisiae* engineering and biosensor-driven investigation of the intracellular environment. ACS Synth Biol. 2023;12(8):2493–7. 37552581 10.1021/acssynbio.3c00124 PMC10443032

[26] Charcosset JY, Chauvet E. Effect of culture conditions on ergosterol as an indicator of biomass in the aquatic hyphomycetes. Appl Environ Microbiol. 2001;67(5):2051–5. 11319080 10.1128/AEM.67.5.2051-2055.2001 PMC92835

[27] Mukherjee V, Lind U, St Onge RP, Blomberg A, Nygård Y. A CRISPR interference screen of essential genes reveals that proteasome regulation dictates acetic acid tolerance in *Saccharomyces cerevisiae*. mSystems. 2021. 10.1128/msystems.00418-21. 34313457 10.1128/mSystems.00418-21 PMC8407339

[28] Sun N, Petiwala S, Wang R, Lu C, Hu M, Ghosh S, et al. Development of drug-inducible CRISPR-Cas9 systems for large-scale functional screening. BMC Genomics. 2019;20(1):225. 30890156 10.1186/s12864-019-5601-9 PMC6425629

[29] Tominaga M, Shima Y, Nozaki K, Ito Y, Someda M, Shoya Y, et al. Designing strong inducible synthetic promoters in yeasts. Nat Commun. 2024;15(1):10653. 39702268 10.1038/s41467-024-54865-z PMC11659477

[30] Roney IJ, Rudner AD, Couture JF, Kærn M. Improvement of the reverse tetracycline transactivator by single amino acid substitutions that reduce leaky target gene expression to undetectable levels. Sci Rep. 2016;6(1):27697. 27323850 10.1038/srep27697 PMC4914848

[31] Hsu PD, Scott DA, Weinstein JA, Ran FA, Konermann S, Agarwala V, et al. DNA targeting specificity of RNA-guided Cas9 nucleases. Nat Biotechnol. 2013;31(9):827–32. 23873081 10.1038/nbt.2647 PMC3969858

[32] McCarty NS, Graham AE, Studená L, Ledesma-Amaro R. Multiplexed CRISPR technologies for gene editing and transcriptional regulation. Nat Commun. 2020;11(1):1281. 32152313 10.1038/s41467-020-15053-x PMC7062760

[33] Pavão G, Sfalcin I, Bonatto D. Biocontainment techniques and applications for yeast biotechnology. Fermentation. 2023;9(4):341.

[34] Jung WJ, Park SJ, Cha S, Kim K. Factors affecting the cleavage efficiency of the CRISPR-Cas9 system. Anim Cells Syst. 2024;28(1):75–83. 10.1080/19768354.2024.2322054 PMC10911232 38440123

[35] Moe-Behrens GHG, Davis R, Haynes KA. Preparing synthetic biology for the world. Front Microbiol. 2013;4:5. 23355834 10.3389/fmicb.2013.00005 PMC3554958

[36] Melde RH, Bao K, Sharp NP. Recent insights into the evolution of mutation rates in yeast. Curr Opin Genet Dev. 2022;76:101953. 35834945 10.1016/j.gde.2022.101953 PMC9491374

[37] Zhang Z, Ren Q. Why are essential genes essential? The essentiality of *Saccharomyces* genes. Microb Cell. 2015;2(8):280–7. 28357303 10.15698/mic2015.08.218 PMC5349100

[38] Agmon N, Tang Z, Yang K, Sutter B, Ikushima S, Cai Y, et al. Low escape-rate genome safeguards with minimal molecular perturbation of *Saccharomyces cerevisiae*. Proc Natl Acad Sci U S A. 2017;114(8):E1470–9. 28174266 10.1073/pnas.1621250114 PMC5338387

[39] Kokina A, Kibilds J, Liepins J. Adenine auxotrophy—be aware: some effects of adenine auxotrophy in *Saccharomyces cerevisiae* strain W303-1A. FEMS Yeast Res. 2014;14(5):697–707. 24661329 10.1111/1567-1364.12154

[40] Lea DE, Coulson CA. The distribution of the numbers of mutants in bacterial populations. J Genet. 1949;49(3):264–85. 24536673 10.1007/BF02986080

[41] Duperray M, François JM, Capp JP. Tuning the expression of the bacterial relBE toxin-antitoxin system in *Saccharomyces cerevisiae* allows characterizing the subsequent growth inhibition. FEMS Yeast Res. 2023;23:foad009. 36722160 10.1093/femsyr/foad009

[42] Wheatley RM, MacLean RC. CRISPR-Cas systems restrict horizontal gene transfer in *Pseudomonas aeruginosa*. ISME J. 2021;15(5):1420–33. 33349652 10.1038/s41396-020-00860-3 PMC8105352

[43] Liu C, Choi B, Efimova E, Nygård Y, Santala S. Enhanced upgrading of lignocellulosic substrates by coculture of *Saccharomyces cerevisiae* and *Acinetobacter baylyi* ADP1. Biotechnol Biofuels Bioprod. 2024;17(1):61. 38711153 10.1186/s13068-024-02510-8 PMC11075230

