# Supplementary material for: Towards the development of a CRISPR-Cas9 based kill switch for Saccharomyces cerevisiae
Source: Microb Cell Fact. 2026 Feb 20;25:62. doi: 10.1186/s12934-026-02959-2 (PMC12930942; doi:10.1186/s12934-026-02959-2)
Supplement: Supplementary file 1 — Supplementary material 1: Figure S1. Fluorescence microscopy images of selected strains. Figure S2. Growth profiles and the generation time at the maximum specific growth rate plotted against the lag time of KiSS strains with single gRNA targets at different ATc concentrations. Figure S3. Growth profiles of KiSS strains with single gRNA targets without induction. Figure S4. Growth profiles of experimental replicates of KiSS strains with single gRNA targets with and without induction. Figure S5. Growth profiles of experimental replicates of KiSS strains with single or dual gRNA targets without ATc. Figure S6. Escape frequency of cultures without induction. Table S1. List of oligos used. Table S2. Plasmids used and developed. Table S3. Sequencing results of selected KiSS strains. [file 12934_2026_2959_MOESM1_ESM.docx]

# **Supplementary information**

# **Towards the development of CRISPR-Cas9 based kill switch for *Saccharomyces cerevisiae***

**Pavithra Umashankar^1^** **^†^, Bohyun Choi^1^** **^†^ and Yvonne Nygård^1,2#^**

^1^Department of Life Sciences, Chalmers University of Technology, Gothenburg, Sweden.

^2^VTT Technical Research Centre of Finland, Espoo, Finland.

† These authors contributed equally to this work and share first authorship.

#Corresponding author: yvonne.nygard@chalmers.se


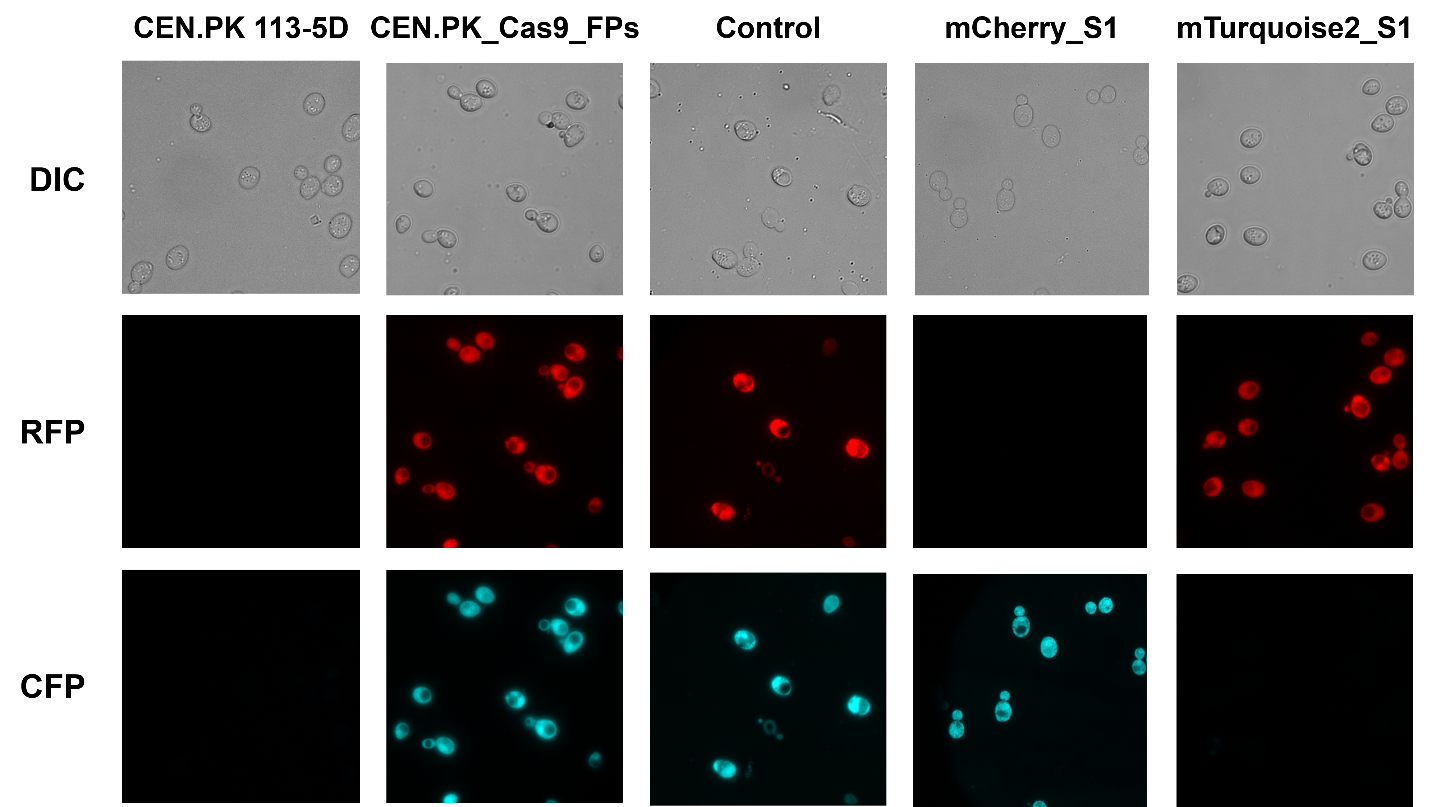


**Figure S1**. Fluorescence microscopy images of selected strains. CEN.PK 113-5D and CEN.PK_Cas9_FPs were cultured in SD-CSM and SD-URA media, respectively. The control strain, mCherry_S1, and mTurquoise2_S1 were cultured in SD-URA medium with G418. All cultures were carried out at 30°C for 48 h, and samples were collected after 20 h.

**A**

**C**

**B**

**D**


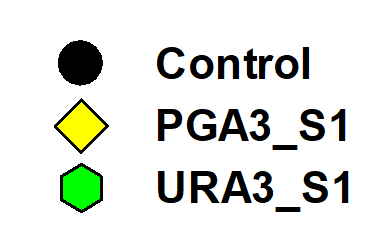

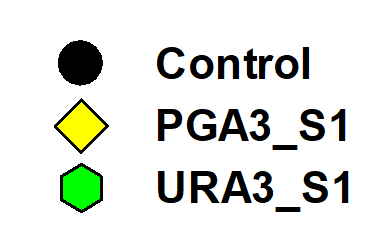

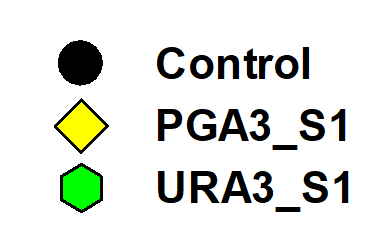


**Figure S2.** Growth profiles (A and C) and the generation time at the maximum specific growth rate plotted against the lag time (B and D) of the PGA3_S1 (yellow diamond;
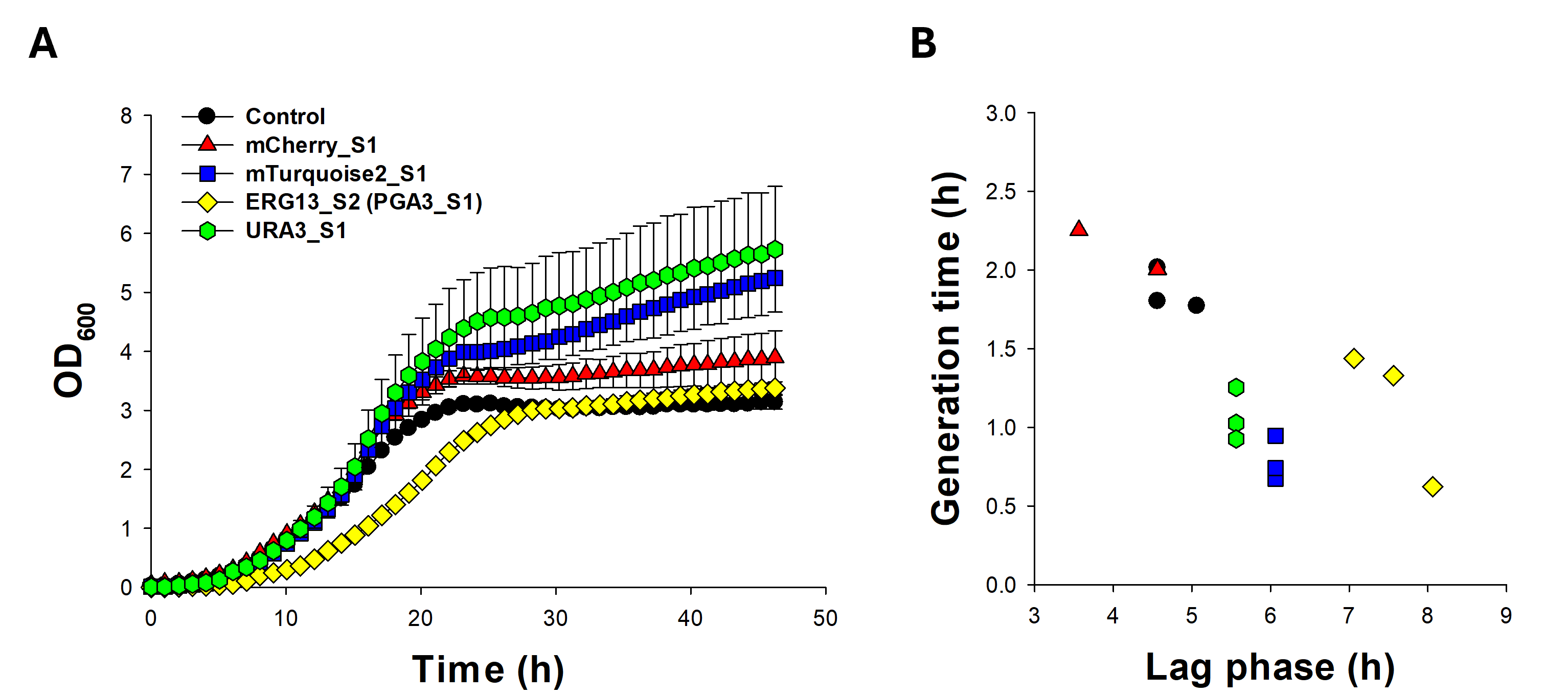
) and URA3_S1 (green hexagon;
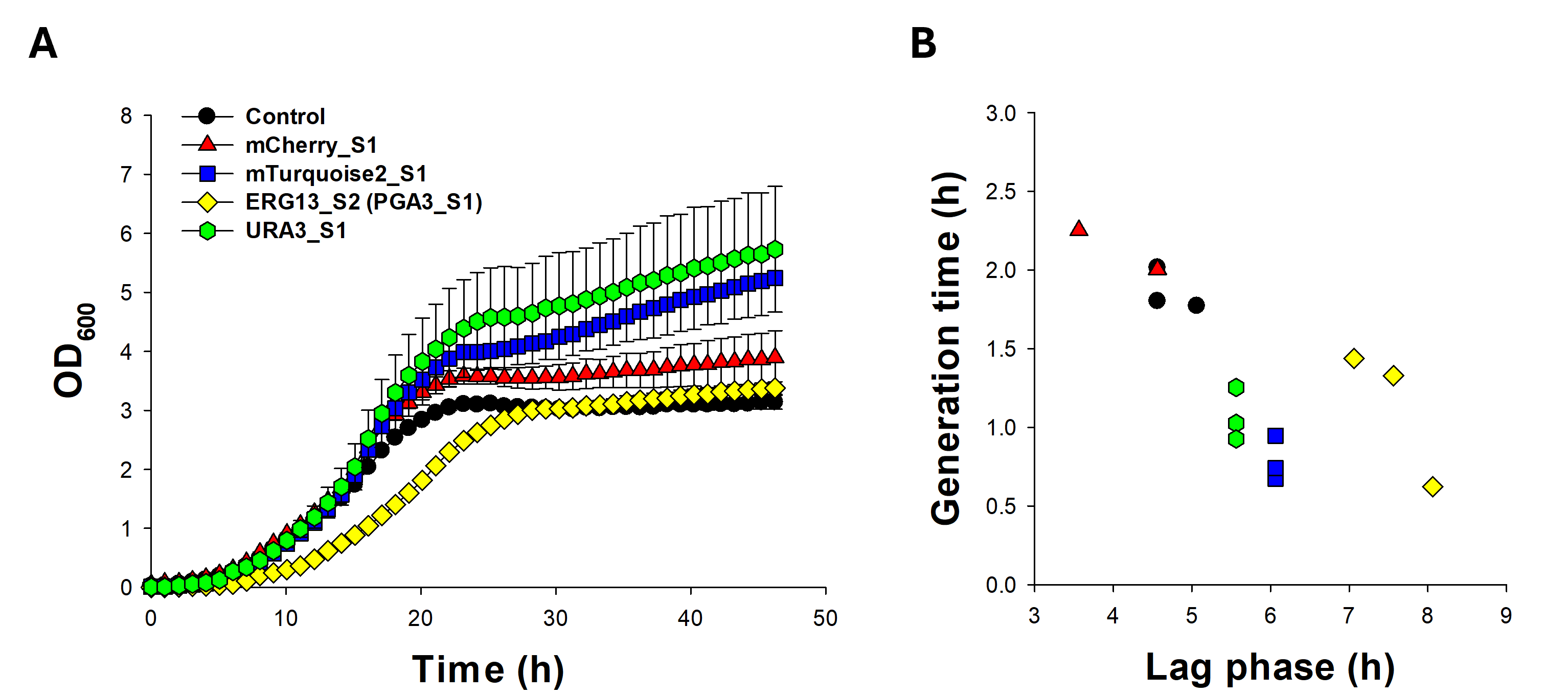
) cultivated in SD – URA medium supplemented with G418 and 5 (A and B) or 10 (C and D) µg/mL ATc. Values represent the means of three independent experiments, with error bars indicating the standard error.

**B**

**A**

**Figure S3**. Growth profiles (A and B) of a representative set of KISS strains: control strain (black circle;
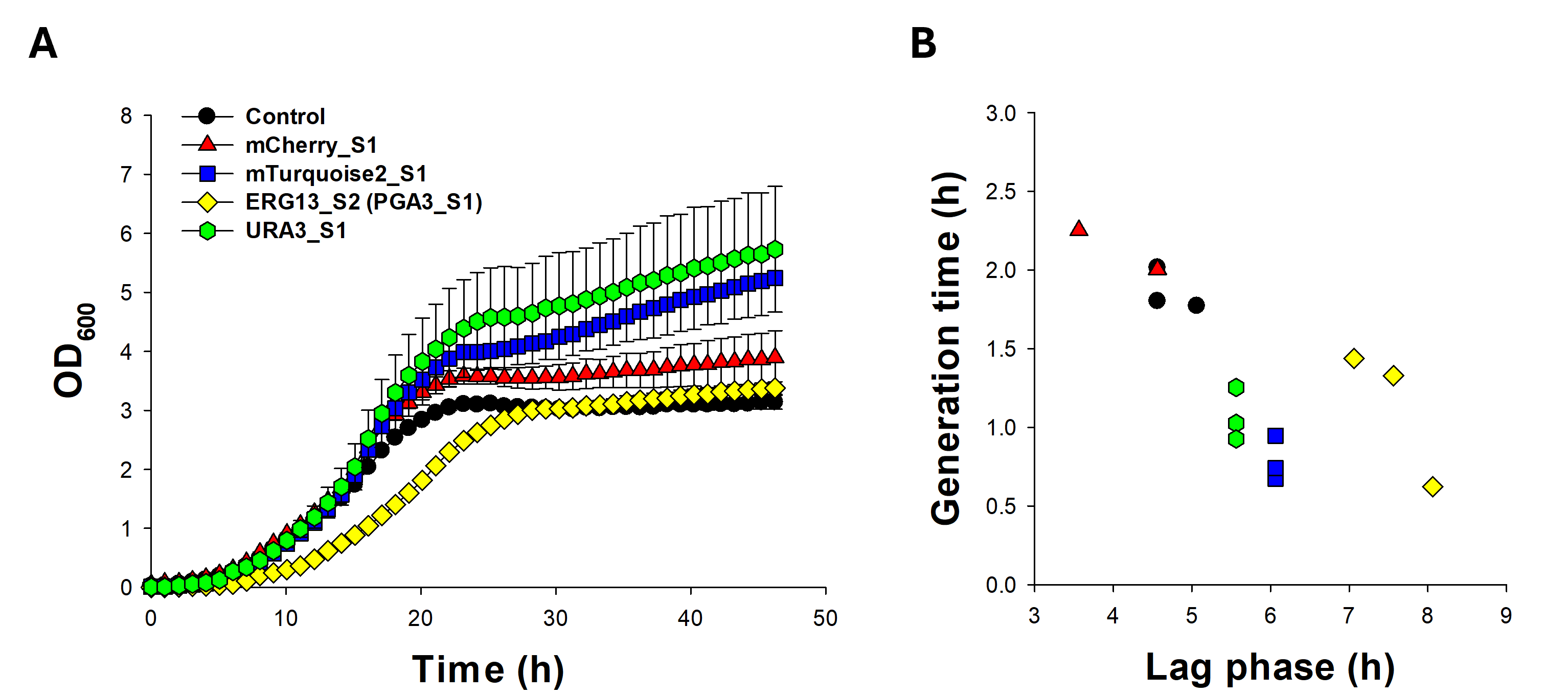
), ERG13_S1 (red triangle;
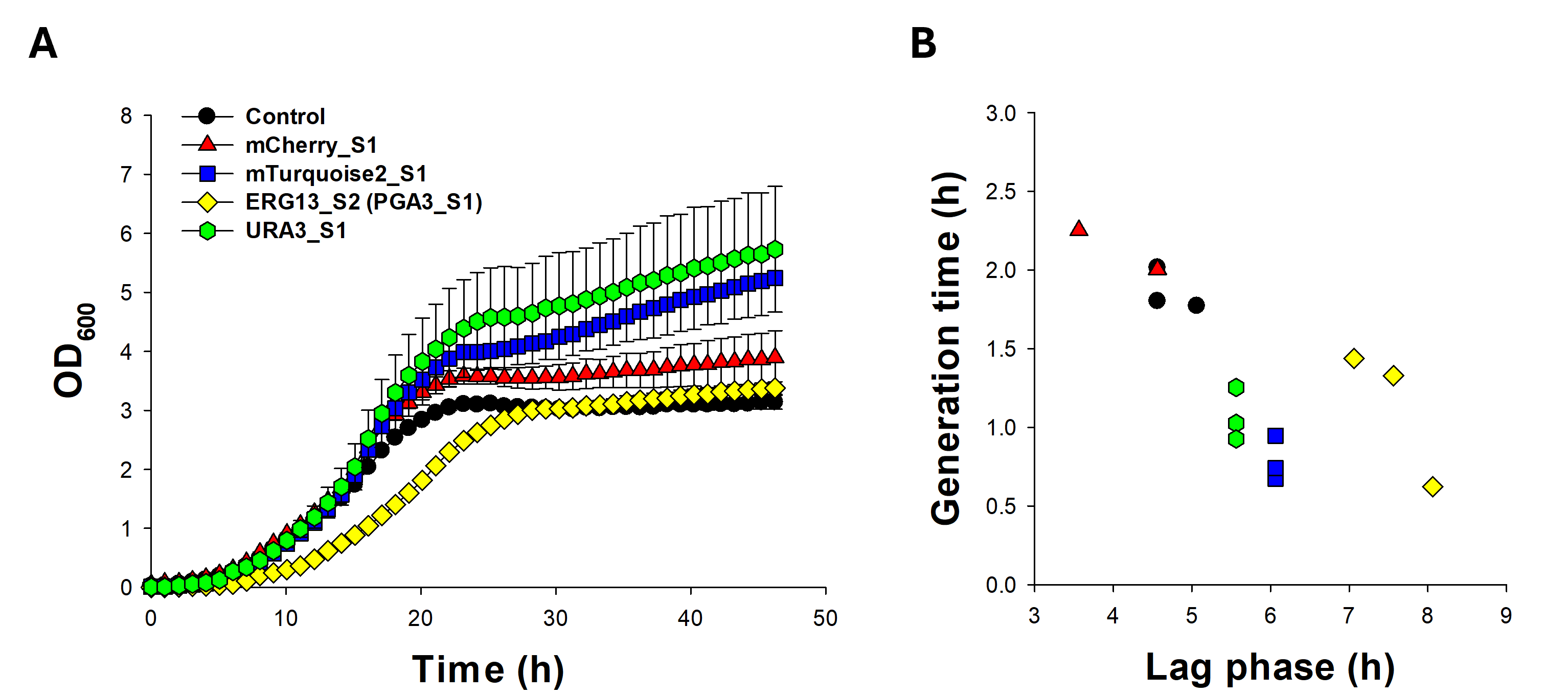
), PGA3_S1 (blue square;
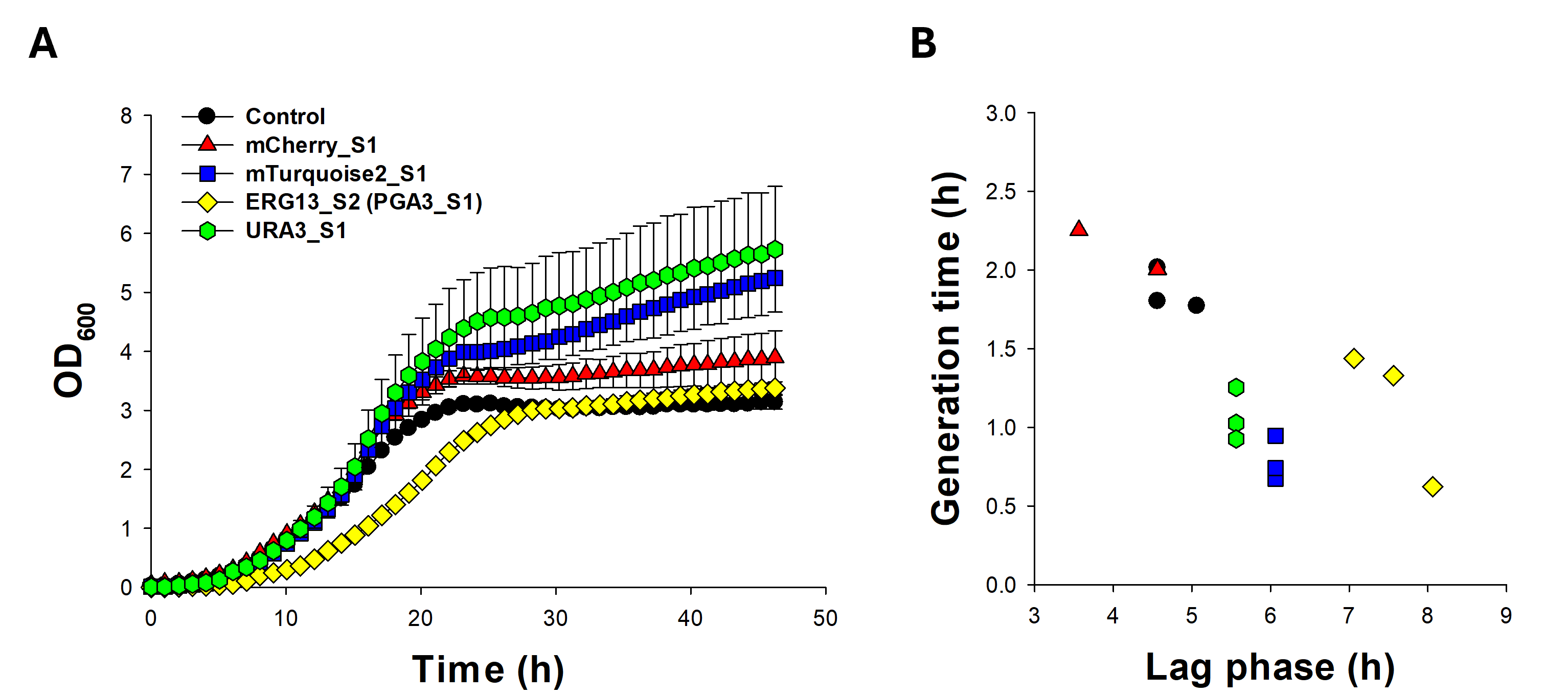
), ERG13_S4 (yellow diamond;
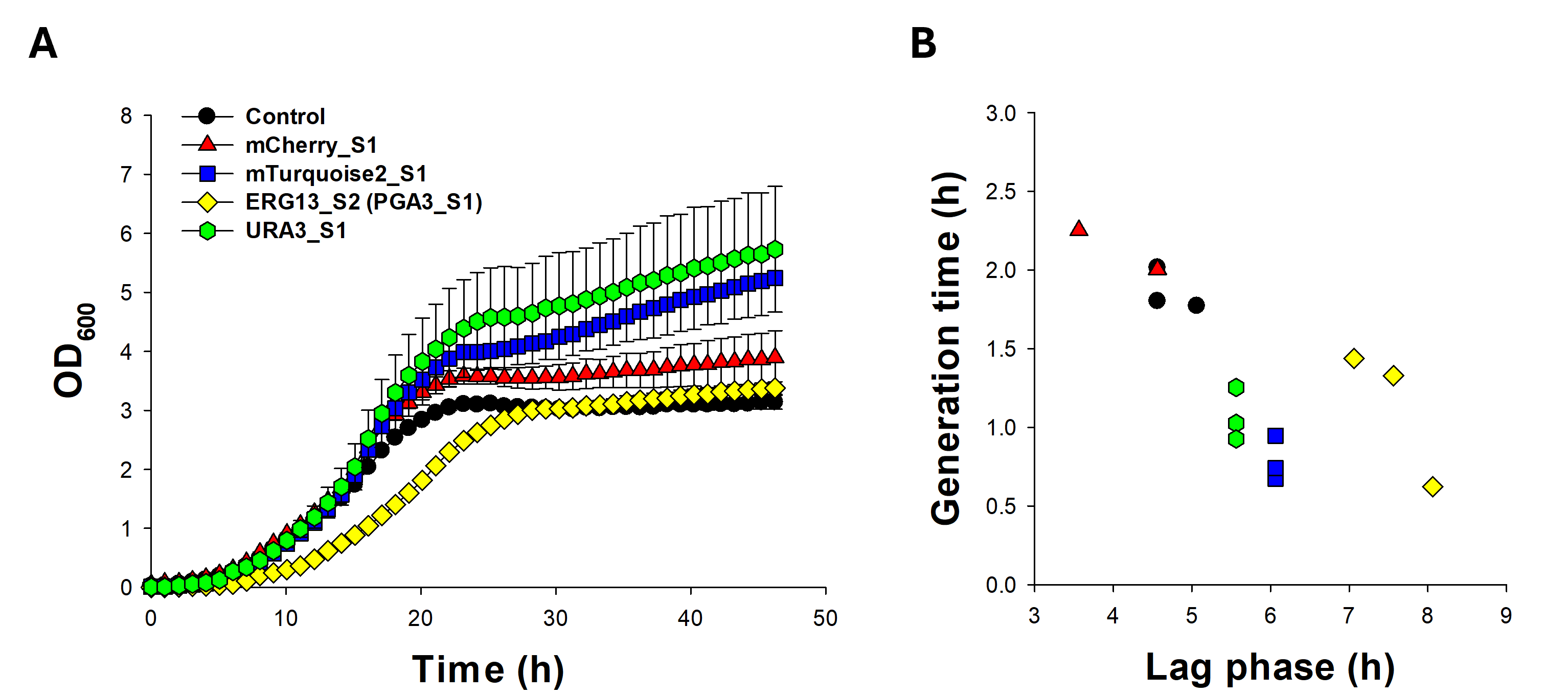
), ERG13_S5 (green hexagon;
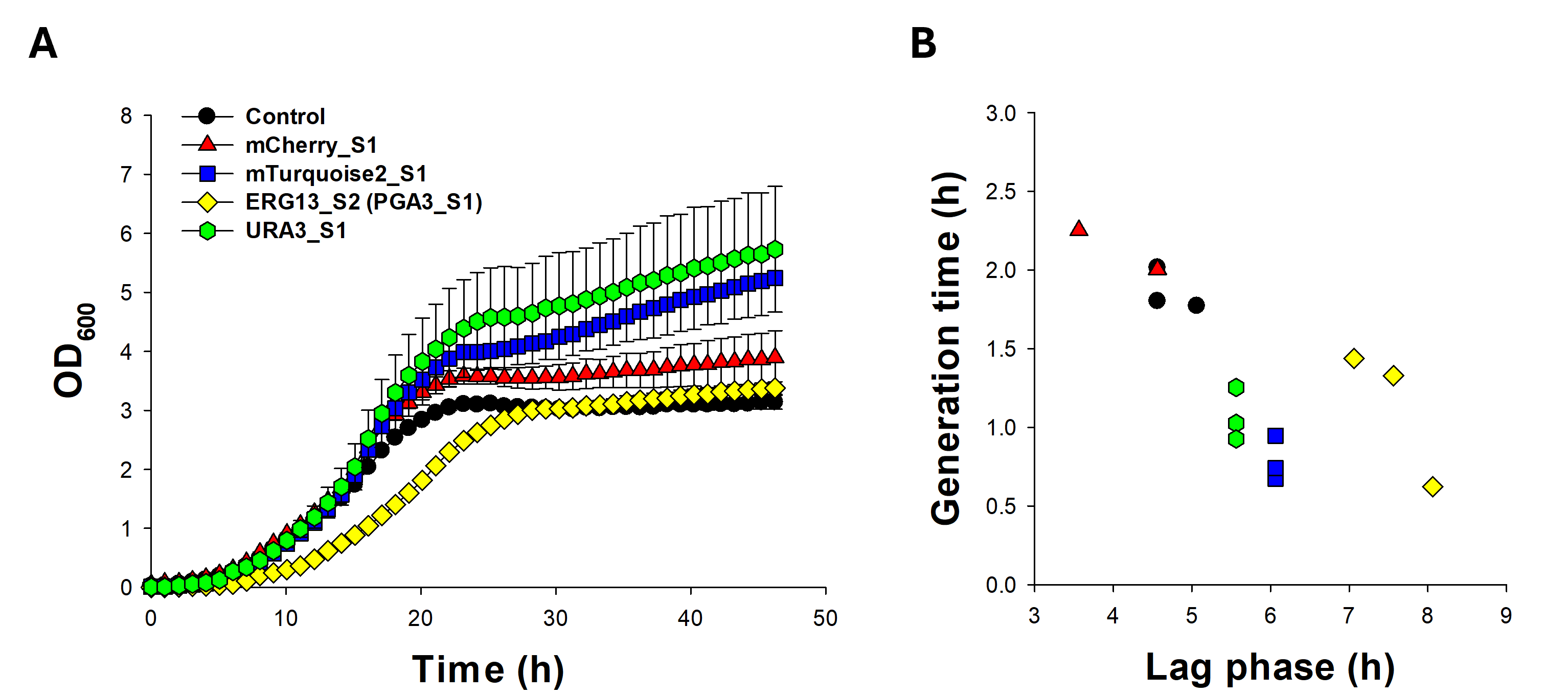
), ERG13_S6 (purple inverted triangle;
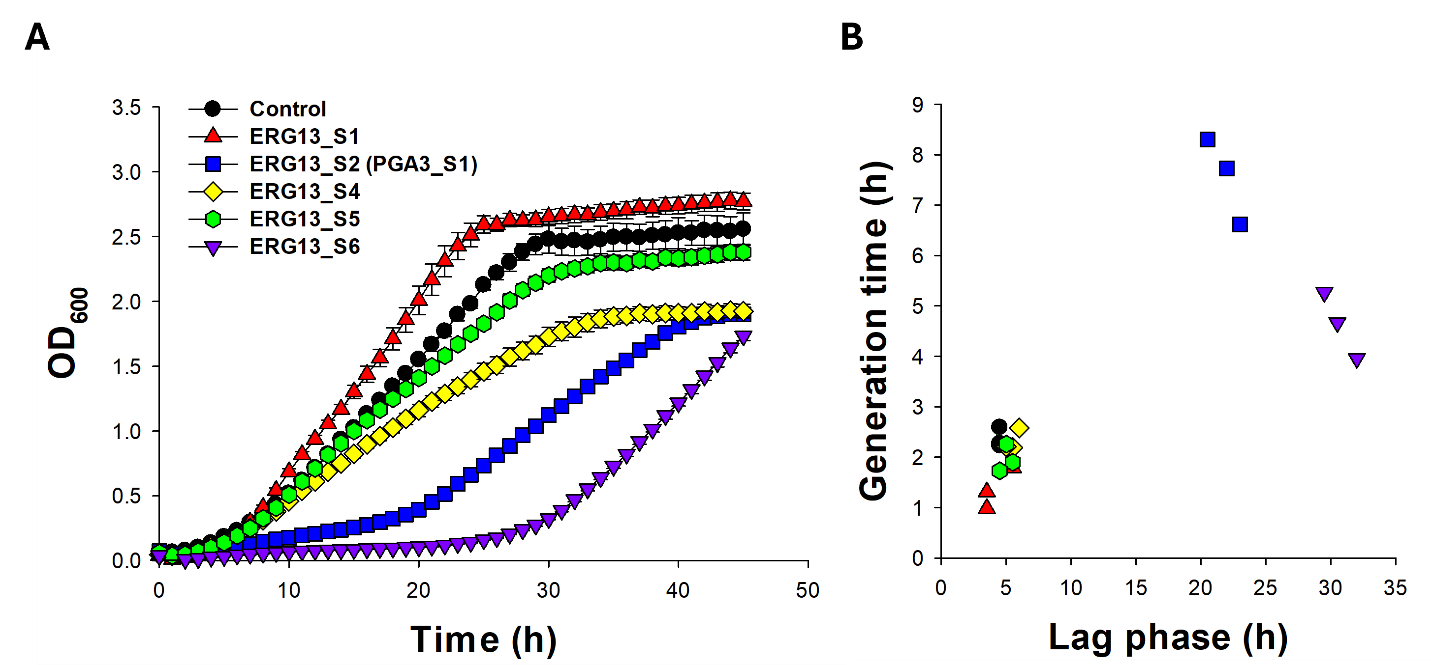
) , TPI1_S1 (light green cross-hair triangle, ), TPI1_S2 (light blue cross-hair square;), CDC19_S1 (light yellow cross-hair diamond;), and CDC19_S2 (light red cross-hair hexagon; ) cultured in SD – URA medium supplemented with G418.


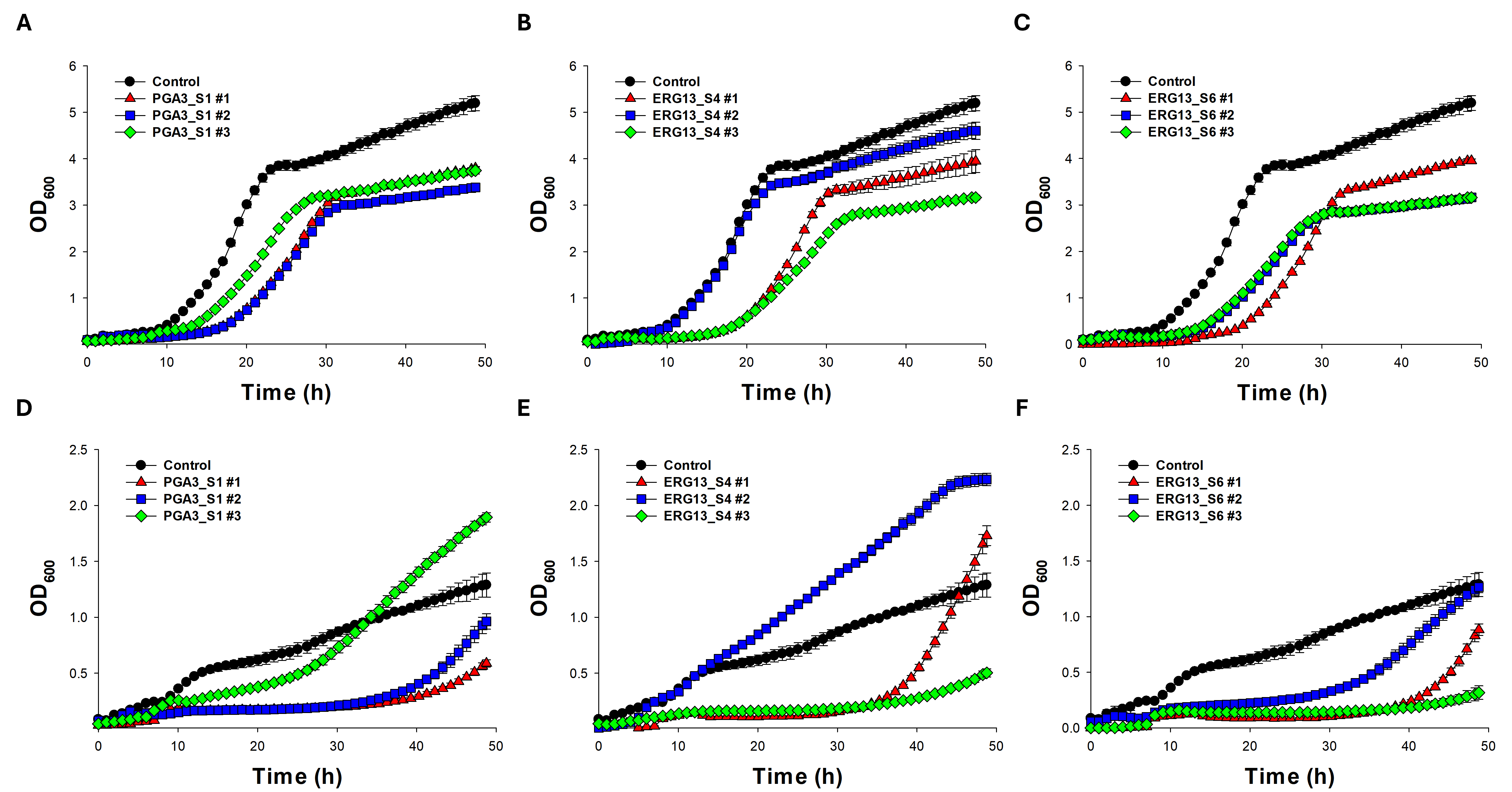


**Figure S4.** Growth profiles of the control strain (black circle;
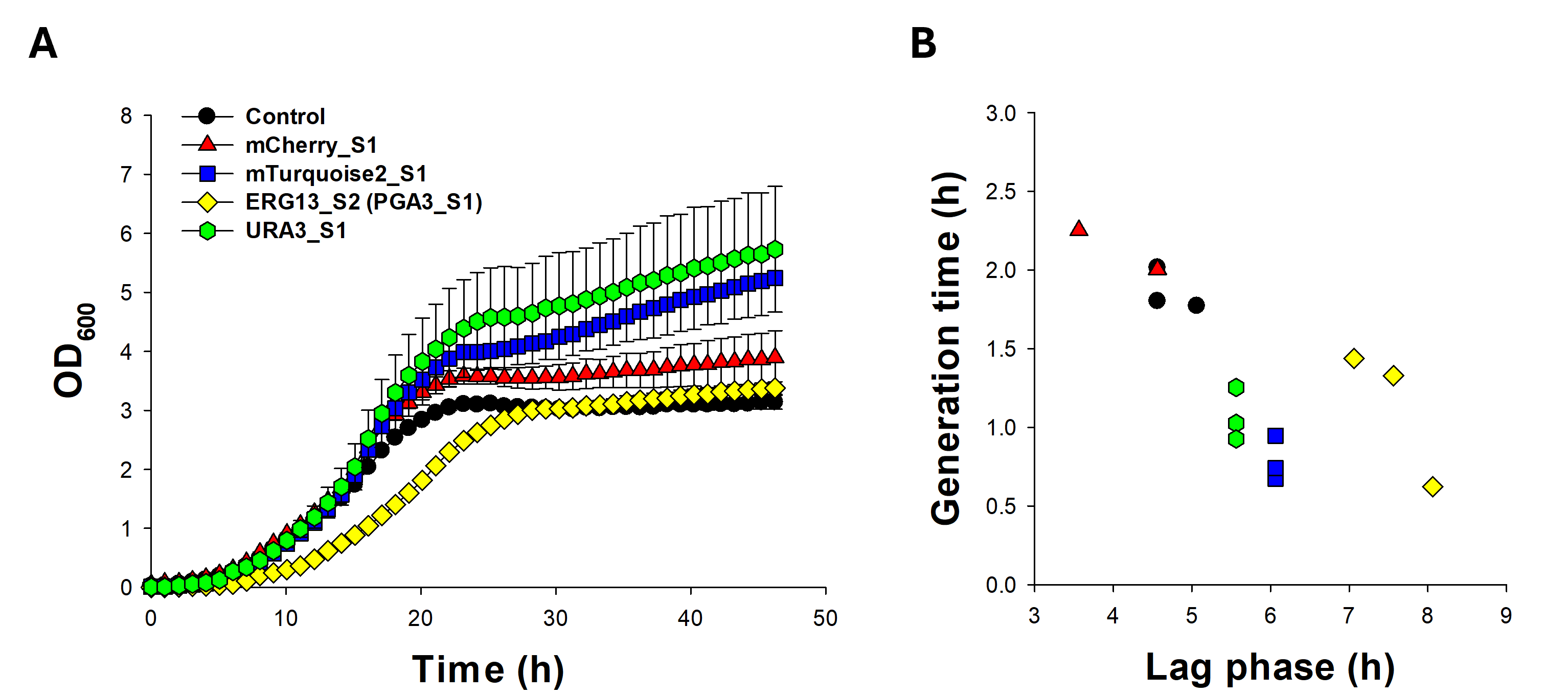
), and cultures (#3) of PGA3_S1 (A and D), ERG13_S4 (B and E), and ERG13_S6 (C and F) grown in SD – URA medium supplemented with G418, at 0 (A, B and C) and 10 (D, E and F) µg/mL of ATc, added after 7 h of cultivation. Independent cultures of the respective KiSS strains are indicated by different symbols: colony #1 (red triangle;
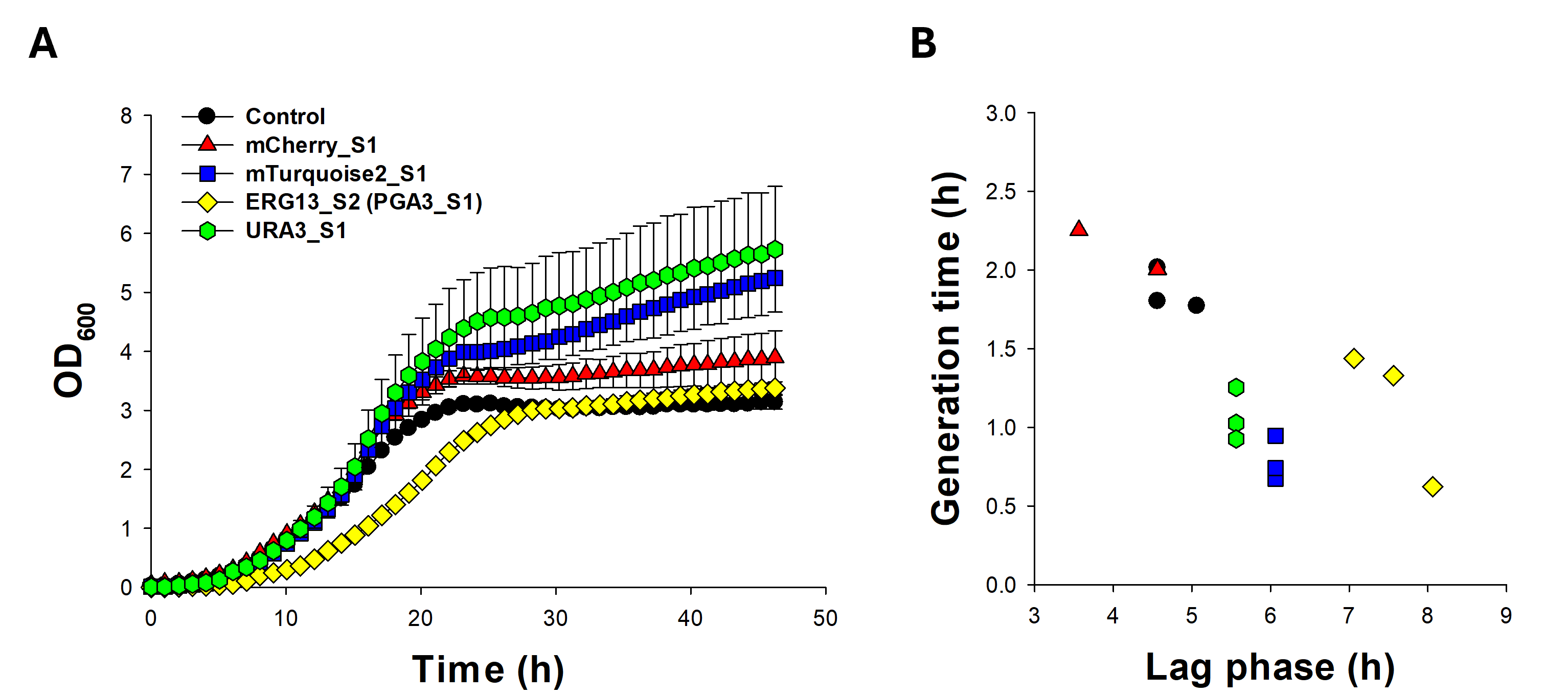
), colony #2 (blue square;
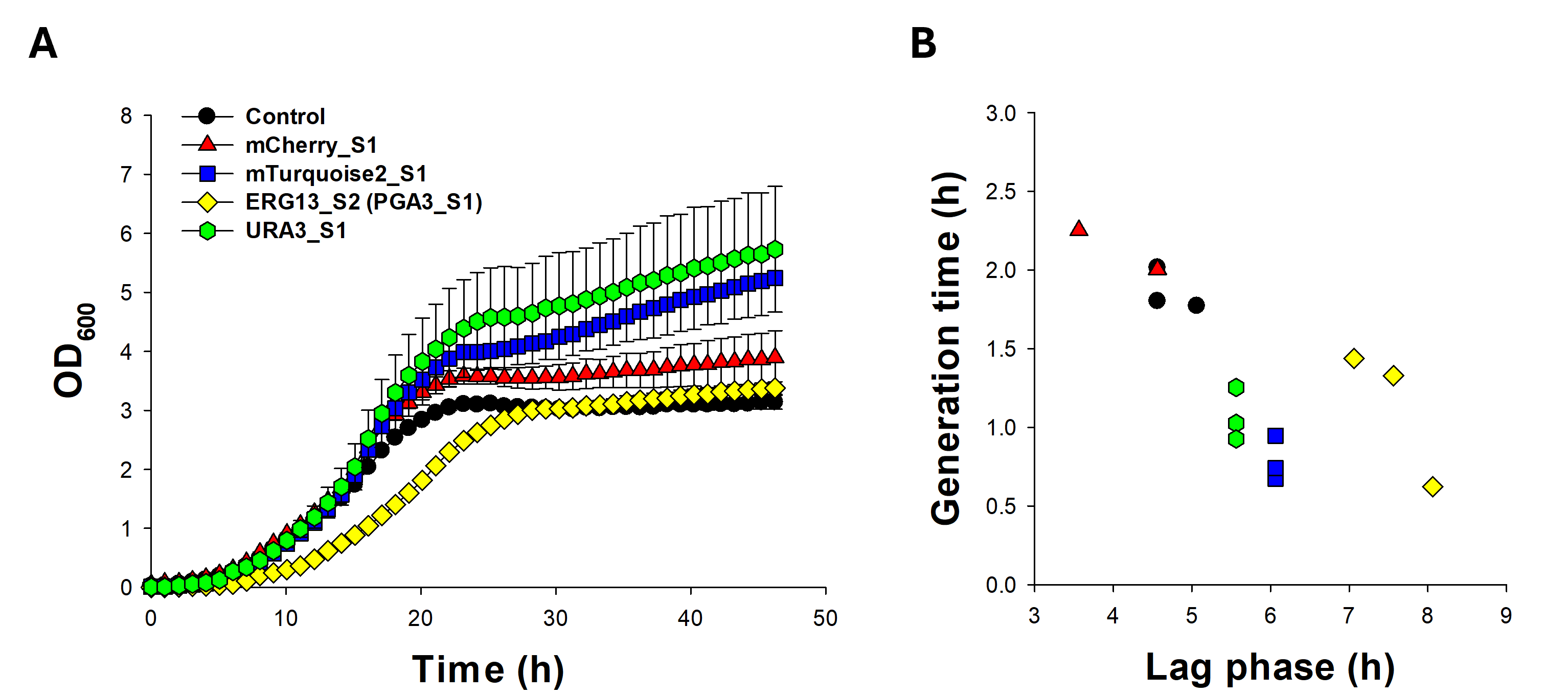
), and colony #3 (green hexagon;
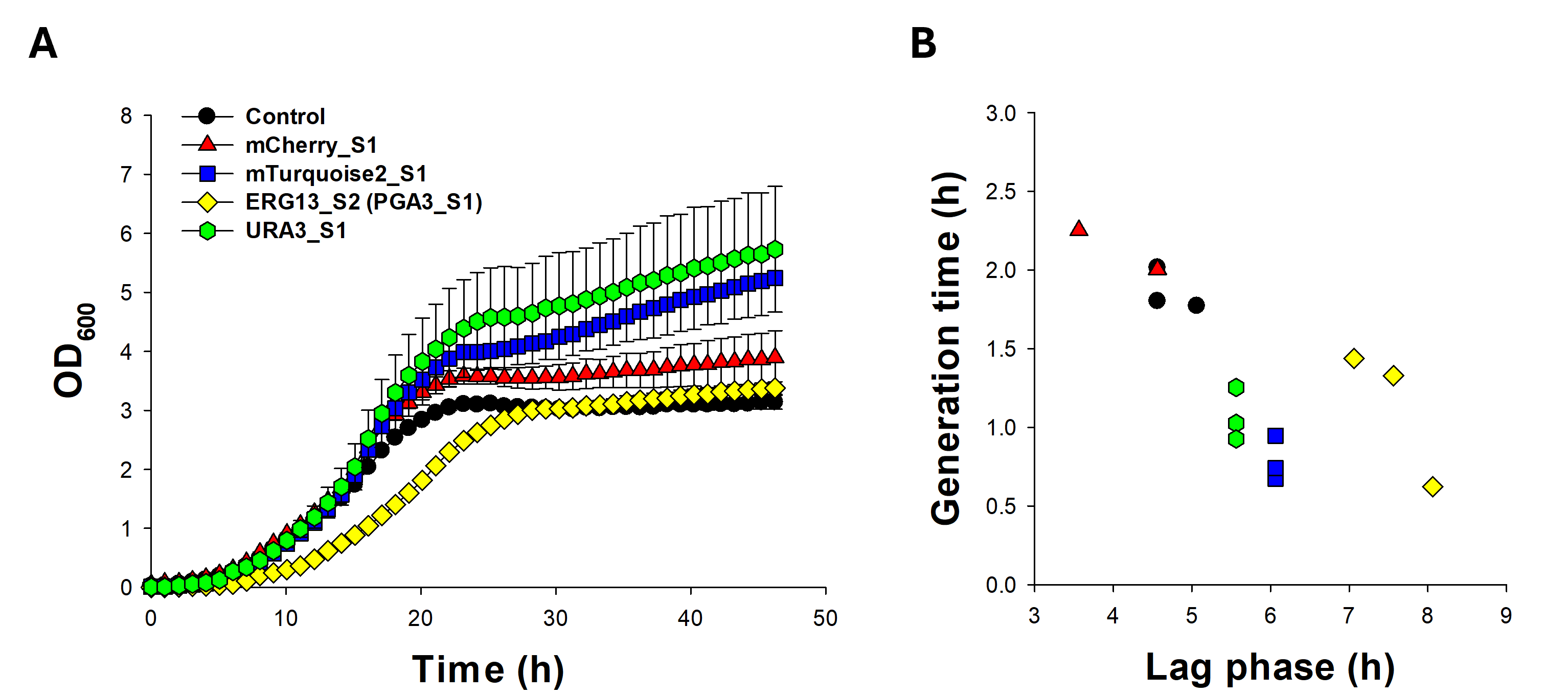
).

**
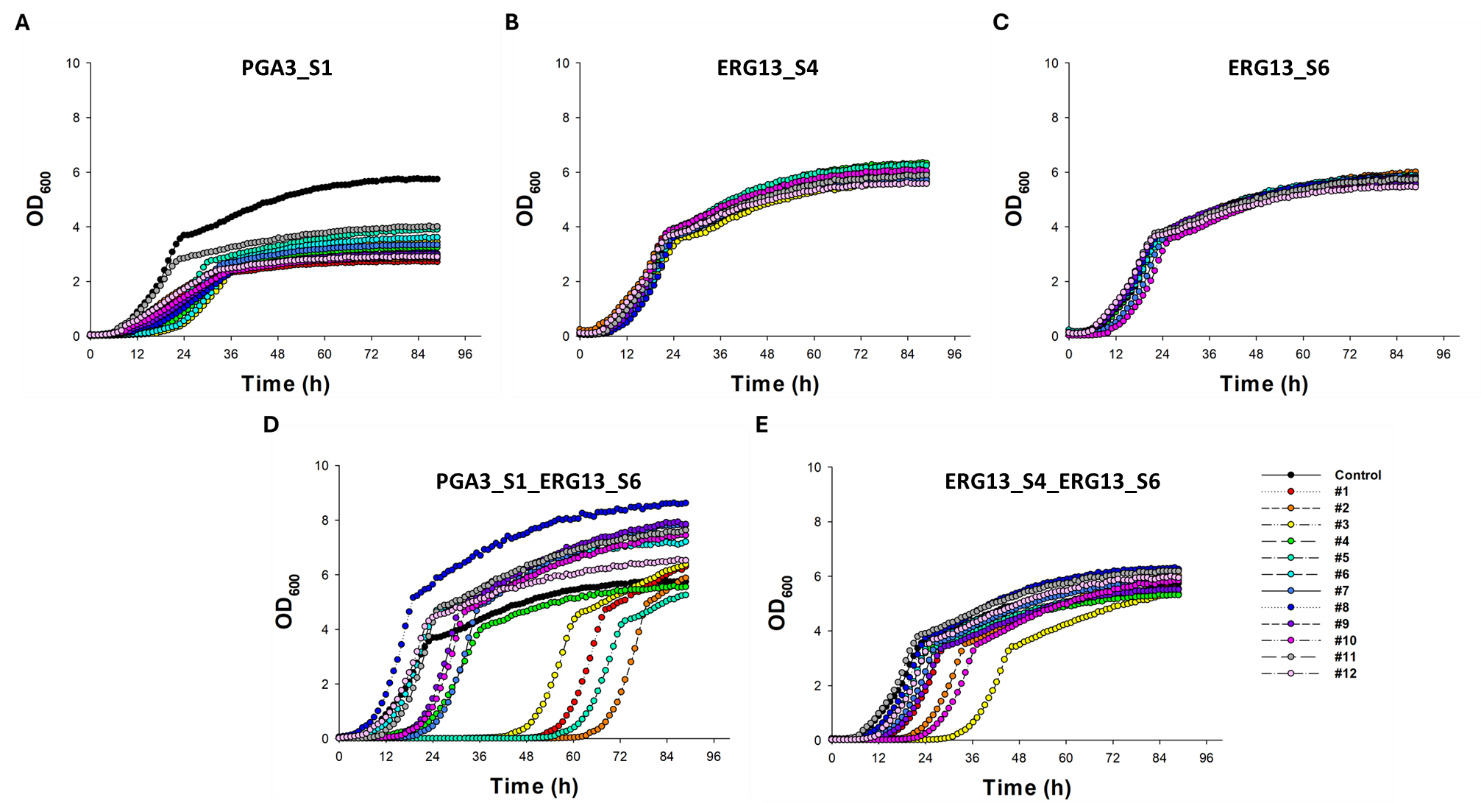
**

**Figure S5.** Growth profiles of the control strain (black circle,
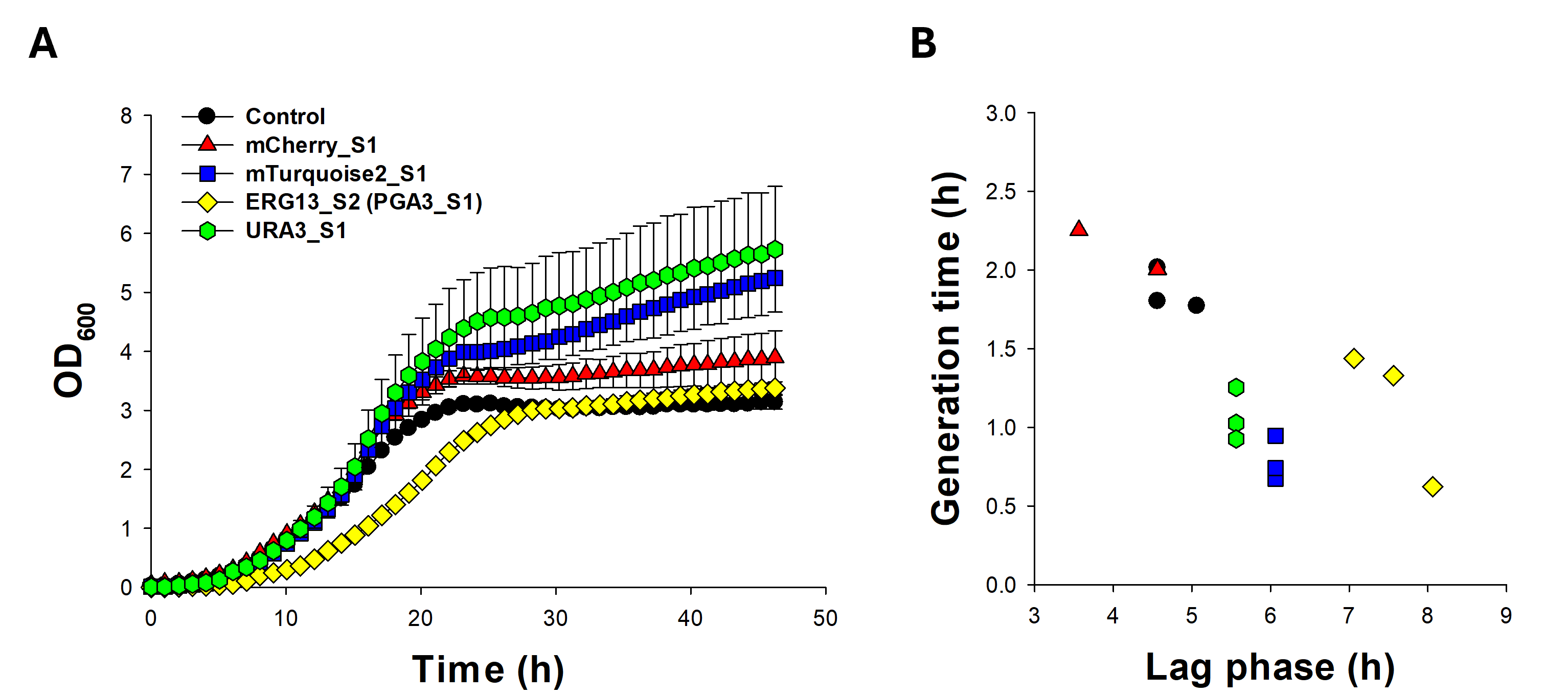
) and (A) PGA3_S1, (B) ERG13_S4, (C) ERG13_S6, (D) PGA3_S1_ERG13_S6, and (E) ERG13_S4_ERG13_S6 in SD – URA medium supplemented with G418 and 0 µg/mL of ATc. Individual colonies (n = 12 per strain) were cultivated for 90 h. Symbols indicate the colony evaluated: colony #1 (red circle,
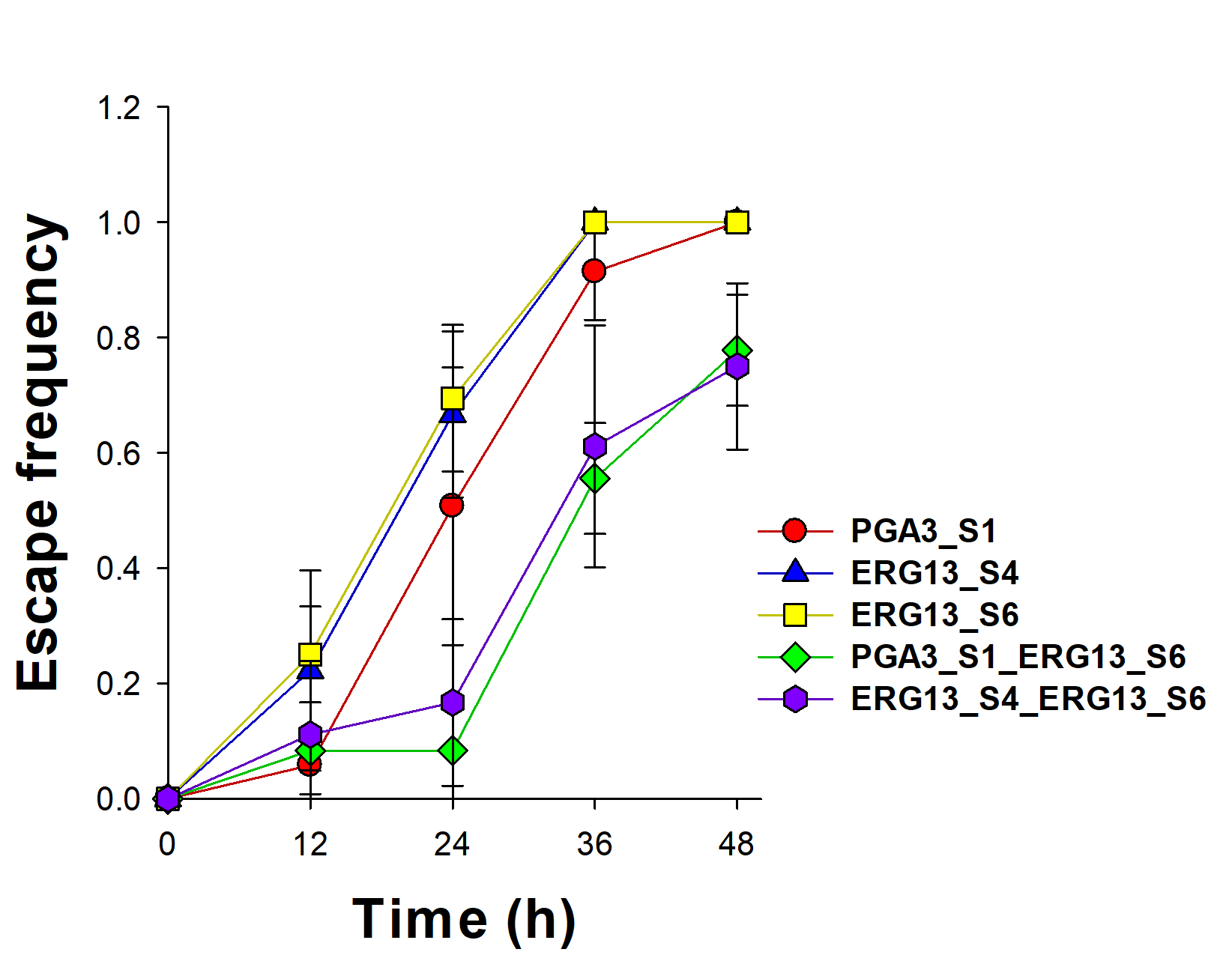
), colony #2 (orange circle,
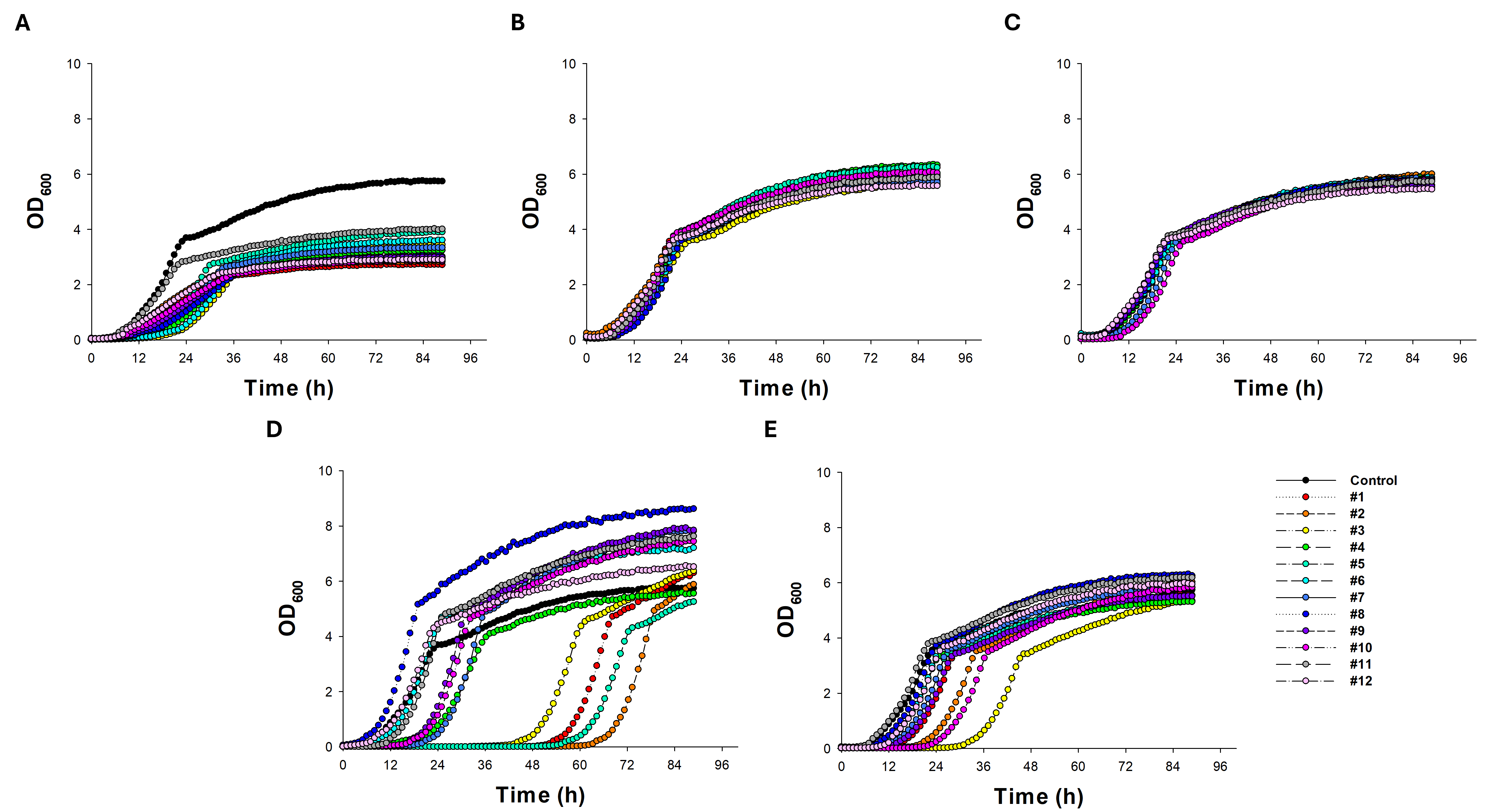
), colony #3 (yellow circle,
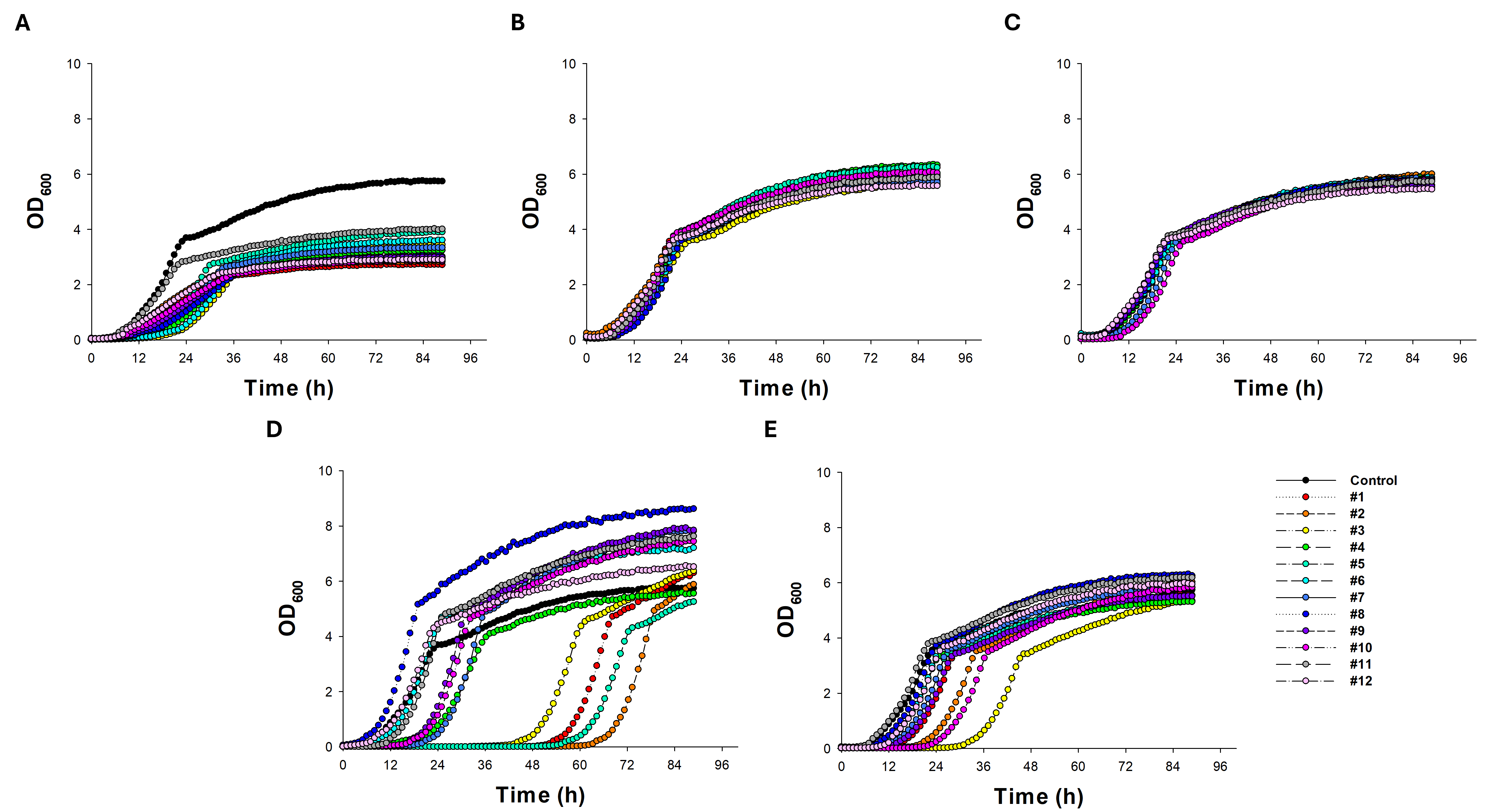
), colony #4 (green circle,
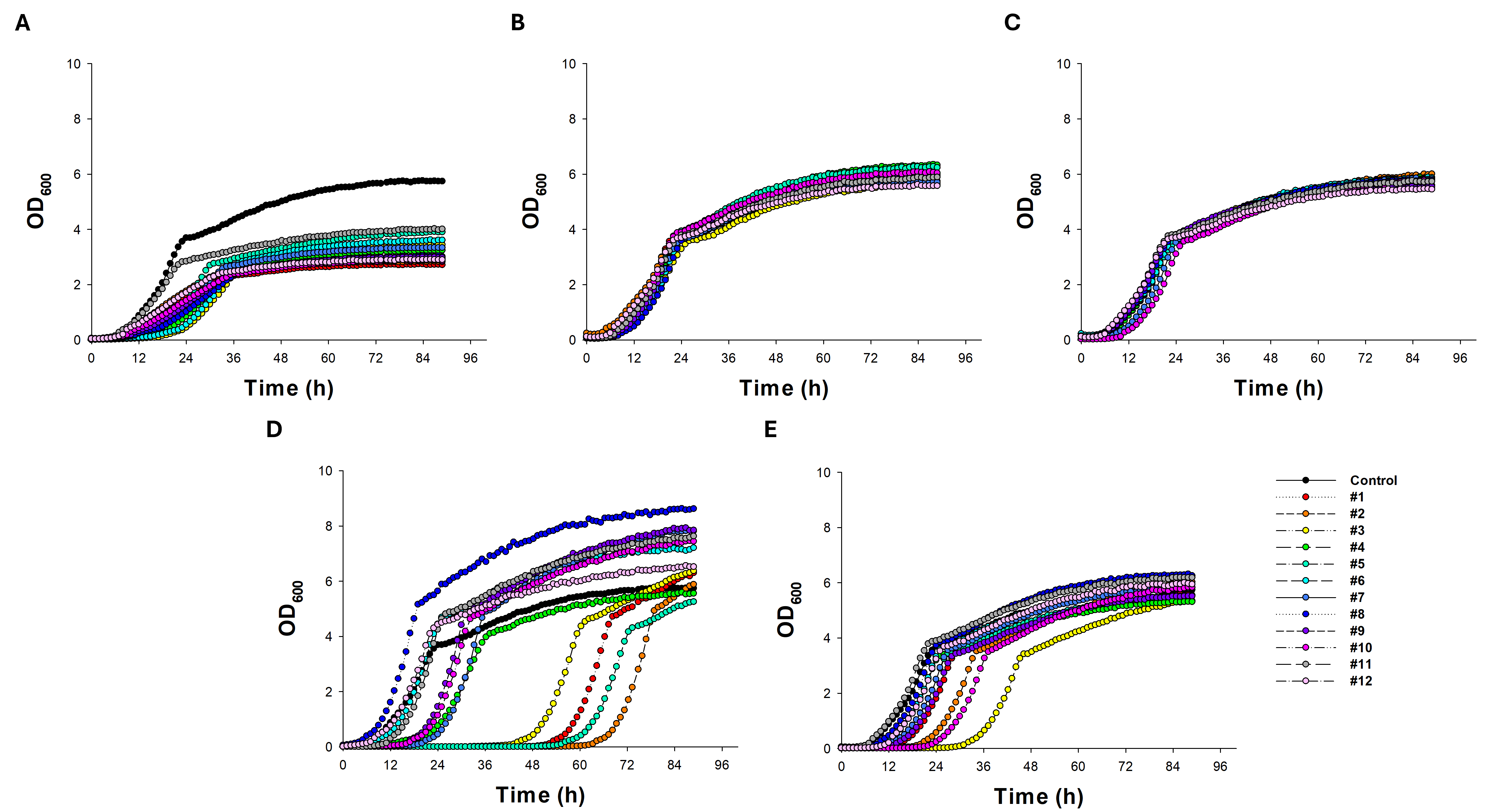
), colony #5 (teal circle,
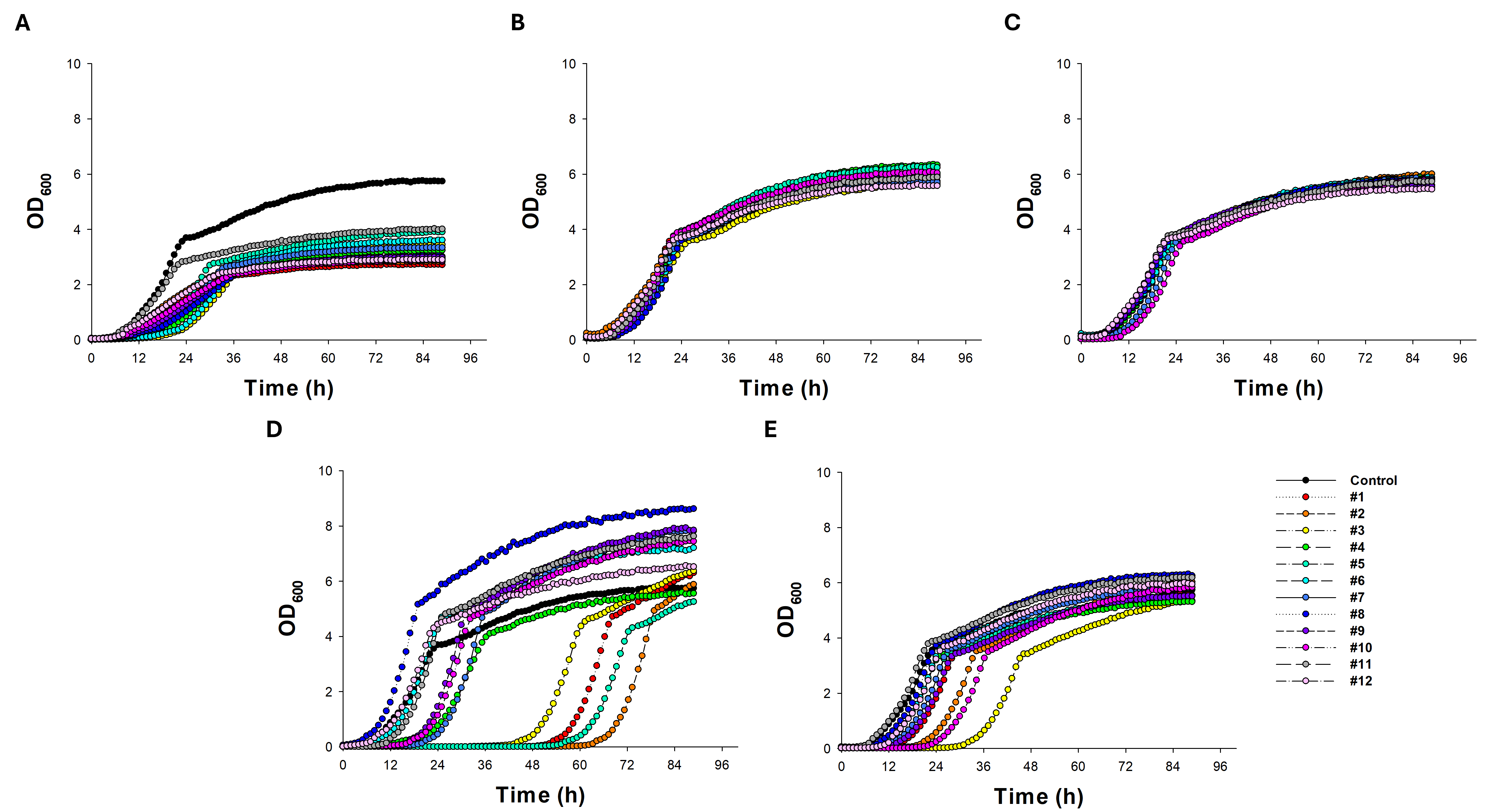
), colony #6 (Aqua circle,
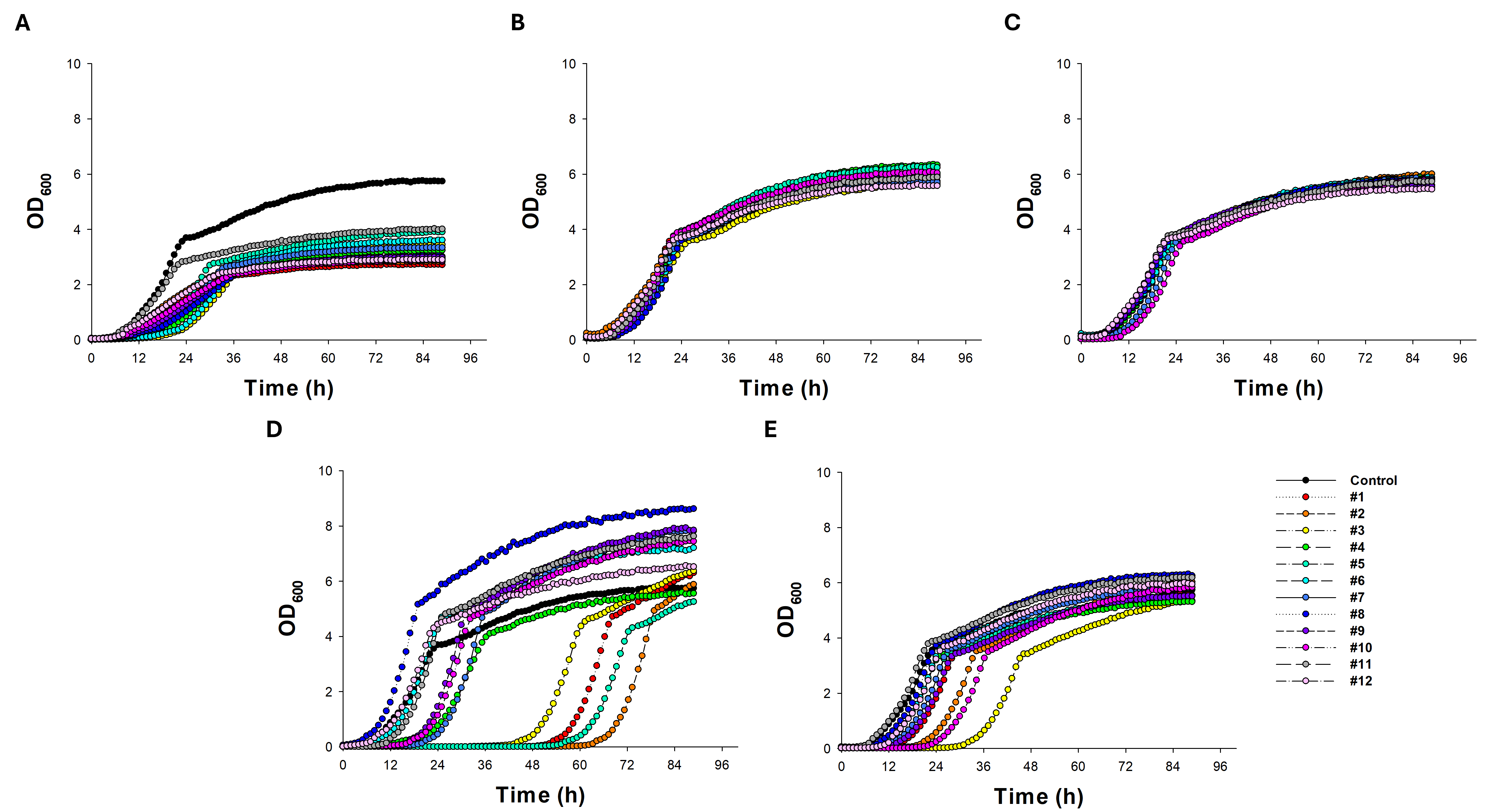
), colony #7 (light blue circle,
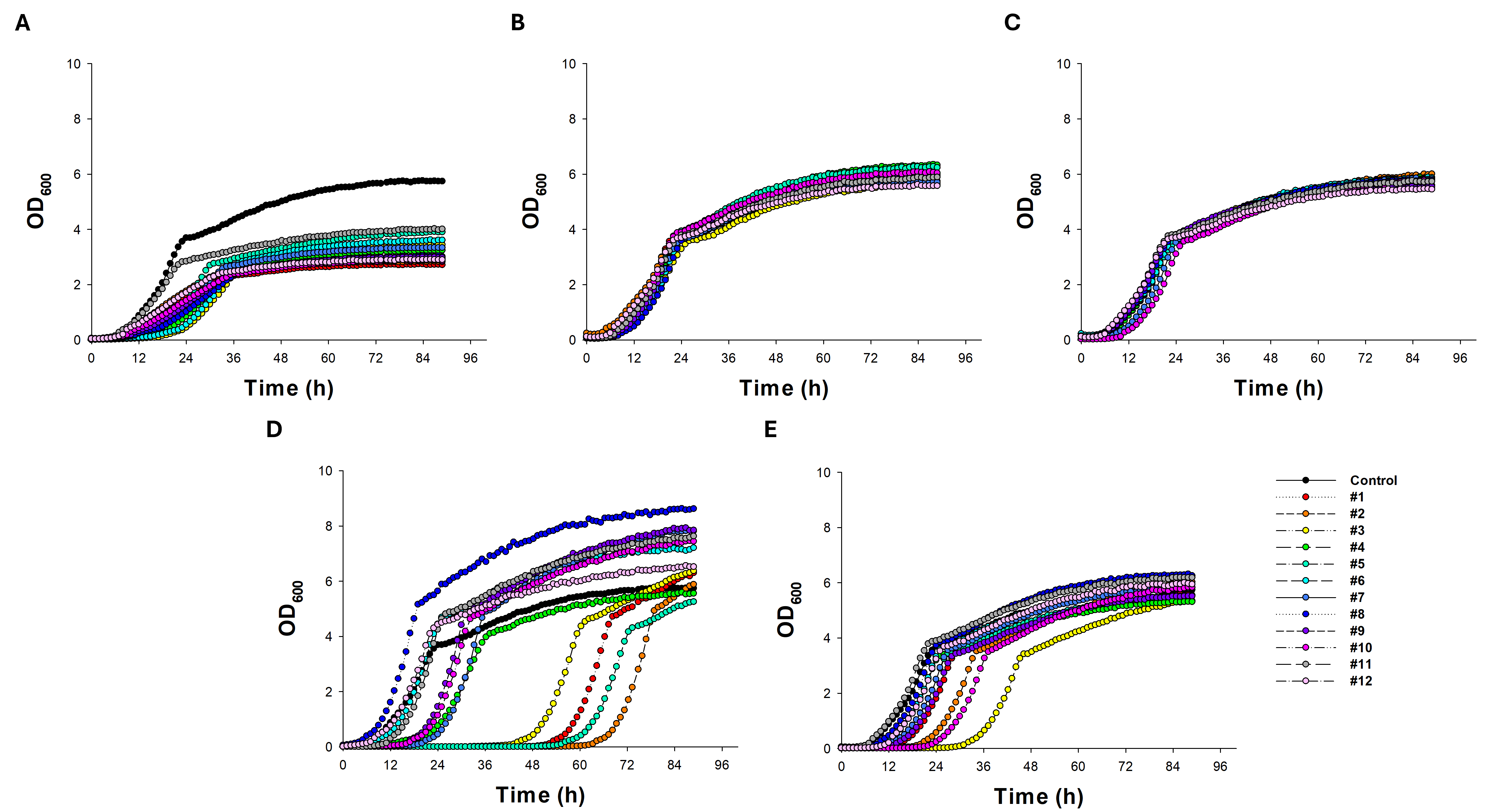
), colony #8 (dark blue circle,
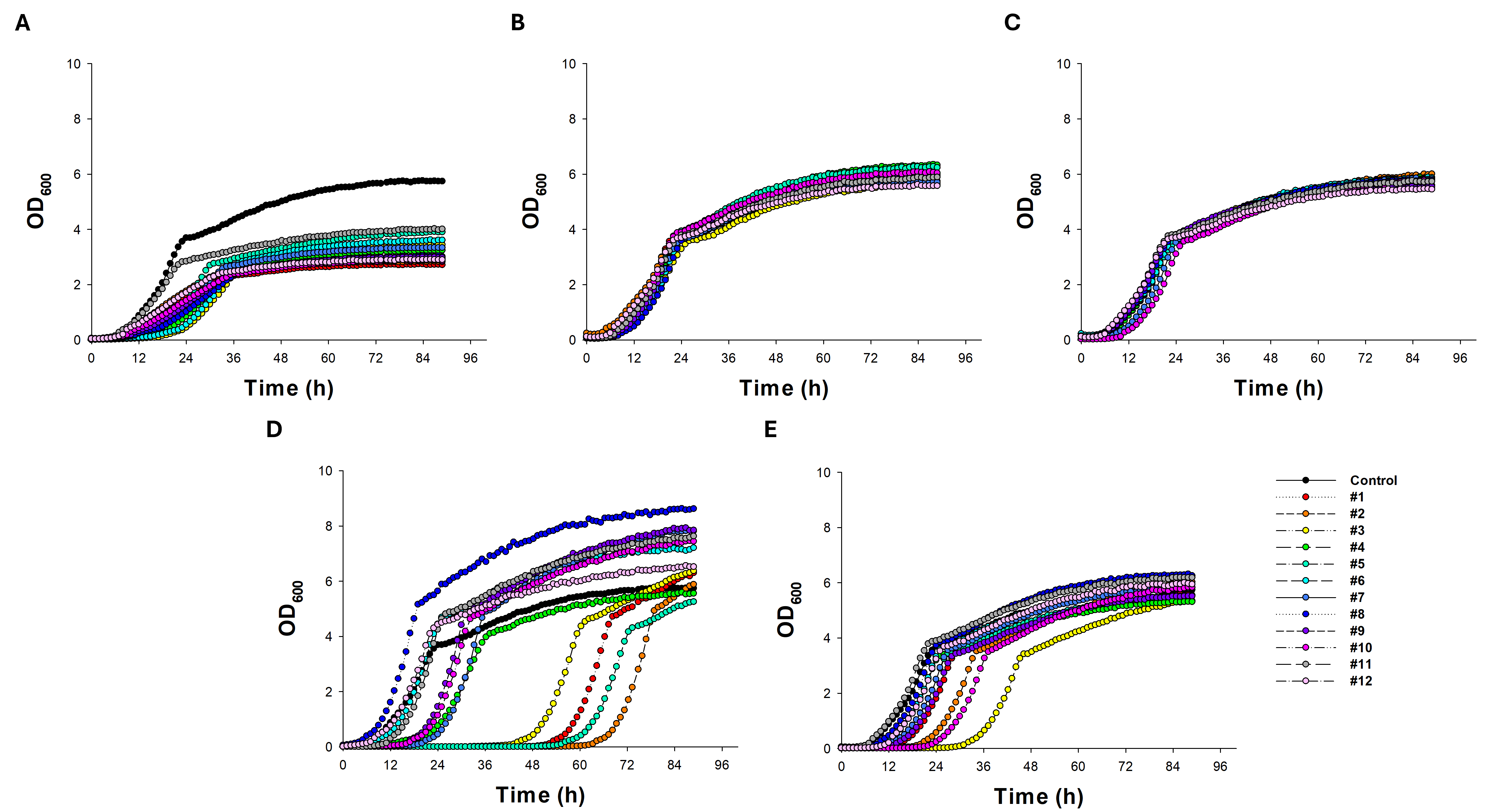
), colony #9 (purple circle,
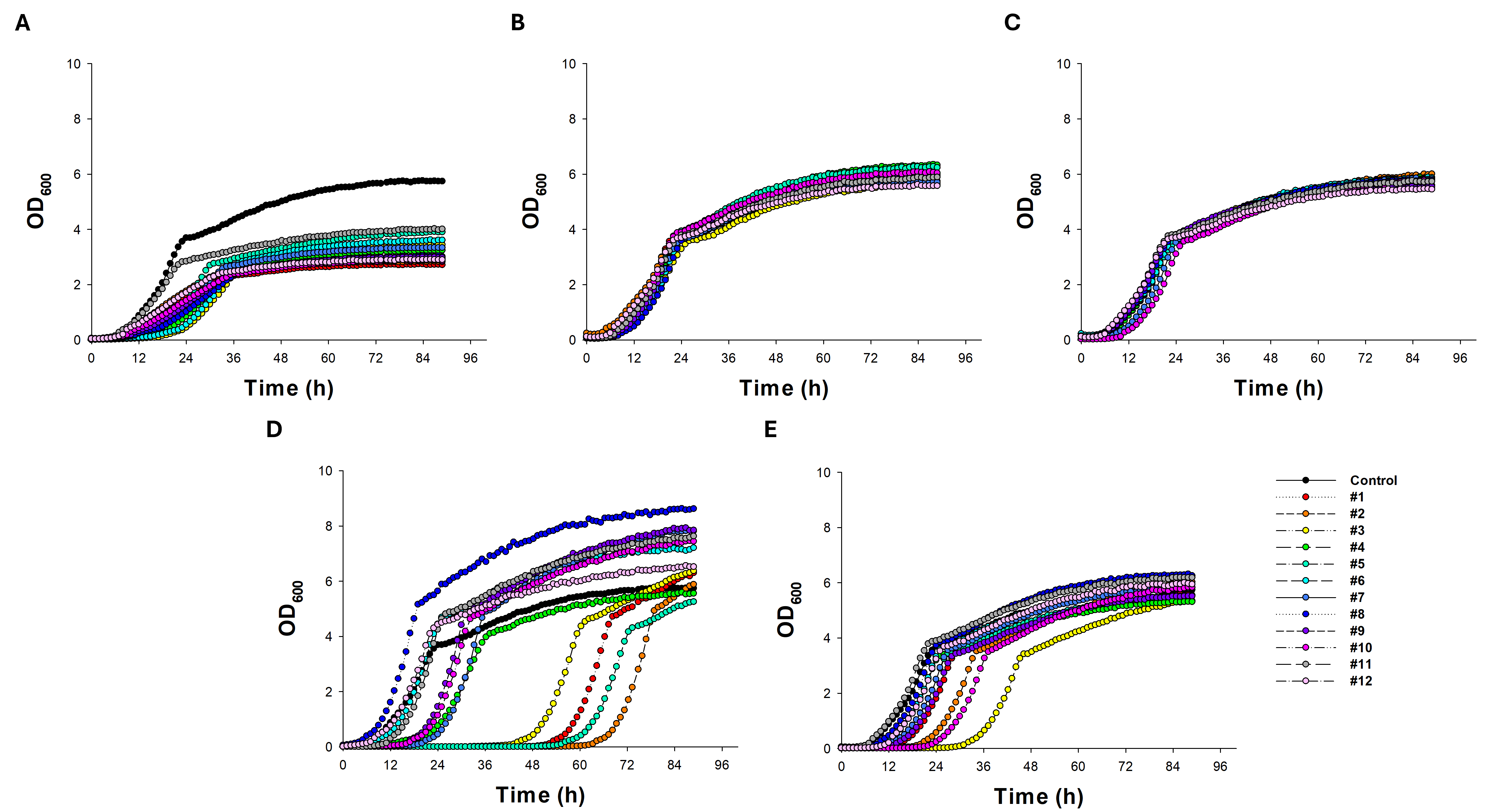
), colony #10 (pink circle,
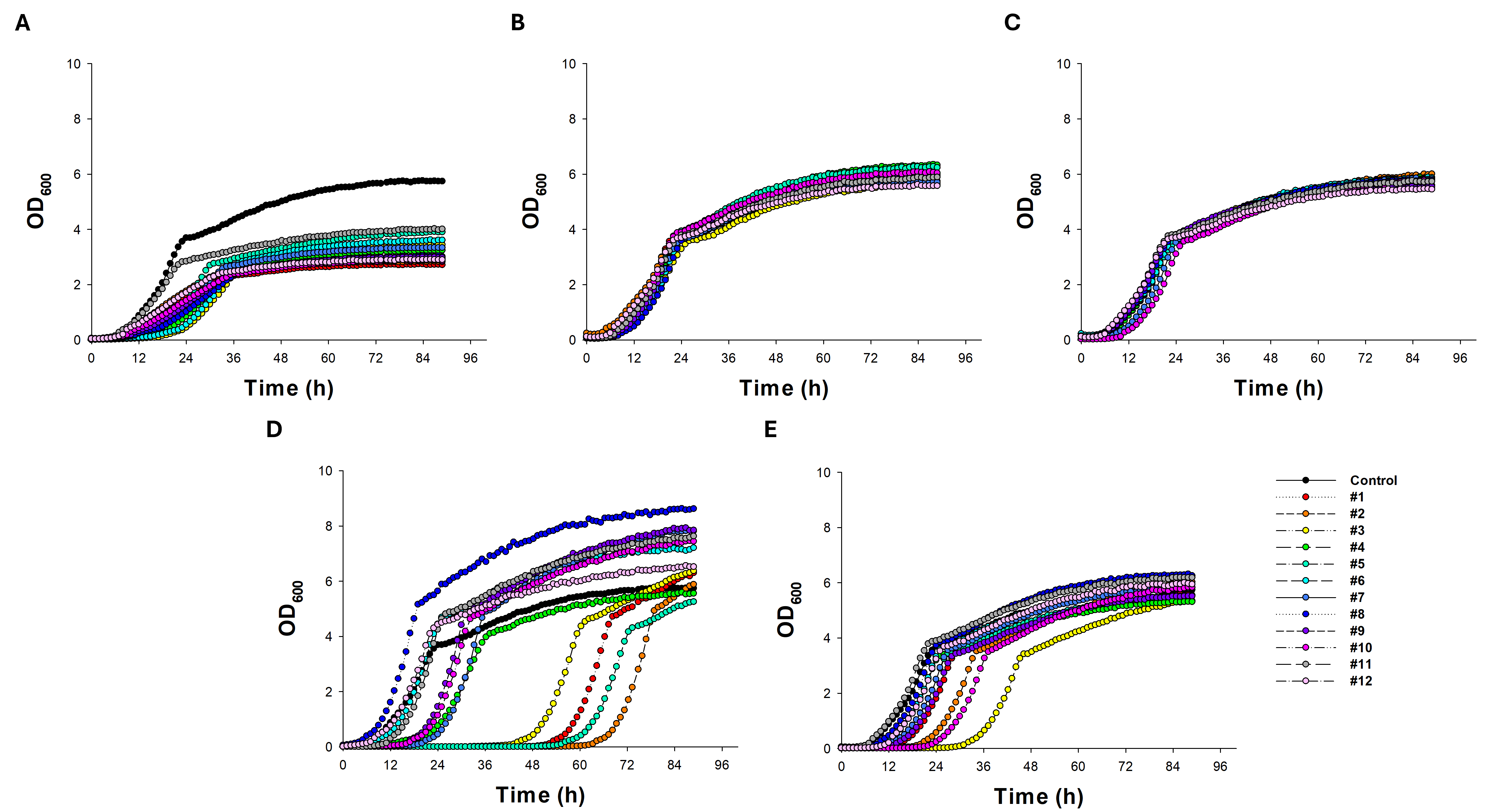
), colony #11 (grey circle,
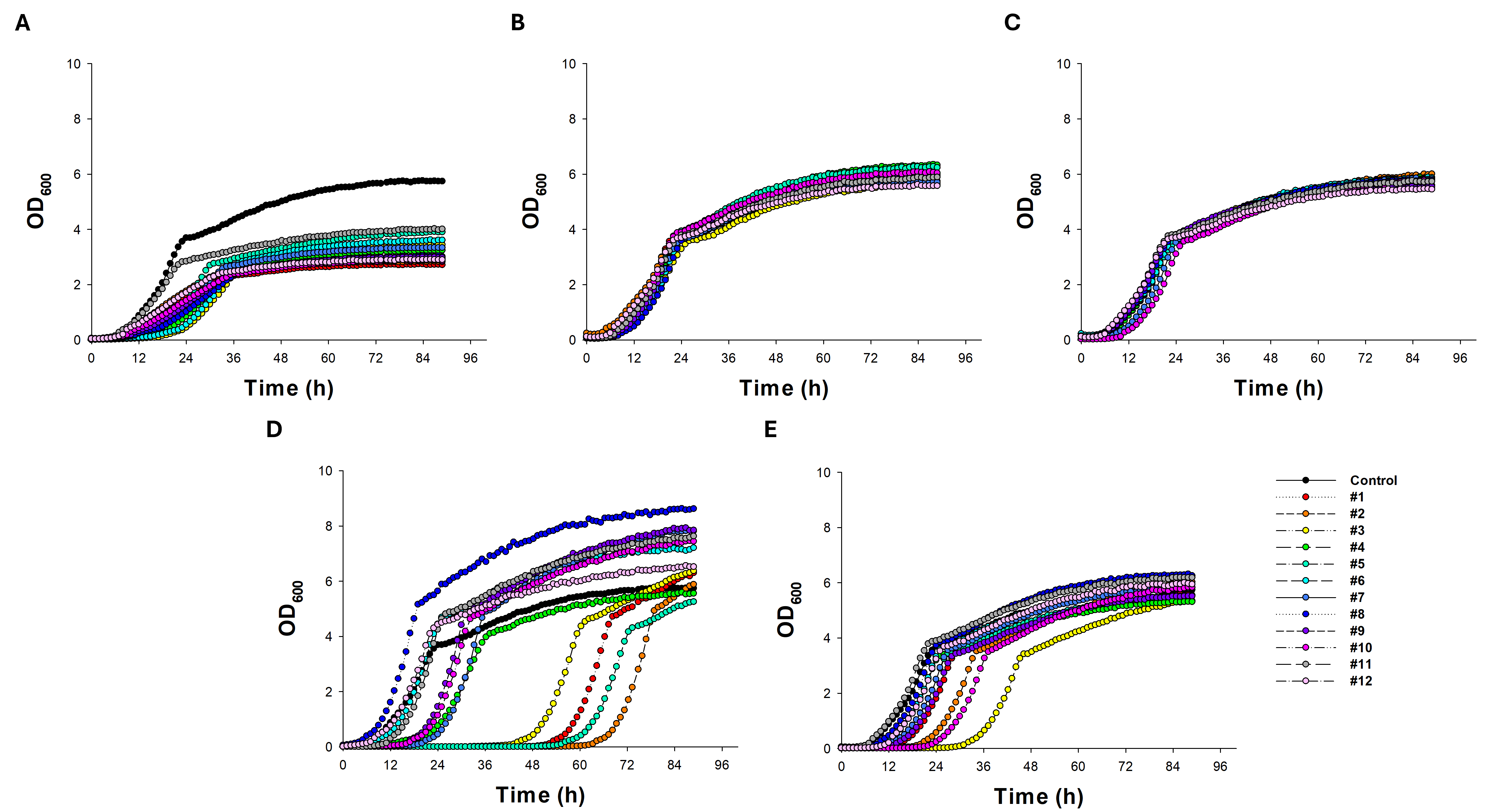
), and colony #12 (lavender circle,
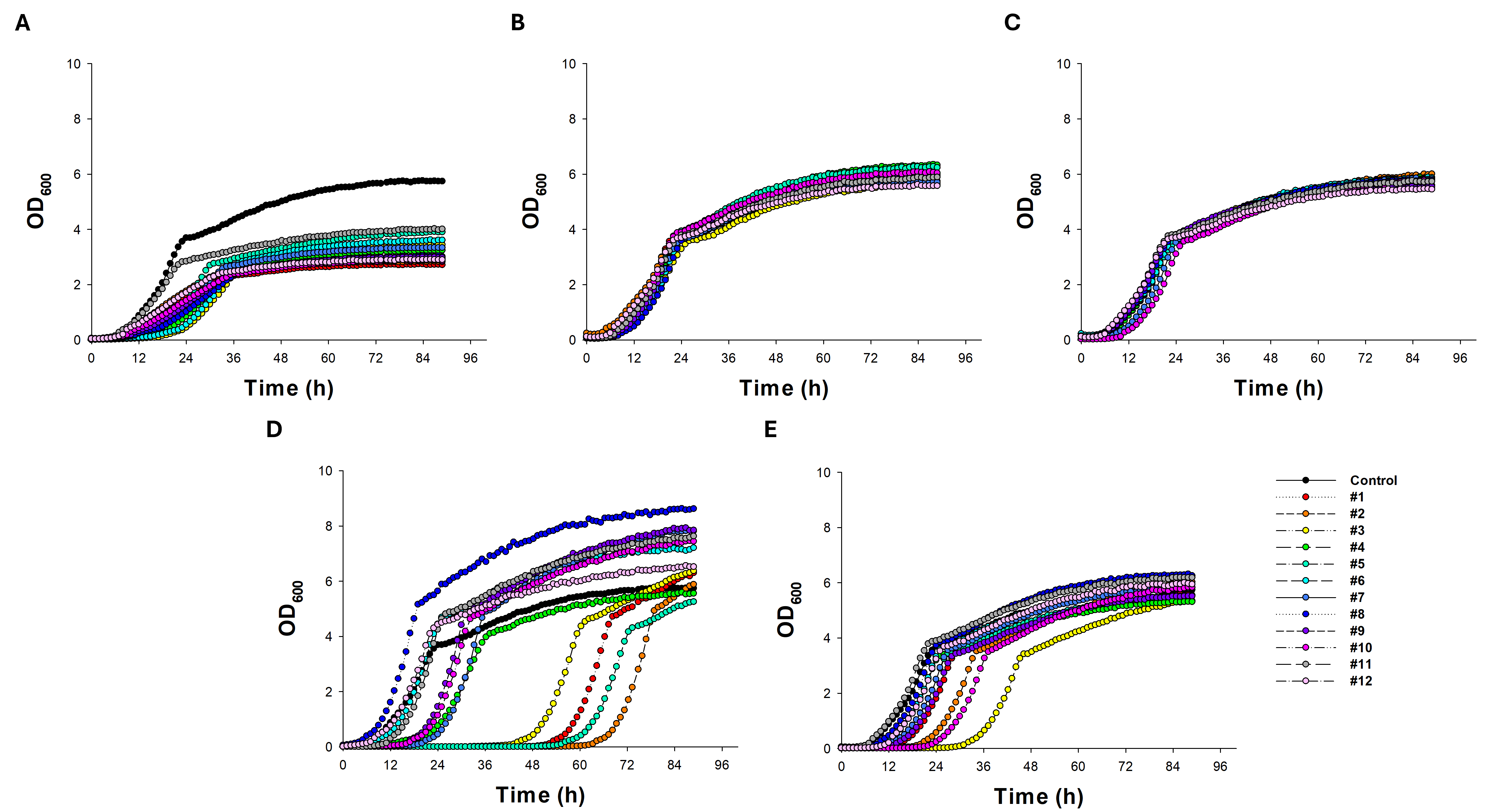
).


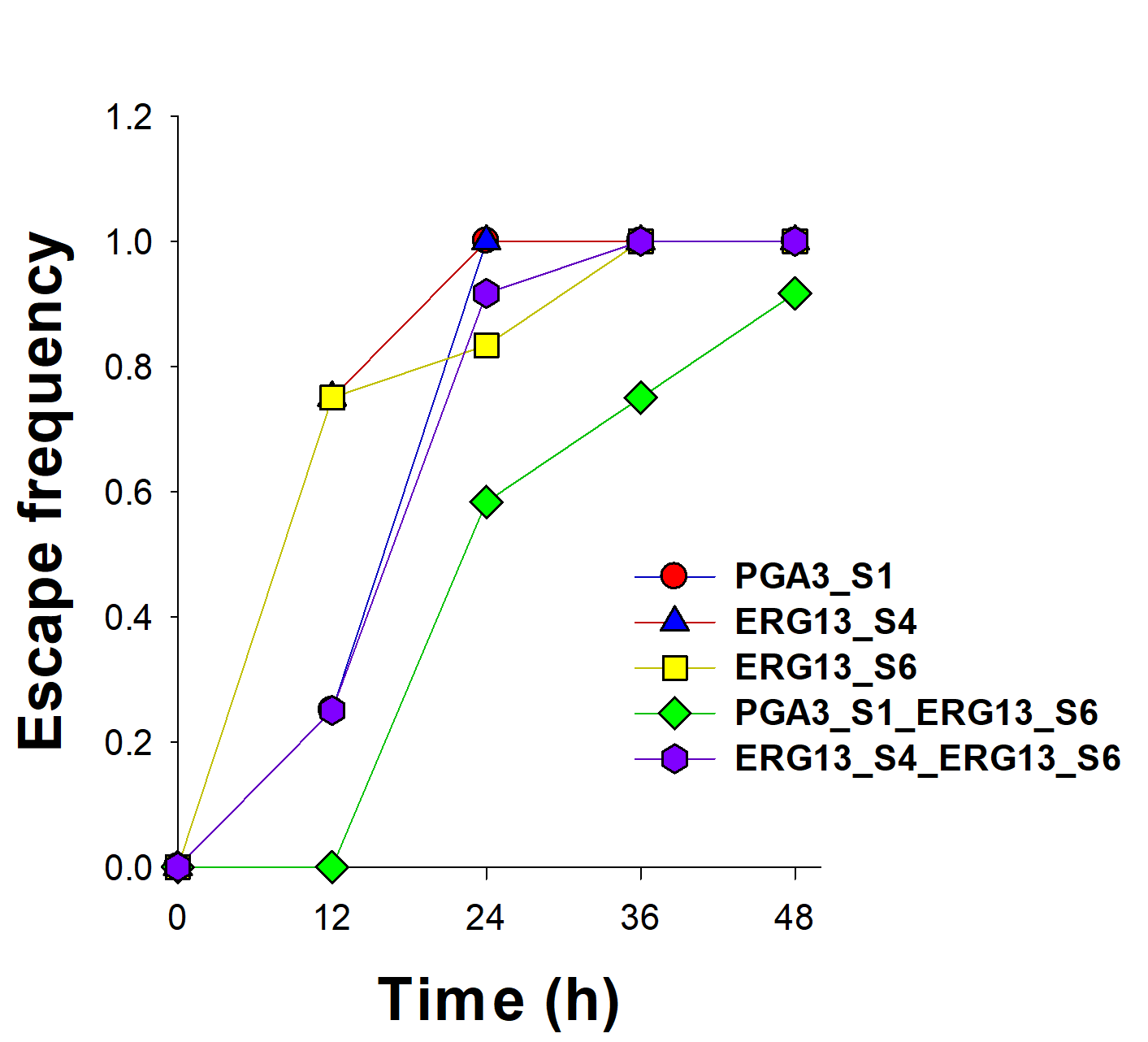


**Figure S6.** Proportion of cells that showed growth in strains expressing the KiSS systems in the absence of ATc. The cultures were inoculated from individual pre-cultures, from different colonies (n = 12) carrying the KiSS system with either single gRNA systems - PGA3_S1 (red circle,
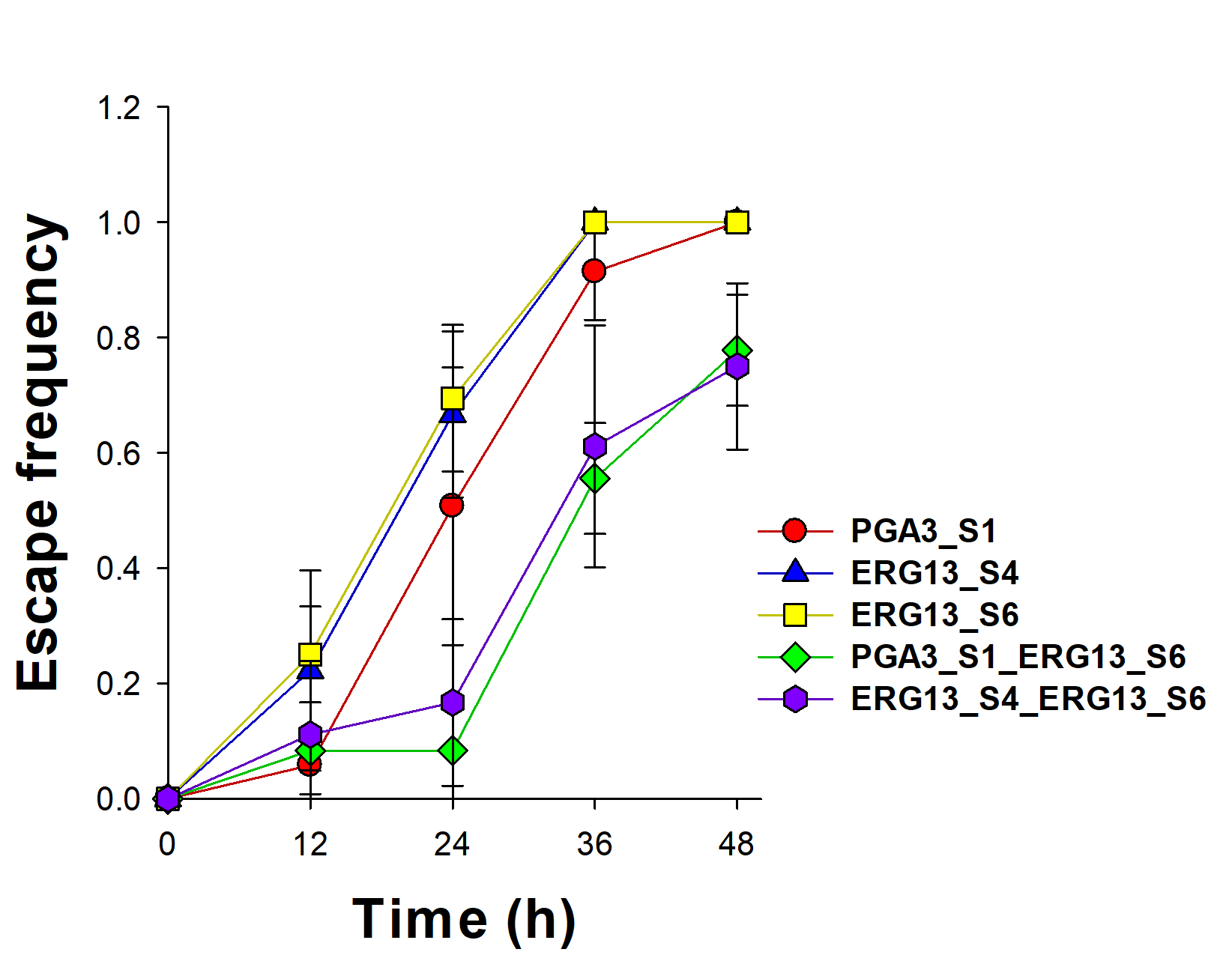
), ERG13_S4 (blue triangle,
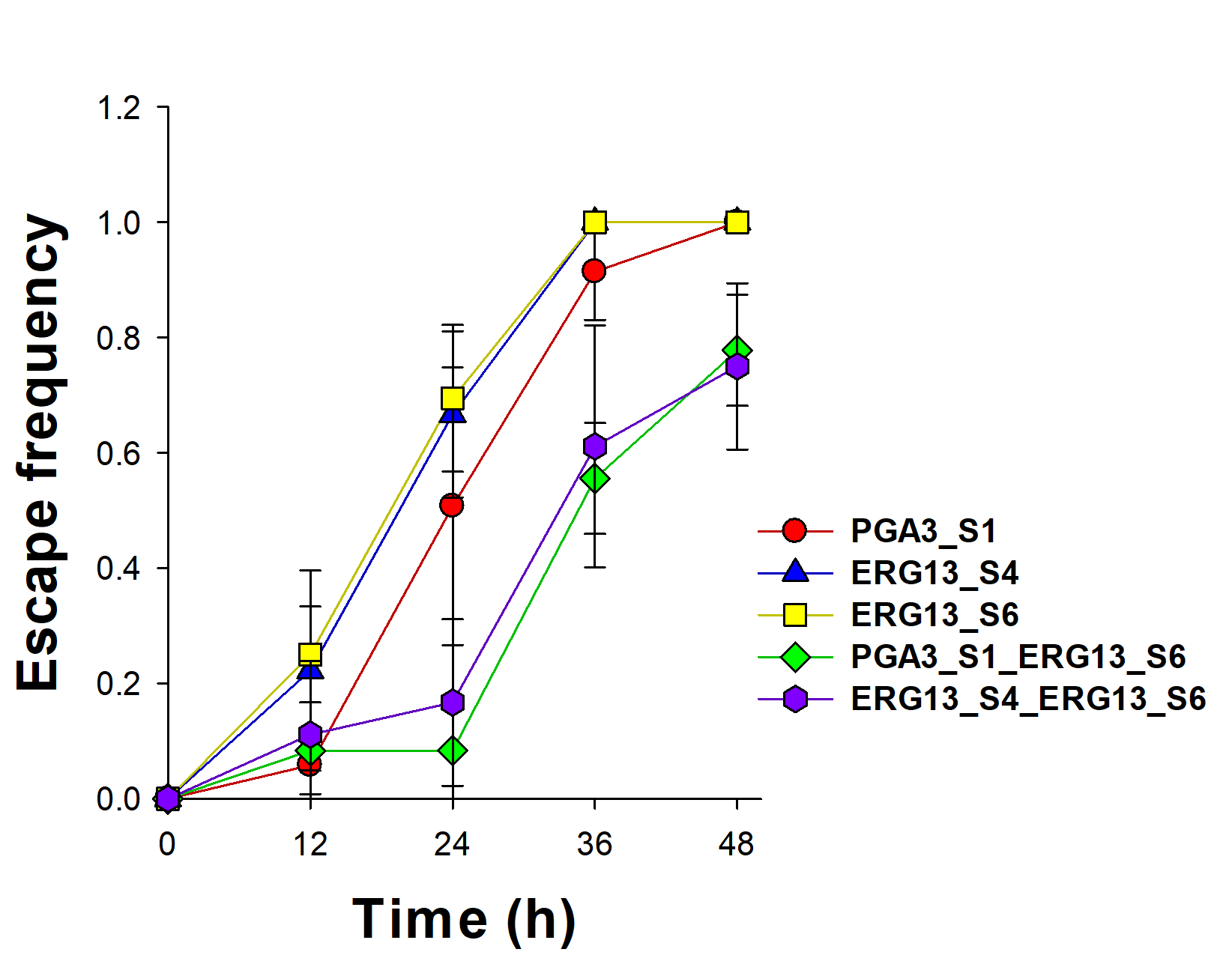
),and ERG13_S6 (yellow square,
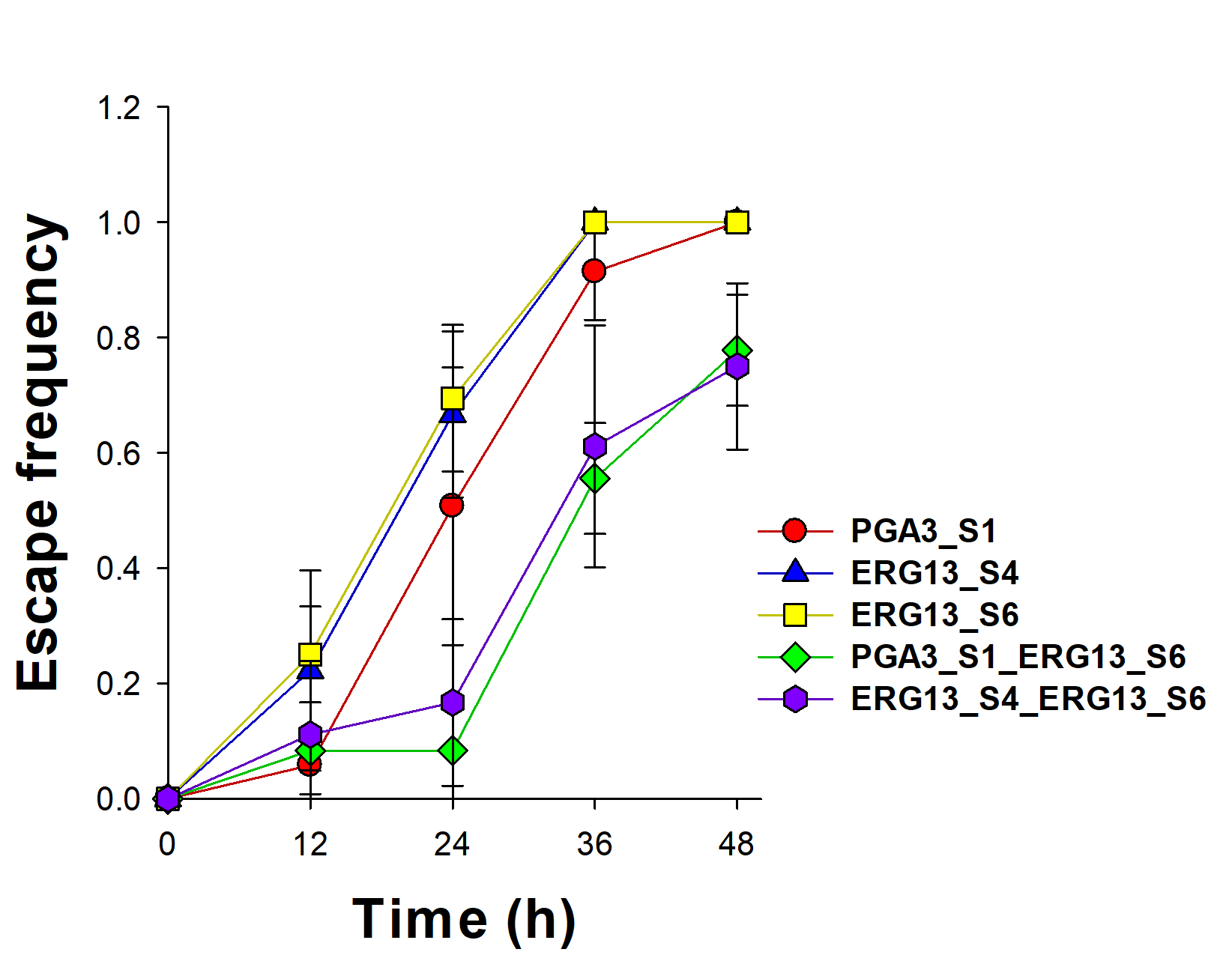
) – or dual gRNA systems -PGA3_S1_ERG13_S6 (green diamond,
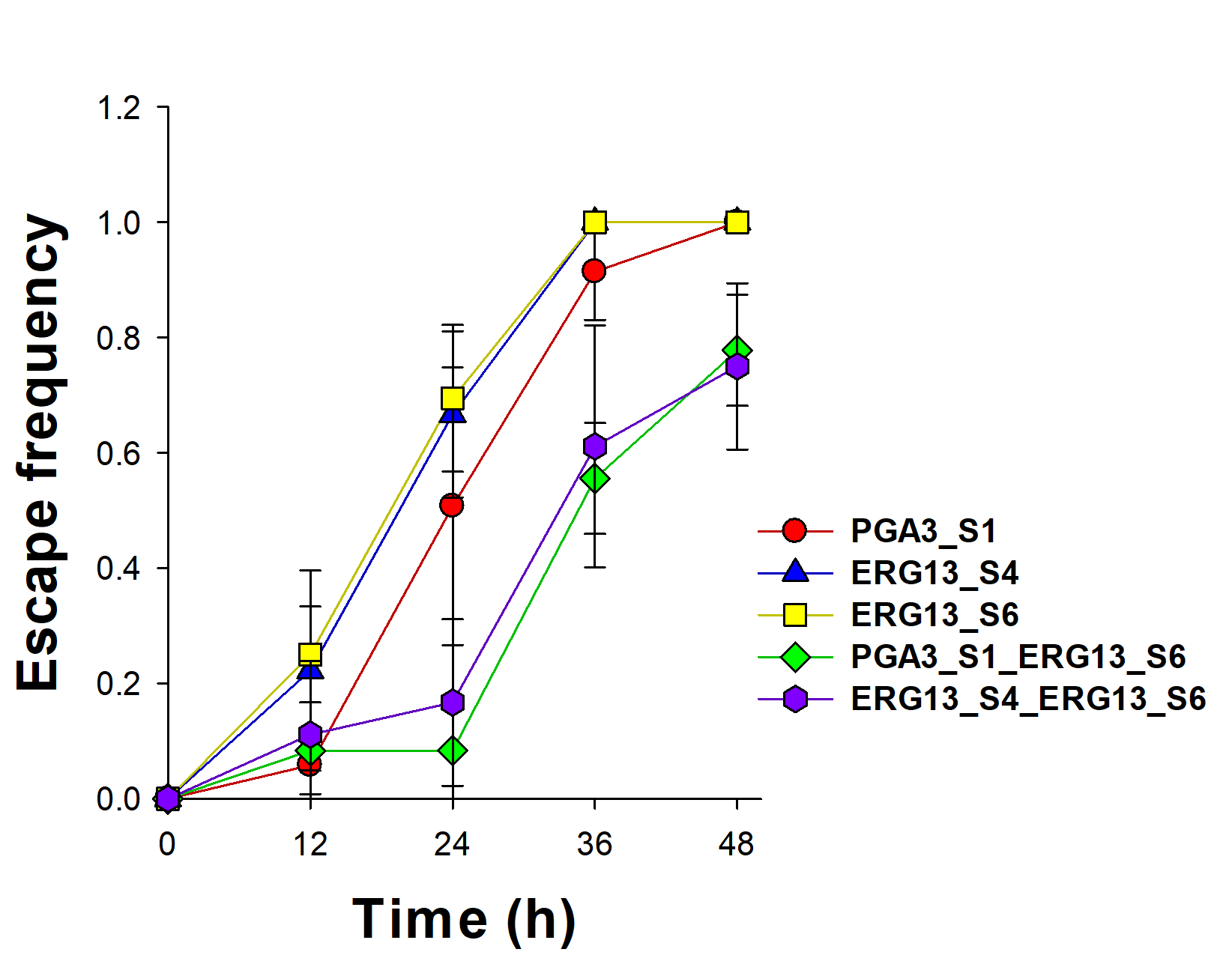
), and ERG13_S4_ERG13_S6 (purple hexagon,
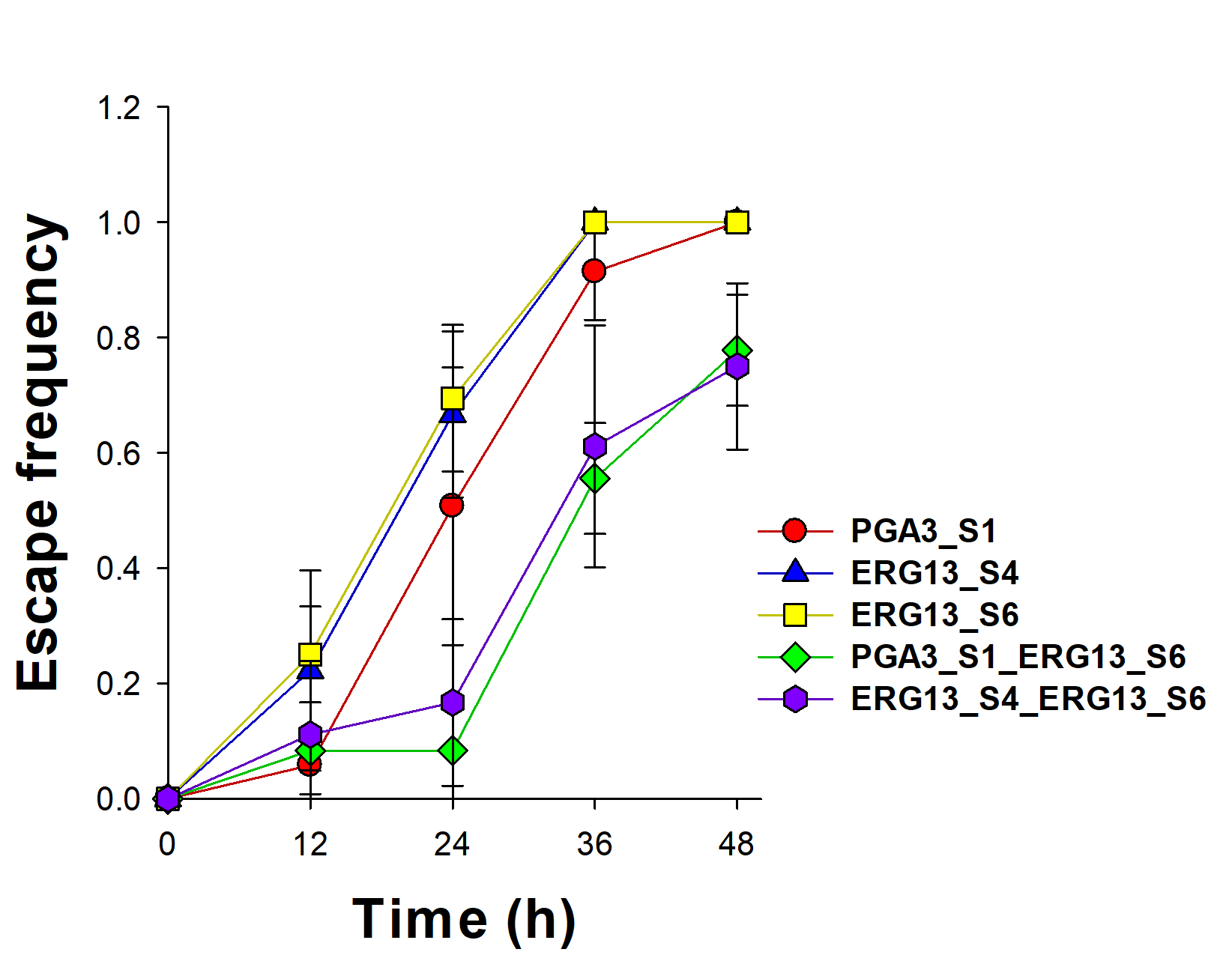
). Cultures were incubated for 90 h, without ATc.

**Table S1.** Oligos used in this study.

| **Oligo** | **sequence (5' - nnn- 3')** | **Description** |
| --- | --- | --- |
| PU_003_tetR_Fw | gcatcgtctcatcggtctcatatgatgtctagattagataaaagtaaagtgat | Forward oligo to amplify tetR |
| PU_004_tetR_Rv | atgccgtctcaggtctcaggatccttaagacccactttcacatttaag | Reverse oligo to amplify tetR |
| PU_007_sgRNA_Fw | gcatcgtctcatcggtctcatatggcggatttagctcagttgg | Forward oligo to amplify sgRNA cassette |
| PU_008_sgRNA_Rv | atgccgtctcaggtctcacagctatccacta | Reverse oligo to amplify sgRNA cassette |
| PU_023_mTurquoise2_Fw | tcatacttcttgcttttatttaaacca | Forward oligo to amplify mTurquoise2 |
| PU_024_mTurquoise2_Rv | ctaaatagattcataaactattcgttaatataaagt | Reverse oligo to amplify mTurquoise2 |
| PU_025_mCherry_Fw | tatggtgagcaagggcgag | Forward oligo to amplify mCherry |
| PU_026_mCherry_Rv | aggatttacttgtacagctcgtc | Reverse oligo to amplify mCherry |
| PU_040_backbone_sgRNA_Fw | accctgaacgagcttgcctcgtccc | Forward oligo to amplify sgRNA backbone for gibson assembly |
| PU_041_backbone_sgRNA_Rv | cagatcccccacggttatccacagaatcag | Reverse oligo to amplify sgRNA backbone for gibson assembly |
| PU_042_PRPR1_tetO_Fw | ggataaccgtgggggatctgccaattgaa | Forward oligo to amplify PRPR1_tetO for gibson assembly |
| PU_043_PRPR1_tetO_Rv | ctaaatccgcctgccaatcgcagctcc | Reverse oligo to amplify PRPR1_tetO for gibson assembly |
| PU_044_sgRNA_cassette_Fw | cgattggcaggcggatttagctcagttg | Forward oligo to amplify sgRNA cassette for gibson assembly |
| PU_045_sgRNA_cassette_Rv | actcgaactgtatccactagacagaagtttgc | Reverse oligo to amplify sgRNA cassette for gibson assembly |
| PU_046_tetR_cassette_Fw | ctagtggatacagttcgagtttatcattatcaatact | Forward oligo to amplify tetR cassette for gibson assembly |
| PU_047_tetR_cassette_Rv | gaggcaagctcgttcagggtaatatattttaaccg | Reverse oligo to amplify tetR cassette for gibson assembly |
| PU_048_Cas9_Fw | tataaaacaagatctatggacaagaag | Forward oligo to amplify Cas9 |
| PU_049_Cas9_Rv | atctcgagttaggatccttatac | Reverse oligo to amplify Cas9 |
| MM19_lv0_Fw | tgctcacatgttctttcctgcg | Forward oligo to verify insert in level 0 MoClo plasmid |
| MM20_lv0_Rv | tgttcagaacgctcggttgc | Reverse oligo to verify insert in level 0 MoClo plasmid |
| IL48_HO_lous_Fw | gcacatcgattatttgatacccc | Forward oligo to amplify HO locus |
| IL49_Ho_locus_Rv | gctcagggcactgtactg | Reverse oligo to amplify HO locus |
| ERG13_S1_FW | gactggtgccgcaagaccaaccgg | Forward oligo for sgRNA #1 targeting *ERG13* |
| ERG13_S1_RV | aaacccggttggtcttgcggcacc | Reverse oligo for sgRNA #1 targeting *ERG13* |
| PGA3_S1_FW | gactgccaagttgttcattccatc | Forward oligo for sgRNA #1 targeting *PGA3* |
| PGA3_S1_RV | aaacgatggaatgaacaacttggc | Reverse oligo for sgRNA #1 targeting *PGA3* |
| ERG13_S4_FW | gactgtggtattaaaggaagactt | Forward oligo for sgRNA #4 targeting *ERG13* |
| ERG13_S4_RV | aaacaagtcttcctttaataccac | Reverse oligo for sgRNA #4 targeting *ERG13* |
| ERG13_S5_FW | gactgtttaatagagatgcaaagg | Forward oligo for sgRNA #5 targeting *ERG13* |
| ERG13_S5_RV | aaaccctttgcatctctattaaac | Reverse oligo for sgRNA #5 targeting *ERG13* |
| ERG13_S6_FW | gactgatattgccatctacgataa | Forward oligo for sgRNA #6 targeting *ERG13* |
| ERG13_S6_RV | aaacttatcgtagatggcaatatc | Reverse oligo for sgRNA #6 targeting *ERG13* |
| URA3_S1_FW | gactaggaattactggagttagtt | Forward oligo for sgRNA #1 targeting *URA3* |
| URA3_S1_RV | aaacaactaactccagtaattcct | Reverse oligo for sgRNA #1 targeting *URA3* |
| URA3_S2_FW | gactttggcggataatgcctttag | Forward oligo for sgRNA #2 targeting *URA3* |
| URA3_S2_RV | aaacctaaaggcattatccgccaa | Reverse oligo for sgRNA #2 targeting *URA3* |
| URA3_S3_FW | gactggtgtgggtttagatgacaa | Forward oligo for sgRNA #3 targeting *URA3* |
| URA3_S3_RV | aaacttgtcatctaaacccacacc | Reverse oligo for sgRNA #3 targeting *URA3* |
| URA3_S4_FW | gactgtgcatgatattaaatagct | Forward oligo for sgRNA #4 targeting *URA3* |
| URA3_S4_RV | aaacagctatttaatatcatgcac | Reverse oligo for sgRNA #4 targeting *URA3* |
| URA3_S5_FW | gactgggtcaacagtatagaaccg | Forward oligo for sgRNA #5 targeting *URA3* |
| URA3_S5_RV | aaaccggttctatactgttgaccc | Reverse oligo for sgRNA #5 targeting *URA3* |
| BC107_107_gRNA_F | ataagaatgcggccgcgggggatctgccaattgaac | Forward oligo to amplify gRNA cassette with NotI flanked ends |
| BC107_108_gRNA_R | atagtttagcggccgctatccactagacagaagtttgcg | Reverse oligo to amplify gRNA cassette with NotI flanked ends |

**Table S2**. Plasmids used and developed in this study.

| **Plasmid name** | **Description** |
| --- | --- |
| pPU0_001_PRPR1_tetO | PRPR1-tetO (Modified RNA polymerase III promoter) |
| pPU0_004_tetR | tetR (Tetracycline regulatable repressor) |
| pPU0_006_gRNA | sgRNA cassette |
| pPU1_007_backbone_HO_integration | Backbone for HO locus-integration |
| pPU1_008_mTurquoise | Cyan fluorescent protein-encoding gene mTurquoise2 expression plasmid |
| pPU1_009_mCherry | Red fluorescent protein-encoding gene mCherry expression plasmid |
| pPU1_010_Cas9 | Cas9 expression plasmid |
| pPU1_011_tetR | tetR cassette |
| pPU1_016_backbone_sgRNA | Backbone sgRNA expression plasmid |
| pPU2_017_tetO_tetR_sgRNA | Expression plasmid (PRPR1_tetO-sgRNA with tetR) |
| pPU2_020_Cas9_FPs_integration | Cas9 and fluorescent proteins integration plasmid |
| pPU2_021_mCherry_S1 | Expression plasmid with sgRNA targeting mCherry |
| pPU2_022_mTurquoise_S1 | Expression plasmid with sgRNA targeting mTurquoise2 |
| pPU2_023_ERG13_S1 | Expression plasmid with sgRNA #1 targeting ERG13 |
| pPU2_024_PGA3_S1 | Expression plasmid with sgRNA #1 targeting PGA3 (initially named as pPU2_022_ERG13_S2) |
| pPU2_025_ERG13_S4 | Expression plasmid with sgRNA #4 targeting ERG13 |
| pPU2_026_ERG13_S5 | Expression plasmid with sgRNA #5 targeting ERG13 |
| pPU2_027_ERG13_S6 | Expression plasmid with sgRNA #6 targeting ERG13 |
| pPU2_028_URA3_S1 | Expression plasmid with sgRNA #1 targeting URA3 |
| pPU2_029_URA3_S2 | Expression plasmid with sgRNA #2 targeting URA3 |
| pPU2_030_URA3_S3 | Expression plasmid with sgRNA #3 targeting URA3 |
| pPU2_031_URA3_S4 | Expression plasmid with sgRNA #4 targeting URA3 |
| pPU2_032_URA3_S5 | Expression plasmid with sgRNA #5 targeting URA3 |
| pPU2_033_control | Expression plasmid with no sgRNA |
| pPU2_034_PGA3_S1_ERG13_S6 | Expression plasmid with sgRNA #1 and sgRNA #6 targeting PGA3 and ERG13, respectively |
| pPU2_034_ERG13_S4_ERG13_S6 | Expression plasmid with sgRNA #4 and sgRNA #6 targeting ERG13 |
| YN2_1_IL50_HOlocus | Cas9 Plasmid with sgRNA cassette for integration in the HO locus |
| pMM0_12 | Red fluorescent protein-encoding gene mCherry plasmid |

**Table S3**. Sequencing results of the KiSS strains used in this study. Target genes and the gRNA expression modules were sequenced when cell growth had resumed, after an initial growth repression.

| **Strain** | **Gene** | **Sequence** |
| --- | --- | --- |
| Control | mCherry  (partial) | atggtgagcaagggcgaggaggataacatggccatcatcaaggagttcatgcgcttcaaggtgcacatggagggctccgtggacggccacgagttcgagatcgagggcgagggcgagggccgcccctacgagggcacccagaccgccaagctgaaggtgaccaagggtggccccctgcccttcgcctgggacatcctgtcccctcagttcatgtacggctccaaggcctacgtgaagcaccccgccgacatccccgactacttgaagctgtccttccccgagggcttcaagtgggagcgcgtgatgaacttcgaggacggcggcgtggtgaccgtgacccaggactcctccctgcaggacggcgagttcatctacaaggtgaagctgcgcggcaccaacttcccctccgacggccccgtaatgcagaagaagactatgggctgggaggcctcctccgagcggatgtaccccgaggacggcgccctgaagggcgagatcaagcagaggctgaagctgaaggacggcggccactacgacgctgaggtcaagaccacctacaaggccaagaagcccgtgcagctgcccggcgcctacaacgtcaacatcaagttggacatcacctcccacaa |
| mCherry_S1 | mCherry (partial) | tccgtgagcaaggggcgaggaggataaacatgggccattcatcaaaggaggttcatgcgctttcaaggggccccatggaggggttccgtggacggccacgggtttcgaagggcgagggcccgcccccatcgaggggcacccaggaccgccaagctgaaaggtgacccaagggggggcccccctgccctttcccctgggaacatcctgtccccctcagtttaatgtacggctccaaaggcctacgtgaagcacccccccgacatccccgactacttgaagctgtcctttccccgagggcttcaagtgggagcgcgtgatgaacttcgaggacggcggcgtggtgaccgtgacccagggctcctccctgcaggacggcgagttcatctacaaggtggaagctgcgcgggcaccaacttacccctccgacggccccgtaatgcagaagaagactatgggctgggaggcctcctccgagcggatgtaccccgaggacggcgccctgaagggcgagatcaagcagaggctgaagctgaaggacggcggccactacgacgctgaggtcaagaccacctacaaggccaagaagcccgcgcagctgcccggcgcctacaacgtcaacatcaagttggacatcacctcccacaa |
| Control | mTurquoise2 (partial) | gtttctaaaggagaagaattattcactggtgttgtcccaattttggttgaattagatggtgatgttaatggtcacaaattttctgtctccggtgaaggtgaaggtgatgctacttacggtaaattgaccttaaaatttatttgtactactggtaaattgccagttccatggccaaccttagtcactactttatcttggggtgttcaatgttttgcaagatacccagatcatatgaaacaacatgactttttcaagtctgccatgccagaaggttatgttcaagaaagaactatttttttcaaagatgacggtaactacaagaccagagctgaagtcaagtttgaaggtgataccttagttaatagaatcgaattaaaaggtattgattttaaagaagatggtaacattttaggtcacaaattggaatacaattatttctctgacaatgtttacatcactgctgacaaacaaaagaatggtatcaaagctaacttcaaaattagacacaacattgaagatggtggtgttcaattagctgaccattatcaacaaaatactccaattggtgatggtccagtcttgttaccagacaaccattacttatccactcaatctaagttatccaaagatccaaacgaaaagagggaccacatggtcttgttagaatttgttactgctgctggtattaccttgggtatggatgaattgtacaaa |
| mTurquiose2_S1 | mTurquoise2 (partial) | gtttctaaaggagaagaattattcactggtgttgtcccaattttggttgaattagatggtgatggtcacaaattttctgtctccggtgaaggtgaaggtgatgctacttacggtaaattgaccttaaaatttatttgtactactggtaaattgccagttccatggccaaccttagtcactactttatcttggggtgttcaatgttttgcaagatacccagatcatatgaaacaacatgactttttcaagtctgccatgccagaaggttatgttcaagaaagaactatttttttcaaagatgacggtaactacaagaccagagctgaagtcaagtttgaaggtgataccttagttaatagaatcgaattaaaaggtattgattttaaagaagatggtaacattttaggtcacaaattggaatacaattatttctctgacaatgtttacatcactgctgacaaacaaaagaatggtatcaaagctaacttcaaaattagacacaacattgaagatggtggtgttcaattagctgaccattatcaacaaaatactccaattggtgatggtccagtcttgttaccagacaaccattacttatccactcaatctaagttatccaaagatccaaacgaaaagagggaccacatggtcttgttagaatttgttactgctgctggtattaccttgggtatggatgaattgtacaaa |
| Control | *URA3*  (partial) | atgtcgaaagctacatataaggaacgtgctgctactcatcctagtcctgttgctgccaagctatttaatatcatgcacgaaaagcaaacaaacttgtgtgcttcattggatgttcgtaccaccaaggaattactggagttagttgaagcattaggtcccaaaatttgtttactaaaaacacatgtggatatcttgactgatttttccatggagggcacagttaagccgctaaaggcattatccgccaagtacaattttttactcttcgaggacagaaaatttgctgacattggtaatacagtcaaattgcagtactctgcgggtgtatacagaatagcagaatgggcagacattacgaatgcacacggtgtggtgggcccaggtattgttagcggtttgaagcaggcggcagaagaagtaacaaaggaacctagaggccttttgatgttagcagaattgtcatgcaagggctccctatctactggagaatatactaagggtactgttgacattgcgaagagcgacaaagattttgttatcggctttattgctcaaagagacatgggtggaagagatgaaggttacgattggttgattatgacacccggtgtgggtttagatgacaagggagatgcattgggtcaacagtatagaaccgtggatgatgtggtttctacaggatctgacattattattgttggaagaggactatttgcaaagggaagggatgctaaggtagagggtgaacgttacagaaaagcaggctgggaagcatatttgagaagatgcggccagcaaaactaa |
| URA3_S1 | *URA3*  (partial) | atgtcgaaagctacatataaggaacgtgctgctactcatcctagtcctgttgctgccaagctatttaatatcatgcacgaaaagcaaacaaacttgtgtgcttcattggatgttcgtaccaccaaggaattactggagttagttgaagcattaggtcccaaaatttgtttactaaaaacacatgtggatatcttgactgatttttccatggagggcacagttaagccgctaaaggcattatccgccaagtacaattttttactcttcgaggacagaaaatttgctgacattggtaatacagtcaaattgcagtactctgcgggtgtatacagaatagcagaatgggcagacattacgaatgcacacggtgtggtgggcccaggtattgttagcggtttgaagcaggcggcagaagaagtaacaaaggaacctagaggccttttgatgttagcagaattgtcatgcaagggctccctatctactggagaatatactaagggtactgttgacattgcgaagagcgacaaagattttgttatcggctttattgctcaaagagacatgggtggaagagatgaaggttacgattggttgattatgacacccggtgtgggtttagatgacaagggagatgcattgggtcaacagtatagaaccgtggatgatgtggtttctacaggatctgacattattattgttggaagaggactatttgcaaagggaagggatgctaaggtagagggtgaacgttacagaaaagcaggctgggaagcatatttgagaagatgcggccagcaaaactaa |
| Control | *PGA3* (partial) | tatcacgccagtcctacagatcttgaatgaaatcatcaccgttcctgaagatttgacgaaagtctccctgctatatgccaatgagactgaaaatgacattctattgaaggacgaactggatgagatggccgaaaaatacccacatttccaggtccattacgtggtacactatccatccgacagatggaccggagatgtcggctacatcaccaaggaccagatgaacaggtatctgccggaatattcggaggataacagactcttgatctgtggacctgatggaatgaacaacttggcccttcaatacgctaaagaactgggctggaaggtcaattcaacgagaagttctggcgacgatcaagtc |
| PGA3_S1 | *PGA3* (partial) | tatcacgccagtcctacagatcttgaatgaaatcatcaccgttcctgaagatttgacgaaagtctccctgctatatgccaatgagactgaaaatgacattctatcgaaggacgaactggatgagatggccgaaaaatacccacatttccaggtccattacgtggtacactatccatccgacagatggaccggagatgtcggctacatcaccaaggaccagatgaacaggtatctgccggaatattcggaggataacagactcttgatctgtggaatgaacaacttggcccttcaatacgctaaagaactgggctggaaggtcaattcaacgagaagttctggcgacgatcaagtc |
| Control | *ERG13* (partial) | actaaactttgttggtgtggtattaaaggaagacttaggccgcaaaagcaacaacaattacacaatacaaacttgcaaatgactgaactaaaaaaacaaaagaccgctgaacaaaaaaccagacctcaaaatgtcggtattaaaggtatccaaatttacatcccaactcaatgtgtcaaccaatctgagctagagaaatttgatggcgtttctcaaggtaaatacacaattggtctgggccaaaccaacatgtcttttgtcaatgacagagaagatatctactcgatgtccctaactgttttgtctaagttgatcaagagttacaacatcgacaccaacaaaattggtagattagaagtcggtactgaaactctgattgacaagtccaagtctgtcaagtctgtcttgatgcaattgtttggtgaaaacactgacgtcgaaggtattgacacgcttaatgcctgttacggtggtaccaacgcgttgttcaactctttgaactggattgaatctaacgcatgggatggtagagacgccattgtagtttgcggtgatattgccatctacgataagggtgccgcaagaccaaccggtggtgccggtactgttgctatgtggatcggtcctgatgctc |
| ERG13_S4 | *ERG13* (partial) | actaaactttgttggtgtggtattaaaggaagggcttaggccgcaaaagcaacaacaattacacaatacaaacttgcaaatgactgaactaaaaaaacaaaagaccgctgaacaaaaaaccagacctcaaaatgtcggtattaaaggtatccaaatttacatcccaactcaatgtgtcaaccaatctgagctagagaaatttgatggcgtttctcaaggtaaatacacaattggtctgggccaaaccaacatgtcttttgtcaatgacagagaagatatctactcgatgtccctaactgttttgtctaagttgatcaagagttacaacatcgacaccaacaaaattggtagattagaagtcggtactgaaactctgattgacaagtccaagtctgtcaagtctgtcttgatgcaattgtttggtgaaaacactgacgtcgaaggtattgacacgcttaatgcctgttacggtggtaccaacgcgttgttcaactctttgaactggattgaatctaacgcatgggatggtagagacgccattgtagtttgcggtgatattgccatctacgataagggtgccgcaagaccaaccggtggtgccggtactgttgctatgtggatcggtcctgatgctc |
| ERG13_S6 | *ERG13* (partial) | actaaactttgttggtgtggtattaaaggaagacttaggccgcaaaagcaacaacaattacacaatacaaacttgcaaatgactgaactaaaaaaacaaaagaccgctgaacaaaaaaccagacctcaaaatgtcggtattaaaggtatccaaatttacatcccaactcaatgtgtcaaccaatctgagctagagaaatttgatggcgtttctcaaggtaaatacacaattggtctgggccaaaccaacatgtcttttgtcaatgacagagaagatatctactcgatgtccctaactgttttgtctaagttgatcaagagttacaacatcgacaccaacaaaattggtagattagaagtcggtactgaaactctgattgacaagtccaagtctgtcaagtctgtcttgatgcaattgtttggtgaaaacactgacgtcgaaggtattgacacgcttaatgcctgttacggtggtaccaacgcgttgttcaactctttgaactggattgaatctaacgcatgggatggtagagacgccattgtagtttgcggtgatattgccatctacggtaagggtgccgcaagacc |
| Control | pPU2_024_PGA3_S1 (partial) | cctcttcttattggccggctgtctctatactcccctatagtctgtttcttttcgtttcgattgtccctatcagtgatagagatggcgcacatggtacgctgtggtgctcgcggctgggaacgaaactctgggagctgcgattggcaggcggatttagctcagttgggagagcgccagactgaagaaaaacttcggtcaagtcatctggaggtcctgtgttcgatccacagaattcgcagatggccggcatggtcccagcctcctcgctggcgccggctgggcaacaccttcgggtggcgaatgggactgccaagttgttcattccatcgttttagagctagaaatagcaagttaaaataaggctagtccgttatcaacttgaaaaagtggcaccgagtcggtgctttt |
| PGA3_S1 | pPU2_024_PGA3_S1 (partial) | cctcttcttattggccggctgtctctatactcccctatagtctgtttcttttcgtttcgattgtccctatcagtgatagagatggcgcacatggtacgctgtggtgctcgcggctgggaacgaaactctgggagctgcgattggcaggcggatttagctcagttgggagagcgccaaactgaagaaaaacttcggtcaagtcatctggaggtcctgtgttcaatccacagaattcgcagatggccggcatggtcccagcctcctcgctggcgccggctgggcaacaccttcgggtggcgaatgggactgccaagttgttcatttccatcgttttagagctagaaatagcaagttaaaataaggctagtccgttatcaactttgaaaaagtggcaccgagtctgtgctttt |
| Control | pPU2_025_ERG13_S4 (partial) | cctcttcttattggccggctgtctctatactcccctatagtctgtttcttttcgtttcgattgtccctatcagtgatagagatggcgcacatggtacgctgtggtgctcgcggctgggaacgaaactctgggagctgcgattggcaggcggatttagctcagttgggagagcgccagactgaagaaaaacttcggtcaagtcatctggaggtcctgtgttcgatccacagaattcgcagatggccggcatggtcccagcctcctcgctggcgccggctgggcaacaccttcgggtggcgaatgggactgtggtattaaaggaagacttgttttagagctagaaatagcaagttaaaataaggctagtccgttatcaacttgaaaaagtggcaccgagtcggtgctttt |
| ERG13_S4 | pPU2_025_ERG13_S4 (partial) | cctcttcttatcggccggctgtctctatactcccctatagtctgtttcttttcgtttcgattgtccctatcagtgatagagatggcgcacatggtacgctgtggtgctcgcggctgggaacgaaactctgggagctgcgattggcaggcggatttagctcagttgggagagcgccaaactgaaaaaaaacttcggtcaagtcatctggaggtcctgtgttcaatccacagaattcgcagatggccggcatggtcccagcctcctcgctggcgccggctgggcaacaccttcgggtggcgaatgggactgtggtattaaaggaagacttgttttagagctagaaatagcaagttaaaataaggctagtccgttatcaacttgaaaaagtggcaccgagtctgtgctttt |
| Control | pPU2_027_ERG13_S6 (partial) | cctcttcttattggccggctgtctctatactcccctatagtctgtttcttttcgtttcgattgtccctatcagtgatagagatggcgcacatggtacgctgtggtgctcgcggctgggaacgaaactctgggagctgcgattggcaggcggatttagctcagttgggagagcgccagactgaagaaaaacttcggtcaagtcatctggaggtcctgtgttcgatccacagaattcgcagatggccggcatggtcccagcctcctcgctggcgccggctgggcaacaccttcgggtggcgaatgggactttatcgtagatggcaatatcgttttagagctagaaatagcaagttaaaataaggctagtccgttatcaacttgaaaaagtggcaccgagtcggtgctttt |
| ERG13_S6 | pPU2_027_ERG13_S6 (partial) | actaactttattgggccggctagtctctatactcccctatagtctagtttactttttcgtttcgattgtccctatcagtgatagagatggcgcacatggtacgctgtggtgctcgcggctgggaacgaaactctgggagctgcgattggcaggcggatttagctcagttgggagagcgccaaactgaaaaaaaacttcggtcaagtcatctggaggtcctgtgttcaatccacagaattcgcagatggccggcatggtcccagcctcctcgctggcgccggctggtgcaacacctttcgggtggcgaatgggtactgatatttgccatctaccgataagtttttagagctagaaatagcaagtttaaaataaggctaagtccgtttatcaactttgaaaaaggtggcaccgtagtctgtgcttta |

Samples for sequencing were taken after 20 h of cultivation in medium lacking ATc (mCherry_S1, mTurquiose_S1, and URA3_S1, see Figure 2A), or after 36 h (PGA3_S1 #3 see Figure S6A) or 45 h (ERG13_S4 #1 and ERG13_S6 #1, see Figure S3E and F) of cultivation in medium supplemented with 10 µg/ml ATc (Figure S3).
